# Medicinal Plants for the Treatment of Local Tissue Damage Induced by Snake Venoms: An Overview from Traditional Use to Pharmacological Evidence

**DOI:** 10.1155/2017/5748256

**Published:** 2017-08-21

**Authors:** Juliana Félix-Silva, Arnóbio Antônio Silva-Junior, Silvana Maria Zucolotto, Matheus de Freitas Fernandes-Pedrosa

**Affiliations:** ^1^Laboratório de Tecnologia & Biotecnologia Farmacêutica (TecBioFar), Faculdade de Farmácia, Universidade Federal do Rio Grande do Norte (UFRN), Natal, RN, Brazil; ^2^Grupo de Pesquisa em Produtos Naturais Bioativos (PNBio), Laboratório de Farmacognosia, Faculdade de Farmácia, Universidade Federal do Rio Grande do Norte (UFRN), Natal, RN, Brazil

## Abstract

Snakebites are a serious problem in public health due to their high morbimortality. Most of snake venoms produce intense local tissue damage, which could lead to temporary or permanent disability in victims. The available specific treatment is the antivenom serum therapy, whose effectiveness is reduced against these effects. Thus, the search for complementary alternatives for snakebite treatment is relevant. There are several reports of the popular use of medicinal plants against snakebites worldwide. In recent years, many studies have been published giving pharmacological evidence of benefits of several vegetal species against local effects induced by a broad range of snake venoms, including inhibitory potential against hyaluronidase, phospholipase, proteolytic, hemorrhagic, myotoxic, and edematogenic activities. In this context, this review aimed to provide an updated overview of medicinal plants used popularly as antiophidic agents and discuss the main species with pharmacological studies supporting the uses, with emphasis on plants inhibiting local effects of snake envenomation. The present review provides an updated scenario and insights into future research aiming at validation of medicinal plants as antiophidic agents and strengthens the potentiality of ethnopharmacology as a tool for design of potent inhibitors and/or development of herbal medicines against venom toxins, especially local tissue damage.

## 1. Introduction

Snakebites are a serious public health problem in many regions around the world, particularly in Africa, Asia, Latin America, and parts of Oceania [1]. Conservative data indicate that, worldwide, there are between 1.2 and 5.5 million snakebites every year, leading to 25,000 to 125,000 deaths [2]. Despite its significant impact on human health, this condition remains largely neglected by national and international health authorities, funding agencies, pharmaceutical companies, patients' organizations, and health advocacy groups [1]. Thus, snake envenomation is included since 2009 in World Health Organization (WHO) list of Neglected Tropical Diseases (NTDs) [3]. Envenoming and deaths resulting from snakebites are a particularly important public health problem in the rural tropics. Populations in these regions experience high morbidity and mortality because of poor access to health services, which are often suboptimal, as well as other NTDs, which are associated with poverty [3, 4].

Snakes with major clinical importance belong to the families Elapidae (African and Asian cobras, Asian kraits, African mambas, American coral snakes, Australian and New Guinean venomous snakes, and sea snakes) and Viperidae (Old World vipers, American rattlesnakes and pit vipers, and Asian pit vipers) [5]. After production, snake venom is injected in the victim via tubular or channeled fangs [6]. Biochemically, venoms are complex mixtures of pharmacologically active proteins and polypeptides, acting in concert to help in immobilizing the prey [7]. The most common toxins in snake venoms are snake venom metalloproteinases (SVMPs), phospholipases A_2_ (PLA_2_s), snake venom serine proteinases (SVSPs), acetylcholinesterase (AChE), L-amino acid oxidases (LAAOs), nucleotidases, and snake venom hyaluronidases (SVHs) [7].

Biological properties of snake venom components are peculiar to each species, but in general, the main clinical effects of snake envenomation are immediate and prominent local tissue damage (including myonecrosis, dermonecrosis, hemorrhage, and edema), coagulation disorders (consumption coagulopathy and spontaneous systemic bleeding), cardiovascular alterations (hypotension, hypovolemic shock, and myocardial damage), renal alterations (which could evolve into acute kidney injure), neurotoxic action (descending paralysis, progressing from ptosis and external ophthalmoplegia to bulbar, respiratory muscle, and total flaccid paralysis), generalized rhabdomyolysis with myoglobinuria, and intravascular haemolysis [5, 8].

The only available specific treatment is the antivenom serum therapy, which consists of a pool of neutralizing immunoglobulins, or immunoglobulin fragments, purified from the plasma of animals hyperimmunized against snake venoms or specific toxins. Its effectiveness consists in its ability to provide to the patient antibodies with a high affinity to snake venom, aiming to eliminate the toxins responsible for toxicity of the envenoming, mitigating the progress of toxic effects induced by snake venom components [9]. However, the antivenom has some limitations, such as poor ability to treat local effects, risk of immunological reactions, high cost, and difficult access in some regions [8–10]. If antivenom administration is initiated rapidly after envenomation, neutralization of systemic effects is usually achieved successfully; however, neutralization of local tissue damage is more difficult [8]. Furthermore, the availability and accessibility of antivenoms is limited in many regions, such as Sub-Saharan Africa, Asia, and, to a lesser extent, Latin America, which could aggravate even more this picture [1]. Thus, this inability to treat local effects, as well as the increased time between accident and treatment, is the main reason for the temporary or permanent disability observed in many victims, which can lead to serious social, economic, and health negative impacts, given that most victims live in rural areas [3].

In this context, the search for complementary therapies to treat snakebites is relevant and medicinal plants could be highlighted as a rich source of natural inhibitors and pharmacologically active compounds [6, 11–13]. There are several reports of the popular use of medicinal plants against snakebites around the world, especially in tropical and subtropical regions such as Asia, Africa, and South America [14, 15]. The rural and tribal people living in remote areas greatly depend on folk medicines for the treatment of bites from any venomous creatures [16]. The use of medicinal plants against snakebites is a historical practice throughout the human history, and this knowledge has been transferred among the rural communities from generation after generation [17]. Nowadays, these herbal antidotes used in folk traditional medicine gained much attention by toxinologists worldwide as a tool for design of potent inhibitors against snake venom toxins. The potential advantages of antiophidic plants are their possible low cost, easy access, stability at room temperature, and ability to neutralize a broad spectrum of toxins, including the local tissue damage [12, 15–17].

So, the objective of this review is to provide an updated overview of medicinal plants used popularly as antiophidic and discuss the main species with pharmacological studies supporting the uses, with emphasis on plants inhibiting local effects of snake envenomation, since this is a critical effect of snake venoms that could lead to relevant sequel to victims. A review of the main botanical families popularly used as antiophidic is presented, including the main species and forms of popular use of them. Then, studies supporting their popular use are discussed, as well as the advantages of this kind of approach for treatment of snake venom accident.

## 2. Methodology

An extensive review of the literature was undertaken in different scientific sources, such as PubMed (https://www.ncbi.nlm.nih.gov/pubmed), Science Direct (http://www.sciencedirect.com/), Scopus (https://www.scopus.com/), Web of Science (http://www.webofknowledge.com/),* “Literatura Latino-Americana e do Caribe em Ciências da Saúde”* (LILACS) (http://lilacs.bvsalud.org/), Scientific Electronic Library Online (SciELO) (http://www.scielo.org/), Google Scholar (https://scholar.google.com.br/), Cochrane Library (http://www.cochranelibrary.com/), and Centre for Reviews and Dissemination (CRD) (http://www.crd.york.ac.uk/CRDWeb).

The study database included original articles published in peer-reviewed journals, as well as books, thesis, dissertations, patents, and other reports covering antiophidic plants (ethnopharmacological surveys, original articles, or reviews), dated until December 2016. For the online search, where applicable, the following search strategy was employed: (“plant” OR “plants” OR “plant extract” OR “vegetal” OR “vegetal species” OR “vegetal extract” OR “traditional medicine” OR “alternative medicine” OR “complementary therapy” OR “natural medicine” OR “ethnopharmacology” OR “ethnobotany” OR “herbal medicine” OR “herb” OR “herbs” OR “decoction” OR “tea” OR “infusion” OR “macerate”) AND (“snake venom” OR “snake” OR “snakes” OR “snakebite” OR “snakebites” OR “antivenom” OR “antivenoms” OR “anti-venom” OR “anti-venoms” OR “antivenin” OR “antivenins” OR “anti-venin” OR “anti-venins” OR “antiophidian” OR “antiophidic” OR “snake envenomation” OR “antitoxin” OR “antitoxins” OR “snake antidote” OR “snake antidotes” OR “snake venom neutralization” OR “snake venom inhibition” OR “snake toxins inhibition” OR “snake toxins neutralization” OR “viper” OR “viperidae” OR “crotalinae” OR “viperinae” OR “elapidae” OR “pit-viper” OR “bothrops” OR “jararaca” OR “crotalus” OR “micrurus” OR “lachesis” OR “cobra” OR “naja” OR “bitis” OR “vipera” OR “daboia” OR “trimeresus”).

All abstracts and/or full-text data were considered, without language restriction. Then, the publications covering ethnobotanical and/or pharmacological studies of antiophidic plants were selected and carefully analyzed. With the information gathered in these studies, the actual scenario of the use of plants against snake venom was pointed out. Main botanical families used, main countries where antiophidic plants are reported, and mode of use mostly employed in folk medicine were described. Regarding studies of pharmacological evidence, the snake species that were most studied, which plant species were tested and presented positive results, correlating with those species that also presented record of ethnopharmacological use, were also reported.

The accepted botanical name of each medicinal plant listed was confirmed in at least 2 botanical databases among the following ones: Flora do Brasil (http://www.floradobrasil.jbrj.gov.br/), Tropicos (http://www.tropicos.org/), The Plant List (http://www.theplantlist.org/), and NCBI Taxonomy Browser (https://www.ncbi.nlm.nih.gov/taxonomy). In some cases, where the same species was considered as different ones (different synonyms used) in different papers, the accepted name according to the botanical databases mentioned above was used in the present review, bringing the synonym used in the original work between parenthesis.

## 3. Medicinal Plants as a Popular Source of Antidotes for Snakebites: Traditional Use

According to the literature search performed, a lot of ethnopharmacological studies showing medicinal plants claimed as antiophidic were found. A summary of these vegetal species can be observed in Table 1.

**Figure 1:**
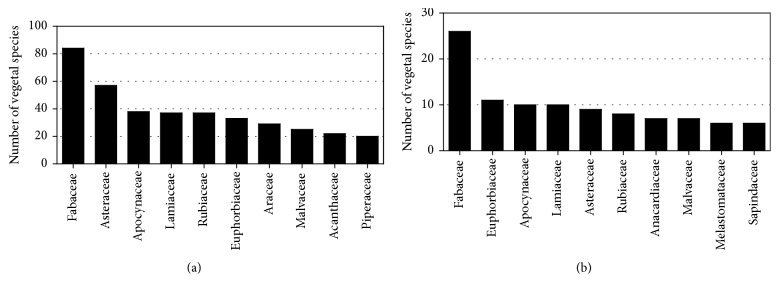
“Hot families” with antiophidic potential. Main related botanical families in ethnopharmacological surveys as antiophidic (a) and main botanical families that were evaluated in antiophidic assay (inhibition of local tissue damage) and presented positive results (b).

Along our survey were found 150 botanical families containing plants with reputation against snakebites, among which the most cited ones were the families Fabaceae, Asteraceae, Apocynaceae, Lamiaceae, Rubiaceae, Euphorbiaceae, Araceae, Malvaceae, and Acanthaceae (Figure 1(a)). In a cross-cultural comparison of medicinal floras used against snakebites, Molander et al. [80] identified five countries with a high number of antiophidic plants and representing different cultures, geography, and floristic zones: Brazil, Nicaragua, Nepal, China, and South Africa. From these countries, some “hot” families were identified, which were Apocynaceae, Lamiaceae, Rubiaceae, and Zingiberaceae [80], similar to the present review, except for the Zingiberaceae family which was not so reported in our survey.

**Figure 2:**
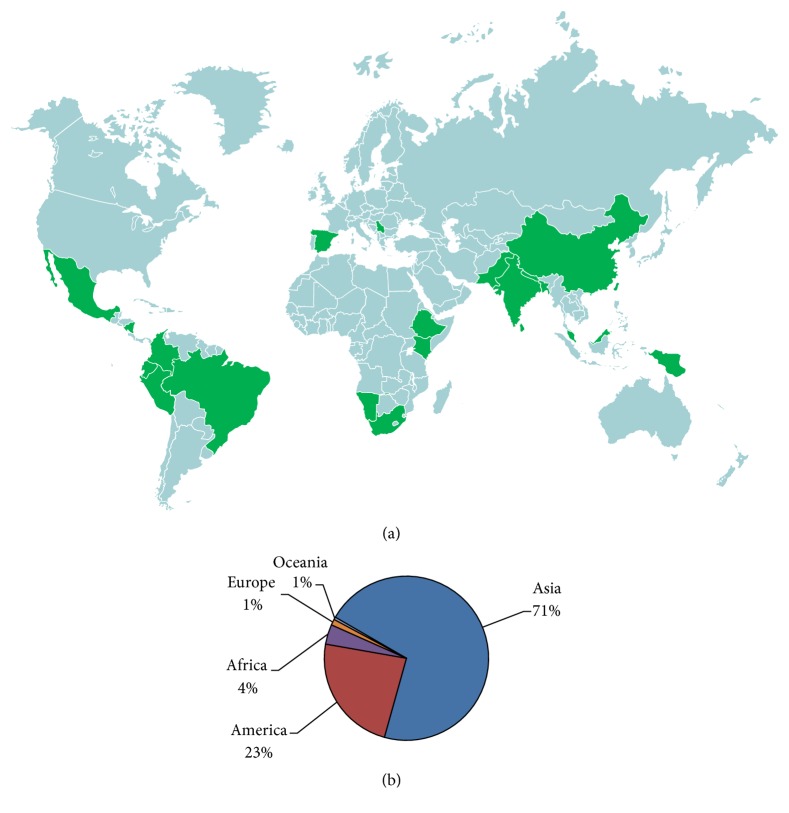
Distribution of medicinal plants used against snakebite around the world. World map highlighting the countries where antiophidic plants were related in ethnopharmacological surveys (a) and number of vegetal species per continent (b).

Medicinal plants with reputation against snakebites are found all over the world, especially in tropical or subtropical regions of Asia, Americas, and Africa (Figure 2). This fact may be associated with richness of flora of these regions, as well as with relative need of complementary therapies to treat snakebites, considering geographical features that could limit the distribution and availability of the antivenoms in these areas.

**Figure 3:**
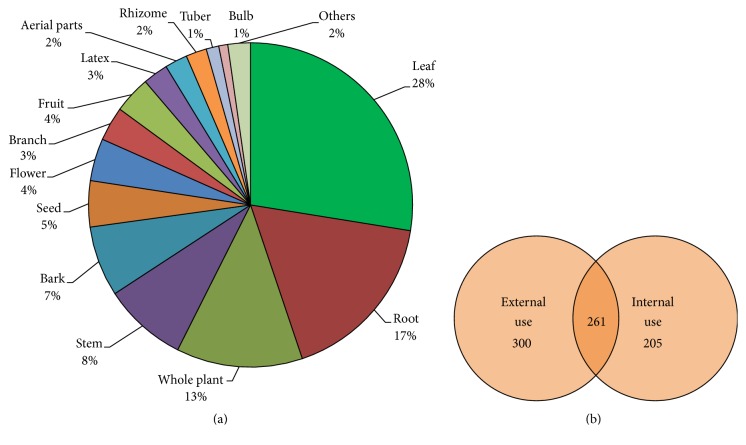
Mode of utilization of antiophidic plants reported by folk medicine. Main plant parts used (a) and Venn diagram showing the number of species enlisted having external use, internal use, or both (b).

As observed in Figure 3(a), leaves and roots are the parts of plants most used in folk medicine. Regarding the mode of use, the most frequent one is the topical application of the vegetal products directly on the place of the bite (Figure 3(b)). This is interesting especially in snake venoms that cause serious local tissue damage, such as* Bothrops* and* Daboia* species. Since these snakes produce intense local tissue damage, which has a very rapid onset, a topical treatment could be interesting for a rapid inhibitory action. On the other hand, interestingly, the use of some plant species is made by internal and external routes simultaneously, while for some other species the route of administration could be chosen among internal or external use. However, since in several cases this information is not clear, this differentiation was not considered in data tables. Regarding the mode of preparation, in general, paste and decoction were the most cited forms of use. However, for most of the plants enlisted, the information of mode of preparation was missing or confusing.

It is important to emphasize that these plant species, in addition to their use as antiophidic agents, present a series of another popular uses (data not shown) in popular medicine, mainly anti-inflammatory activity. For example,* Jatropha gossypiifolia* (Euphorbiaceae) has antiophidic, anti-inflammatory, analgesic, antipyretic, healing, and antihemorrhagic uses, among others [81].

## 4. Antivenom Activities of Extracts of Medicinal Plants against Snake Venom Induced Local Tissue Damage

### 4.1. General Aspects

Until date, according to our database, only a few numbers (less than 20%) of the species with reputation against snakebites were tested in preclinical assays with different snake venoms, which shows that there is still a great road for the study of antiophidic plants. From these tested plants which have popular use documented in our database, more than a half (almost 60%) showed positive results, which shows that in fact ethnobotany could be a good tool for bioprospecting of plants with antiophidic activity. In addition, the fact that among the tested vegetal species very significative results were obtained strongly suggests the potentiality of these natural products as a future source for development of snake venom inhibitors.

The plant families with most vegetal species showing positive results in antiophidic tests were Fabaceae, Euphorbiaceae, Apocynaceae, Lamiaceae, Asteraceae, Malvaceae, Melastomaceae, and Sapindaceae (Figure 1(b)). Crossing the data of popular use (Figure 1(a)) and of positive activity (Figure 1(b)), we can highlight these families as “hot” ones, that is, families that might be preferred or prioritized in studies searching for antiophidic plants.

Snakes from the genus* Naja*,* Bothrops*, and* Bitis* were the most evaluated ones in these antiophidic assays. However, although* Naja* and* Bitis* comprise a large fraction of the studies, virtually most of them are only* in vitro* studies, dealing with the* in vitro* enzymatic inhibition of classes of venom toxins relevant to local tissue damage, such as phospholipases A_2_ (PLA_2_s), hyaluronidases (SVHs), and proteases. More particularly, the great majority of these studies with* Naja* and* Bitis* snakes are part of the work undertaken by Molander et al. [82], aiming to investigate whether plants used in traditional medicine systems would be active against necrosis-inducing enzymes of snake venoms, having tested a total of 226 extracts from 94 plants from the countries of Mali, Democratic Republic of Congo, and South Africa against PLA_2_, SVHs, and proteases from* Bitis arietans* and* Naja nigricollis* (see Tables 2 and 4). Studies evaluating the inhibitory action of medicinal plants against these enzymes are very relevant, since they are involved in several pathological mechanisms produced by snake venoms; however,* in vivo* preclinical assays or, even better, clinical assays are essential for giving even stronger evidences of the effectivity of the use of medicinal plants against snakebites. In this scenario, the study of anti-*Bothrops* plants is more advanced, since quantitatively a higher number of* in vivo* scientific evidences are found in literature. Going the same way, studies with plants inhibiting local tissue damage of* Daboia/Vipera*,* Lachesis*, and* Crotalus* snakes could be also highlighted. However, studies of antiophidic medicinal plants in humans are very scarce: only one clinical study was found in literature, evaluating the inhibitory properties of a polyherbal formulation against local effects from Chinese cobra bite (see Section 4.9).

Hereafter, we describe the main plants with inhibitory potential against local tissue damage induced by snake venoms. It is important to emphasize that the focus of this review is plants against local tissue damage, mainly due to severity of these effects (which could cause permanent disabilities in victims) and the poor effectiveness of available antivenoms against them. So, studies with plants against systemic effects induced by snake were not considered; in addition some plants herein described possess inhibitory action upon systemic effects, although not stated here. For example, the vegetal species* Jatropha gossypiifolia* (Euphorbiaceae), a medicinal plant studied very much by our research group, had showed significative inhibitory action upon hemostatic disorders induced by* B. jararaca* snake venom [96]. So, the antiophidic potential of this species (as well as some others) lies beyond the capacity of inhibit local tissue damage provoked by* B. jararaca* venom, although not described in this review.

In addition, it is important to analyze critically some works dealing with antiophidic activity of plant extracts, since some of them have limitations that could reduce, at least partially, the potentiality of these species. The major limitation is that various studies, especially the early ones, make the evaluation of the plants using a preincubation approach, which consists in the previous inactivation of venom by preincubating it with different proportions of the tested extracts. Although scientifically valid and even recommended by WHO for assessing antiophidic antivenoms [97], this preincubation approach makes a scenario unlikely to be possible in the field, where the medicine would be delivered after the snakebite. In fact, a recent study evaluated the inhibitory action of the medicinal plant* Bellucia dichotoma* (Melastomataceae) against* Bothrops atrox* snake venom using different protocols: preincubation, pretreatment, and posttreatment [98]. The authors observed that while the extract was greatly active when preincubated, this inhibitory activity was drastically reduced or even lost when the extract was injected independently of venom, simulating traditional use. The authors observed that the extract has great amounts of tannins, which are compounds known to precipitate proteins. So, it was concluded that the “pseudo-inhibition” observed after preincubation may be due to the presence of these compounds, suggesting that the preincubation protocol overestimates inhibitory potential of medicinal plants, and for this reason, this kind of approach must be analyzed with caution for estimation of inhibitory potential of medicinal plants [13, 98]. In this sense, many recent studies have been done using protocols of pre- and/or posttreatment, to ensure the potentiality of antiophidic plants, and for most of them, positive results have been found [96, 98–102]. For this reason, studies using preincubation protocol are marked in the tables, for a critical analysis.

Also, it is interesting to note that several of the plants with inhibitory potential against snake venom local toxicities also present other relevant pharmacological activities. This is interesting since it is often discussed in the literature that several antiophidic plants did not neutralize snake venoms per se, but could have antiophidic use once they could relieve some of the symptoms of snake envenoming, especially the local effects. It is related that the presence of tranquilizing, antioxidant, immunostimulating, and/or anti-inflammatory activities in certain plants could be of great interest in the alleviation of snake envenoming symptoms [103, 104]. For example, some studies have shown that anti-inflammatory drugs could inhibit the edematogenic and other snake venom effects related to inflammation, such as necrosis and myotoxicity, induced by* Bothrops* venoms [105, 106]. In fact, many medicinal plants with antiophidic activity also possess significant anti-inflammatory activity* in vivo* [83, 96, 107–110]. Following the same reasoning, some plants with antioxidant activity also possess significant antiophidic effects [95, 96, 104, 111]. In fact, some authors suggest that molecules with antioxidant and/or anti-inflammatory effects could be interesting along with antivenom therapy, helping to reduce the occurrence of secondary/long term complication due to snakebites [112].

Bacterial infection secondary to snakebites is a common complication in envenomed victims [113, 114]. The main source of bacteria is the oral cavity of snakes, but the microbiota in the different layers of the victim's skin or even microorganisms from victim's clothes could also contribute [115, 116]. Abscess formation is a common complication found in patients bitten by Viperidae snakes, being a risk factor for amputation in these patients, and it may be associated with sepsis [113, 114, 117]. A large number of bacteria, including anaerobic species, aerobic gram-negative rods, and a small proportion of gram-positive cocci could be inoculated with snakebites and have been isolated from the abscesses of bitten patients [113, 114]. Microorganisms such as* Staphylococcus*,* Pseudomonas*,* Salmonella*,* Escherichia*,* Providencia*,* Proteus*,* Enterococcus,* and* Bacillus* were already identified in oral cavity of certain snakes [116]. The use of antibiotics following snakebites is often recommended, usually therapeutically than prophylactically, mainly to avoid complications due to infections [114, 118]. In this context, medicinal plants presenting antimicrobial activities, especially against those microorganisms usually detected in snakebite victims' abscesses, could be interesting [115].

Medicinal plants having antimicrobial activities in association with some of the pharmacological properties discussed above (such as anti-inflammatory and antioxidant, e.g.) could be of great value to relieve especially local effects induced by snake venom. In another point of view, it is possible that several related plants in folk medicine as antiophidic agents do not act directly upon venom toxins but indirectly on its symptoms. Anyway, some studies have shown the potentiality of some vegetal species acting in two ways: directly, neutralizing venom toxins, or indirectly, by having some of the pharmacological activities mentioned above. For example,* Jatropha gossypiifolia* (Euphorbiaceae), a plant species studied very much in our research group, showed significant antiophidic properties, inhibiting biological and enzymatic activities from* Bothrops* venoms [96, 119], and presented anti-inflammatory, antioxidant, anticoagulant, and antimicrobial properties in preclinical assays [81]. So, plants which possess these biological activities determined in previous studies might be preferred or prioritized in studies searching for antiophidic plants.

The mechanism by which medicinal plants neutralize the toxic venom constituents is still unknown, but many hypotheses have been proposed, such as protein precipitation, enzyme inactivation, proteolytic degradation, metal chelation, antioxidant action, and a combination of these mechanisms [15]. In this context, some improvements in this understanding have been achieved in the last years, through the use of* in silico* methods (e.g., docking simulations) to analyze the interaction of compounds isolated from plants and certain classes of snake venom toxins such as PLA_2_ and SVMP [120–122].

The use of medicinal plants may present several advantages, such as low cost, being easily available, being stable at room temperature, and possibility of neutralization of a wide range of venom components [15]. In addition, since medicinal plants are an extremely complex mixture, it is possible that there may be a synergistic action of different compounds in plant, acting in distinct targets, inhibiting a broad spectrum of venom toxins [12, 15]. According to literature, interestingly, there are some plants in which the crude extract is more active than the isolated constituents [15], which supports the hypothesis of the synergistic action of plant components.

### 4.2. Plants Inhibiting Naja Snakes

A summary of active plants against* Naja* snakes local effects is presented in Table 2.* Naja* species are commonly called cobras. They typically occur in regions throughout Africa and Southern Asia. The outcomes of venom toxicity include nephro-, neuro-, and cardiotoxicity, respiratory and circulatory collapse, necrosis, hemorrhage, and edema [13]. A great number of the plants showed in this review were tested against* Naja* species. However, it is important to mention that only a very small number of these plants were assessed* in vivo*, and so the scientific evidences of antiophidic activities of these species are based on enzymatic* in vitro* assays, especially against SVHs, a class of toxin particularly relevant in cobras. The study of Molander et al. [82] presented several medicinal plants identified as potent inhibitors of* N. nigricollis* SVHs, PLA_2_, and proteases, which could indicate a potential rich source of inhibitors of necrosis induced by these venom, which must be evaluated* in vivo* later [82]. The same group, in a more recent study [123], investigated the skin permeation,* ex vivo* inhibition of venom induced tissue destruction, and wound healing potential of African plants used against snakebite, which included the most potent inhibitors identified in the previous work [82]. A total of 30 plant species were tested against* Naja nigricollis* and* Bitis arietans* employing* in vitro* and* ex vivo* models [123]. However, although plant extracts have showed potential in inhibiting snake venom enzymes, this study showed no effect against cell death and tissue damage.

### 4.3. Plants Inhibiting Bothrops Snakes

A summary of active plants against* Bothrops* snakes local effects is presented in Table 3. More than 90% of the snakebites reported every year in Latin America are caused by* Bothrops* species [8]. Envenomation by* Bothrops* snakes is characterized by a prominent and complex series of local pathological alterations, which appear rapidly after the bite in the anatomical site where venom is inoculated [168]. In a number of* Bothrops* bite cases, lack of neutralization of local effects results in permanent sequelae, with significative tissue loss [8]. So, the use of a therapeutic approach with high inhibitory potential and easy access and disponibility to victims, which could neutralize rapidly the onset of these local manifestations, is interesting. Most of the inhibitory studies with* Bothrops* snakes were performed in Brazil, which could be associated with richness of Brazilian flora as well as the epidemiological aspects of this country. The work performed by De Moura et al. [33] could be highlighted, where these authors performed an ethnopharmacological-guided screening of plants with reputation against snakebite in Santarém, Western Pará, Brazil. Twelve species were evaluated against* Bothrops jararaca* snake venom induced hemorrhage and some of them presented very significative results, showing, thus, the relevance of traditional knowledge in the survey of antiophidic plants [33].

### 4.4. Plants Inhibiting Bitis Snakes

A summary of active plants against* Bitis* snakes local effects is presented in Table 4. Snakes belonging to the genus* Bitis* are implicated in many accidents with humans in Africa. The envenomation by* Bitis* often results in severe local damage, hypotension, coagulopathy, thrombocytopenia, and spontaneous local bleeding and, in the absence of antivenom therapy, the accident can be fatal.* Bitis arietans* is one of the three species of snakes of medical importance in Africa and its venom is considered the most toxic venom of the viper group [169]. Regarding the plants with inhibitory action upon* Bitis* snakes, only one* in vivo* study of antiophidic activity was found until date. Although many works have been showing the potential of medicinal plants against several snake venoms, only three works were identified evaluating the action of plants against* Bitis*, from which two are the same screening studies of plants against* Naja* snake venom discussed before (Section 4.2) [82, 123].

### 4.5. Plants Inhibiting Daboia/Vipera Snakes

A summary of active plants against* Daboia/Vipera* snakes local effects is presented in Table 5. The* Daboia* genus is represented by a single species, named* Daboia russelii*, also popularly known as Russell's viper. This species is widespread in many parts of Asia and is responsible for large morbimortality due to snakebites in this continent [183, 184]. Russell's viper was formerly classified in* Vipera* genus and is therefore better known as* Vipera russelii*, since the new accepted nomenclature* (Daboia russelii)* is not yet universally followed [184]. For this reason, to avoid confounding, we use the term* Daboia/Vipera* in some occasions.

In humans, Russell's viper bite causes severe local tissue damage; more frequently the necrosis results in an irreversible loss of tissue and requires amputation of the affected limb [182, 183, 185]. As observed with* Bothrops* snakes, several studies have showed the inhibitory potential of medicinal plants against local effects of Russell's viper venom, including several preclinical* in vivo* studies.

### 4.6. Plants Inhibiting Lachesis Snakes

A summary of active plants against* Lachesis* snakes local effects is presented in Table 6.* Lachesis muta* is the longest venomous snake in the Americas and is distributed in the equatorial forests east of the Andes, ranging from eastern Ecuador, Colombia, Peru, northern Bolivia, and eastern and northern Venezuela, to Guyana, French Guyana, Surinam, and northern Brazil [100, 186].* L. muta* snakebites are mainly characterized by systemic (generalized bleeding, coagulopathy, renal failure, and shock) and local effects (pain, hemorrhage, edema, and necrosis). In South America,* Bothrops* species has a higher incidence of accidents than* L. muta*, but, on the other hand,* Lachesis* bites led to more severe symptoms and have lethality indexes significantly higher than* Bothrops* [100, 186, 187]. Thus, the study of medicinal plants against these snakes, too, is of very much relevance. However, only a few studies were detected with plants against* Lachesis* snakes.

### 4.7. Plants Inhibiting Crotalus Snakes

A summary of active plants against* Crotalus* snakes local effects is presented in Table 7. Snakes from* Crotalus durissus* complex, popularly known as rattlesnakes, are dispersed northward into North America and southward into South America. Species of the* Crotalus durissus* complex pose a serious medical problem in many parts of the America [199]. Crotalic venom is considered highly toxic and more lethal in comparison with that of the genus* Bothrops*, having three main actions: neurotoxic, myotoxic, and coagulant [200, 201]. The crotalic accident is characterized by local and systemic manifestations, but while the local alterations are only discrete, the systemic manifestations are severe, leading to high chances of death [201]. Probably due to this low local effect in envenomed victims, the inhibition of these effects by plants is, until now, little investigated, especially when compared to other species with characteristic severe local effects.

### 4.8. Plants Inhibiting Other Snakes

Besides the snakes discussed above, some other studies are found with plants inhibiting other snake species, such as those from* Echis* and* Bungarus* genus. For other snakes species such as* Calloselasma rhodostoma*,* Philodryas olfersii,* and* Montivipera xanthina*, only isolated studies with a single plant, in each one, were found. These plants are summarized in Table 8. Many reasons may be stated for this lack of studies, such as low level of local effects, incidence restricted to a small region of the world, and usual low efficacy of plant extracts due to possible extremely high toxicity. However, it is important to highlight that the lack of studies does not mean a lower medical relevance of these species. For example, the saw-scaled viper* (Echis carinatus)* and the common Indian krait* (Bungarus caeruleus)*, along with spectacled cobra* (Naja naja)* and Russell's viper* (Daboia russelii)*, are included among the referred “Big Four” venomous snakes of India, being responsible for the majority of morbid complications, characterized by persistent and progressive tissue necrosis even after treatment with antivenom [195, 202]. Therefore, future studies with plants aiming at the inhibition of the local effects induced by these snakes are encouraged.

### 4.9. Studies in Humans

Along our antiophidic plants database, only one clinical study was found in literature, evaluating the inhibitory properties of a polyherbal formulation, externally applied, against soft-tissue necrosis after* Naja atra* (Chinese cobra) bite [203]. This polyherbal formulation, known in China as Jidesheng antivenom, is composed of the following ingredients: Ganchan* (Succys Bufo)*, Dijincao* (Herba Euphorbiae Humifusae)*, Chonglou* (Rhizoma Paridis Chonglou)*, and Wugong* (Scolopendra)*. This was a retrospective study performed with 126 patients with skin and soft-tissue necrosis due cobra bite, with the control group being treated externally with 40% glyceride magnesium sulfate (*n* = 52) and the treatment group performed by application of Jidesheng antivenom externally (*n* = 74). The authors observed statistically significant differences in maximum local necrotic area of skin and soft tissues, healing time, and skin-grafting rate between the control and treatment groups (*P* < 0.05), thus indicating that external application of Jidesheng antivenom may help to promote wound healing and reduce the skin-grafting rate in cases of skin and soft-tissue necrosis due to Chinese cobra bite [203]. Considering the composition of the Jidesheng antivenom, the authors discuss that each ingredient in this product may exert antipyretic, antidotal, antiphlogistic, and analgesic effects, according to previous results with each ingredient isolated, which could contribute to the inhibitory effect observed by the formulation [203]. The result obtained in this clinical study is very promising, since it shows that a plant-derived product showed significant results in humans, thus pointing to the potentiality of this kind of product in treatment of snake venom induced local effects. However, only one study is insufficient to ensure the potentiality of medicinal plants against snakebites, with performing more clinical studies, preferentially controlled and randomized ones, to bring more evidences of the viability of the approach for future safe and effective use in humans being necessary. So, more clinical studies, especially ones with those plants highlighted in this review and those presenting good preclinical* in vivo* evidences of antiophidic efficacy, are highly encouraged.

## 5. Concluding Remarks

The popular use of vegetal species does not necessarily imply efficacy, but it gives a selected list of medicinal plants that can be primarily studied in pharmacologic assays for possible antiophidic effects, directing future studies in this area. In fact, a great number of these species that have been evaluated against local tissue damage induced by several snake species showed inhibitory potential against hyaluronidase, phospholipase, proteolytic, hemorrhagic, myotoxic, and edematogenic activities, among others. Therefore, considering the limitations of conventional antivenom serotherapy, especially its poor efficacy against local effects, the treatment with medicinal plants may provide a potential adjuvant alternative to treat snakebites, being used to complement the activity and effectiveness of available snake venom therapy. The main potential advantages of antiophidic plants are their low cost, easy access, stability at room temperature, and ability to neutralize a broad spectrum of toxins, including the local tissue damage.

Interestingly, some studies have showed that the crude extracts are more powerful than the individual herbal compounds, which could, at a certain extent, justify the development of herbal products containing these plants instead of medicines containing isolated compounds, which in turn could be more rapidly available in market, after proof of safety, effectiveness, and quality of these products. However, despite the existence of many plants with great potential, no natural antiophidic product is available in market, which points to question of the need for further studies. Only a few numbers of patents regarding herbal products against snakebites were found in literature. Some patents regarding the use of Chinese medicinal plants against snake and bug bites were found. In our research group, two patents were deposited concerning the processes of obtaining extracts, fraction, isolated compounds, and pharmaceutical compositions of some plants studied by our group applied in the treatment of accidents with venomous animals (BR 10 2013 034046 4 A2 and BR 10 2012 026958 9 A2). Thus, the number of patents with antiophidic herbal products is still relatively small. For this reason, we encourage pharmacologists and toxinologists around the world to intensify studies with antiophidic plants, especially prioritizing those with the greatest number of indications in traditional medicine and emphasizing clinical studies with the most active plants in preclinical studies, given that the low number of human studies is one of the major obstacles for the future application of herbal products with antiophidic potential. No less important, toxicological studies are also extremely necessary to ensure the safety of these products.

In conclusion, the data presented in this review provides an updated scenario for and insights into future research aiming at validation of medicinal plants as antiophidic agents and, based on scientific evidences, strengthens the potentiality of medicinal plants and ethnopharmacological knowledge as a tool for design of potent inhibitors and/or herbal medicines against venom toxins.

## Figures and Tables

**Table 1:** List of medicinal plants used against snakebites.

Plant name	Countries	Parts used	Use	Reference(s)
Acanthaceae				
*Acanthus arboreus*	Sri Lanka	ND	I	[18]
*Andrographis echioides* (syn. *Indoneesiella echioides*)	India	Shoot	ND	[19]
*Andrographis lineata*	India	Flower, leaf	I	[20]
*Andrographis paniculata*	India	Leaf, whole plant	I, E	[16, 20–26]
*Barleria cristata*	India, Pakistan	Leaf, root, seed, whole plant	E	[17, 19, 25]
*Barleria lupulina*	Sri Lanka	ND	I	[18]
*Blechum pyramidatum*	Nicaragua	Leaf, whole plant	I	[27]
*Blepharis maderaspatensis*	India	Leaf	I	[28]
*Clinacanthus nutans*	India	Leaf	E	[20]
*Dicliptera paniculata *(syn.* Peristrophe paniculata*)	India	Root, whole plant	I, E	[24, 25]
*Fittonia albivenis*	Peru	Aerial parts	E	[29]
*Hygrophila auriculata*	India, Sri Lanka	Seed	I	[18, 23]
*Justicia adhatoda *(syn.* Adhatoda vasica*)	India, Pakistan, Sri Lanka	Flower, leaf, root	I, E	[16–18, 30]
*Justicia calyculata*	Kenya	Aerial parts	E	[31]
*Justicia gendarussa*	Bangladesh	Leaf	I	[30, 32]
*Justicia japonica *(syn*. Justicia simplex*)	India	Leaf	I	[23]
*Justicia pectoralis* ^*∗*^	Brazil	Leaf	I	[33]
*Justicia procumbens*	Sri Lanka	ND	E	[18]
*Justicia secunda* ^#^	Colombia	Flower, leaf, root, whole plant	I, E	[34, 35]
*Rhinacanthus nasutus*	India	Leaf, root	I, E	[16, 28]
*Thunbergia alata*	Colombia	Flower, leaf	E	[34]
*Trichanthera gigantea*	Colombia	Leaf, root	E	[34]
Acoraceae				
*Acorus calamus*	Bangladesh, India, Pakistan, Sri Lanka	Rhizome, root	I, E	[17, 18, 20, 22, 25, 32, 36]
Adoxaceae				
*Sambucus nigra*	Spain	Flower	E	[37]
Amaranthaceae				
*Achyranthes aspera *(syn.* Achyranthes porphyristachya*)^#^	Bangladesh, Colombia, India	Fruit, inflorescence, leaf, root, seed, stem, whole plant	I, E	[17, 20, 22, 23, 25, 28, 30, 32, 35, 38–41]
*Aerva lanata*	India, Sri Lanka	Rhizome	I	[18, 20]
*Aerva sanguinolenta*	Bangladesh	Leaf	ND	[30]
*Alternanthera albotomentosa*	Colombia	Leaf	E	[34]
*Alternanthera brasiliana*	Brazil	Flower	I	[33]
*Alternanthera sessilis*	Sri Lanka	ND	I	[18]
*Amaranthus blitum*	India	Root	I	[25]
*Amaranthus dubius*	Colombia	Fruit peel, leaf, root, seed	E	[34]
*Amaranthus polygonoides*	Sri Lanka	ND	E	[18]
*Amaranthus spinosus*	India	Leaf, root, stem, whole plant	E	[17, 19, 32, 42]
*Amaranthus viridis*	Bangladesh, India, Pakistan, Sri Lanka	Leaf, stem, whole plant	E	[17, 18, 30, 42, 43]
*Chenopodium album*	Bangladesh, India, Pakistan	Fruit, root, whole plant	E	[17, 32, 41]
*Cyathula tomentosa*	India	Leaf	ND	[19]
*Dysphania ambrosioides *(syn.* Chenopodium ambrosioides*)	Colombia	Whole plant	E	[34]
Amaryllidaceae				
*Allium ascalonicum*	Sri Lanka	ND	I	[18]
*Allium cepa* ^*∗*^	Bangladesh, Colombia, India, Kenya	Bulb, latex, leaf	E	[20, 25, 31, 32, 34, 40]
*Allium sativum* ^*∗*^	Colombia, India, Sri Lanka, Spain	Bulb, inflorescence, leaf	I, E	[18, 22, 23, 37, 44]
*Ammocharis tinneana*	Kenya	Latex	ND	[31]
*Crinum asiaticum*	Sri Lanka	ND	E	[18]
*Crinum latifolium*	Sri Lanka	ND	E	[18]
*Hymenocallis littoralis*	Nicaragua	Leaf, root	I, E	[27]
Anacardiaceae				
*Anacardium occidentale* ^*∗*^	India, Nicaragua	Bark, fruit, leaf, root	I, E	[27, 45]
*Buchanania cochinchinensis *(syn.* Buchanania lanzan*)	India	Bark	E	[24, 38]
*Mangifera indica* ^*∗*^	Bangladesh, Pakistan, Sri Lanka	Leaf	E	[17, 18, 32]
*Mangifera minor*	Papua New Guinea	Bark	I	[46]
*Pistacia chinensis*	Pakistan	Gall	E	[17]
*Pistacia chinensis *subsp. *integerrima*^*∗*^	India, Pakistan	Gall, leaf	E	[17, 19]
*Semecarpus anacardium*	India	Root	I	[20]
*Semecarpus coriacea*	Sri Lanka	ND	E	[18]
*Spondias dulcis*	Sri Lanka	ND	E	[18]
*Spondias mombin* ^*∗*^	Peru	Bark	ND	[29]
*Tapirira guianensis*	Colombia	Oil	E	[34]
Annonaceae				
*Annona montana* ^#^	Brazil	Leaf	I	[33, 47]
*Annona muricata*	Brazil	Leaf	ND	[48]
*Annona senegalensis* ^*∗*^	Kenya	Leaf	I, E	[31]
*Annona squamosa*	Bangladesh, India	Bark, fruit	I, E	[23, 32]
*Polyalthia longifolia*	Bangladesh	Whole plant	ND	[30]
*Uvaria scheffleri*	Kenya	Leaf, root	E	[31]
Apiaceae				
*Centella asiatica*	Sri Lanka	ND	E	[18]
*Conium maculatum*	Spain	Leaf	E	[37]
*Coriandrum sativum*	Sri Lanka	ND	I	[18]
*Eryngium bourgatii*	Spain	Aerial parts, root	E	[37]
*Eryngium campestre*	Spain	Aerial parts, root	E	[37]
*Eryngium foetidum*	Nicaragua, Sri Lanka	Leaf	I, E	[18, 27]
*Steganotaenia araliacea*	Kenya	Root	E	[31]
*Trachyspermum ammi*	Sri Lanka	ND	I	[18]
*Trachyspermum roxburghianum*	Sri Lanka	ND	I	[18]
Apocynaceae				
*Allamanda cathartica* ^*∗*^	Colombia	Aerial parts, branch, leaf, stem	I, E	[35, 44]
*Alstonia scholaris*	Bangladesh, India, Sri Lanka	Bark, flower, latex, leaf, root	I, E	[18, 19, 32]
*Alstonia venenata*	Sri Lanka	ND	E	[18]
*Asclepias curassavica*	Nicaragua	Bark, flower, latex, leaf, root, whole plant	I, E	[27]
*Blepharodon mucronatum*	Nicaragua	Leaf, whole plant	I, E	[27]
*Calotropis acia*	Sri Lanka	ND	I, E	[18]
*Calotropis gigantea*	Bangladesh, India, Sri Lanka	Latex, leaf, root	I, E	[16, 18, 20, 22, 23, 28, 32, 38, 49]
*Calotropis procera* ^*∗*^	Bangladesh, India, Pakistan	Flower, latex, leaf, root, shoot	I, E	[17, 25, 32, 38, 40, 41]
*Cascabela thevetia *(syn.* Thevetia peruviana*)	Brazil	Bark, seed	E	[50]
*Catharanthus roseus*	Bangladesh, Colombia	Flower, leaf	I, E	[32, 34]
*Cerbera floribunda*	Papua New Guinea	Latex	E	[46]
*Cerbera odollam*	Sri Lanka	ND	E	[18]
*Cryptolepis dubia *(syn.* Cryptolepis buchanani*)	India, Sri Lanka	Root	ND	[18, 19]
*Cynanchum viminale *(syn.* Sarcostemma viminale*)	India	Whole plant	E	[38]
*Dregea volubilis *(syn.* Wattakaka volubilis*)	India, Sri Lanka	Root	I, E	[18, 23]
*Echidnopsis dammanniana*	Ethiopia	Stem	E	[51]
*Echites umbellatus*	Nicaragua	Root	I	[27]
*Gymnema sylvestre*	India	Leaf, root	I, E	[19, 20, 22, 23, 28, 52]
*Hemidesmus indicus* ^*∗*^	Bangladesh, India, Sri Lanka	Root, whole plant	I, E	[18, 20, 22, 25, 28, 32, 38]
*Holarrhena pubescens *(syn.* Holarrhena antidysenterica*)	Bangladesh, India	Bark, root, stem	I, E	[24, 32, 38]
*Hoya ovalifolia*	Sri Lanka	ND	I	[18]
*Hunteria zeylanica*	Sri Lanka	ND	E	[18]
*Ichnocarpus frutescens*	Bangladesh	Root	I	[32]
*Nerium oleander *(syn.* Nerium indicum*)^#^	India, Pakistan, Sri Lanka	Leaf, root, seed	E	[17, 18, 20, 28, 41, 53]
*Odontadenia puncticulosa*	Nicaragua	Leaf	I	[27]
*Pergularia daemia*	India, Namibia	Leaf	I	[19, 28, 54]
*Rauvolfia serpentina*	Bangladesh, India, Sri Lanka	Flower, leaf, rhizome, root, seed	I, E	[18, 20, 22, 28, 30, 32]
*Rauvolfia tetraphylla *(syn.* Rauvolfia canescens*)^#^	Bangladesh, India	Root	E	[16, 30]
*Tabernaemontana dichotoma*	Sri Lanka	ND	E	[18]
*Tabernaemontana divaricata*	Sri Lanka	ND	I	[18]
*Tabernaemontana sananho*	Peru	Leaf	E	[29]
*Tylophora indica* ^*∗*^	Bangladesh, India	Leaf	I	[23, 30, 32]
*Tylophora longifolia*	India	Flower, leaf	ND	[20]
*Vincetoxicum hirundinaria*	India	Root	ND	[19]
*Willughbeia edulis*	Bangladesh	Stem	E	[32]
*Wrightia antidysenterica*	Sri Lanka	ND	E	[18]
*Wrightia arborea*	India	Bark	ND	[19]
*Wrightia tinctoria*	India	Leaf	ND	[38]
Aponogetonaceae				
*Aponogeton crispus*	Sri Lanka	ND	E	[18]
Araceae				
*Alocasia cucullata* ^#^	Colombia	Rhizome, root	E	[35, 44]
*Amorphophallus commutatus*	India	Tuber	ND	[55]
*Amorphophallus paeoniifolius*	Sri Lanka	ND	I	[18]
*Anaphyllum beddomei*	India	Rhizome	E	[16]
*Anthurium marmoratum*	Colombia	Branch, leaf, stem	E	[35]
*Arisaema concinnum*	India	Fruit, tuber	ND	[19]
*Arisaema flavum*	Pakistan	Rhizome	ND	[17]
*Arisaema jacquemontii*	India, Pakistan	Flower, leaf, tuber	ND	[17, 19, 56]
*Arisaema tortuosum*	India	Bulb, tuber	I	[38, 55]
*Caladium bicolor*	Peru	Tuber	E	[57]
*Dieffenbachia longispatha* ^#^	Colombia	Whole plant	I, E	[35]
*Dieffenbachia parlatorei*	Colombia	Root	E	[44]
*Dracontium croatii* ^*∗*^	Colombia	Rhizome	I, E	[35]
*Dracontium spruceanum*	Colombia, Peru	Stem, tuber, root	E	[29, 34, 44, 57]
*Dracunculus vulgaris*	Spain	Bulb, flower	E	[37]
*Homalomena aromatica*	Bangladesh	Rhizome	E	[32]
*Homalomena peltata*	Colombia	Leaf	E	[44]
*Homalomena picturata*	Colombia	Leaf	E	[34]
*Lasia spinosa*	Sri Lanka	ND	E	[18]
*Philodendron deltoideum*	Peru	Aerial parts	I, E	[29]
*Philodendron hederaceum*	Nicaragua	Leaf, stem	I, E	[27]
*Philodendron heleniae*	Colombia	Leaf	E	[44]
*Philodendron megalophyllum* ^*∗*^	Brazil	Vine	I	[33]
*Philodendron tripartitum* ^*∗*^	Colombia	Branch, leaf	E	[35]
*Pothos scandens*	Sri Lanka	ND	I	[18]
*Rhodospatha oblongata*	Colombia	Rhizome	E	[35]
*Sauromatum venosum*	India, Pakistan	Leaf, tuber	I, E	[17, 38]
*Typhonium roxburghii*	Sri Lanka	ND	I	[18]
*Xanthosoma poeppigii*	Peru	Stem	E	[57]
Araliaceae				
*Osmoxylon micranthum*	Papua New Guinea	Latex	E	[46]
Arecaceae				
*Areca catechu*	Sri Lanka	ND	E	[18]
*Caryota urens*	Sri Lanka	ND	I	[18]
*Cocos nucifera*	Sri Lanka	ND	I	[18]
*Corypha umbraculifera*	Sri Lanka	ND	E	[18]
*Euterpe edulis*	Brazil	Latex	E	[50]
*Euterpe oleracea*	Brazil	Fruit	E	[33]
*Phoenix pusilla*	Sri Lanka	ND	I	[18]
*Syagrus coronata*	Brazil	Bark	ND	[47]
Aristolochiaceae				
*Aristolochia birostris*	Brazil	Whole plant	I	[47]
*Aristolochia bracteolata* ^*∗*^	India, Sri Lanka	Fruit, leaf, whole plant	I, E	[18, 23, 38, 55]
*Aristolochia clematitis*	Serbia	Rhizome	ND	[58]
*Aristolochia cordiflora*	Colombia	Leaf, stem	I, E	[34, 44]
*Aristolochia grandiflora* ^#^	Colombia	Whole plant	I, E	[35]
*Aristolochia indica* ^*∗*^	Bangladesh, India	Leaf, root, whole plant	I, E	[16, 20, 22, 23, 28, 30, 32]
*Aristolochia ovalifolia*	Mexico	Root	ND	[59]
*Aristolochia pilosa* ^#^	Colombia	Root	I, E	[35]
*Aristolochia tagala*	India	Whole plant	I, E	[16]
*Aristolochia trilobata*	Brazil, Nicaragua	Leaf, root, whole plant	I, E	[27, 50]
*Thottea siliquosa*	India	Leaf, root	E	[16, 26]
Asparagaceae				
*Asparagus racemosus*	Bangladesh, Sri Lanka	Leaf, root	E	[18, 30, 32]
*Drimia indica *(syn.* Urginea indica*)	India	Bulb	E	[25]
*Peliosanthes teta*	Bangladesh	Root	E	[32]
*Sansevieria parva*	Kenya	Latex	E	[31]
*Sansevieria roxburghiana*	India	Rhizome	I	[23]
*Sansevieria trifasciata*	Bangladesh, Colombia	Aerial parts, whole plant	E	[30, 34, 60]
*Sansevieria zeylanica*	Sri Lanka	ND	E	[18]
Aspleniaceae				
*Asplenium dalhousiae *(syn.* Ceterach dalhousiae*)	Pakistan	Leaf	ND	[17]
Asteraceae				
*Achillea millefolium*	India	Whole plant	I	[20]
*Acmella paniculata *(syn. *Spilanthes paniculata*)	Sri Lanka	ND	I	[18]
*Adenostemma fosbergii*	Ecuador	Leaf	I	[61]
*Adenostemma lavenia* ^#^	Colombia	Whole plant	E	[35]
*Ageratum conyzoides* ^#^	Colombia, India, Bangladesh	Flower, leaf, root	E	[19, 24, 32, 34]
*Ageratum houstonianum*	Pakistan	Inflorescence, leaf	E	[17]
*Ambrosia peruviana *(syn.* Ambrosia cumanensis*)	Colombia	Aerial parts, whole plant	I, E	[34, 44]
*Artemisia maritima*	Pakistan	Whole plant	E	[17]
*Artemisia scoparia*	India, Pakistan	Whole plant	E	[17, 40]
*Austroeupatorium inulifolium*	Colombia	Leaf	E	[34, 44]
*Ayapana triplinervis *(syn.* Eupatorium ayapana*,* Eupatorium triplinerve*)	Brazil	Leaf	I	[33, 50]
*Baccharis inamoena *(syn. *Baccharis trinervis*)	Colombia	Aerial parts, whole plant	E	[34]
*Baccharoides anthelmintica *(syn.* Centratherum anthelminticum*)	India	Seed	ND	[26]
*Bidens biternata*	India	Leaf	E	[25]
*Bidens pilosa*	Kenya	Leaf	E	[31]
*Blumea axillaris*	Sri Lanka	ND	I	[18]
*Blumea brevipes *(syn.* Laggera brevipes*)	Kenya	Root	ND	[31]
*Calendula officinalis*	India	Flower	I	[20, 28]
*Chromolaena odorata*	Colombia	Whole plant	E	[34]
*Clibadium sylvestre* ^#^	Colombia	Whole plant	I, E	[35]
*Conyza sumatrensis*	Kenya	Leaf	I	[31]
*Cyanthillium cinereum*	Sri Lanka	ND	E	[18]
*Eclipta prostrata *(syn. *Eclipta alba*)^*∗*^	Bangladesh, India, Pakistan, Sri Lanka	Leaf, whole plant	I, E	[17, 18, 20, 28, 30, 40]
*Elephantopus scaber*	Sri Lanka	ND	E	[18]
*Emilia sonchifolia*	Bangladesh, Colombia, India, Sri Lanka	Leaf, whole plant	I, E	[16, 18, 30, 34]
*Erechtites valerianifolia* ^#^	Colombia	Branch, leaf, stem	I, E	[35]
*Gnaphalium purpureum*	Sri Lanka	ND	I, E	[18]
*Gynura hispida*	Sri Lanka	ND	I	[18]
*Helianthus annuus*	India	Seed	E	[20]
*Inula helenium*	Serbia	Root	E	[58]
*Laggera alata*	Sri Lanka	ND	E	[18]
*Linzia glabra* (syn. *Vernonia glabra*)	Kenya	Leaf	E	[31]
*Microglossa pyrifolia*	Kenya	Leaf	E	[31]
*Mikania cordata*	Bangladesh	Leaf	E	[32]
*Mikania cordifolia*	Nicaragua	Leaf, stem, whole plant	I, E	[27]
*Mikania guaco* ^#^	Colombia, Nicaragua	Leaf, stem, whole plant	I, E	[27, 35, 44]
*Neurolaena lobata* ^*∗*^	Colombia, Nicaragua	Aerial parts, branch, leaf, stem	I, E	[27, 35, 44]
*Pentanema indicum*	India, Sri Lanka	Leaf, root	I	[18, 23]
*Pluchea indica* ^*∗*^	India	Flower, seed	I, E	[20]
*Pseudelephantopus spicatus* ^*∗*^	Colombia	Whole plant	E	[44]
*Saussurea simpsoniana*	India	Flower	ND	[19]
*Senecio chrysanthemoides*	Pakistan	Whole plant	E	[17]
*Seriphidium brevifolium *(syn.* Artemisia brevifolia*)	Pakistan	Flower, leaf	E	[17]
*Solanecio mannii*	Kenya	Leaf	E	[31]
*Sphaeranthus africanus*	Sri Lanka	ND	I	[18]
*Sphaeranthus indicus*	Sri Lanka	ND	I	[18]
*Sphagneticola trilobata*	Nicaragua	Flower, leaf, stem, whole plant	I	[27]
*Tagetes minuta*	Kenya	Leaf	E	[31]
*Taraxacum officinale*	Colombia, Pakistan	Leaf, root, whole plant	I, E	[17, 34]
*Tithonia diversifolia*	Colombia, Kenya	Leaf, whole plant	I, E	[31, 34]
*Tricholepis glaberrima*	India	Root	ND	[19]
*Verbesina gigantea*	Colombia	Root, stem	I, E	[34]
*Vernonanthura patens*	Colombia	Whole plant	E	[34]
*Vernonia zeylanicum*	Sri Lanka	ND	I, E	[18]
*Wedelia calendulacea*	India	Leaf	I	[20]
*Wollastonia biflora *(syn.* Wedelia biflora*)	Sri Lanka	ND	E	[18]
*Xanthium strumarium*	Pakistan	Aerial parts	E	[17]
Balsaminaceae				
*Impatiens balsamina*	Colombia	Flower	I, E	[34]
Begoniaceae				
*Begonia annulata *(syn*. Begonia barbata*)	Bangladesh	Leaf, stem	E	[32]
Berberidaceae				
*Dysosma pleiantha*	China, Taiwan	Rhizome	ND	[62]
Betulaceae				
*Betula alnoides*	India	Bark, leaf	ND	[19]
Bignoniaceae				
*Crescentia cujete* ^#^	Colombia	Fruit	I	[35]
*Dolichandra unguis-cati *(syn.* Macfadyena unguis-cati*)^*#*^	Colombia	Whole plant	E	[35]
*Handroanthus barbatus *(syn. *Tabebuia barbata*)	Brazil	Leaf	I	[33]
*Mansoa alliacea*	Peru	Bark, root	I	[57]
*Oroxylum indicum*	Bangladesh, Sri Lanka	Bark	E	[18, 32]
*Stereospermum chelonoides*	Sri Lanka	ND	I	[18]
*Stereospermum colais*	Sri Lanka	ND	E	[18]
*Tabebuia rosea* ^*∗*^	Colombia	Bark	I, E	[35]
Bixaceae				
*Bixa orellana* ^*∗*^	Bangladesh, Colombia, Nicaragua	Branch, fruit, latex, leaf, root, stem	I, E	[27, 32]
*Cochlospermum vitifolium*	Colombia	Aerial parts	E	[34]
Boraginaceae				
*Cordia dichotoma *(syn.* Cordia obliqua*)	Pakistan	Bark, fruit	ND	[17]
*Cordia spinescens *(syn.* Varronia spinescens*)	Colombia	Leaf	E	[34]
*Cynoglossum zeylanicum*	India	Root	I	[63]
*Echium vulgare*	Spain	Aerial parts	ND	[37]
*Ehretia microphylla *(syn. *Ehretia buxifolia*)	India, Sri Lanka	Root	I, E	[18, 20]
*Heliotropium europaeum*	Pakistan	Whole plant	E	[17]
*Heliotropium indicum* ^#^	Nicaragua	Leaf, whole plant	I	[27]
*Tournefortia cuspidata* ^#^	Colombia	Branch, leaf, stem	E	[35]
*Trichodesma indicum* ^*∗*^	Pakistan	Leaf, root	ND	[17]
*Trichodesma zeylanicum*	India	Root	I, E	[20]
Brassicaceae				
*Brassica juncea*	Sri Lanka	ND	E	[18]
*Brassica rapa* (syn. *Brassica campestris*)	India	ND	E	[25]
*Lepidium virginicum*	Colombia	Whole plant	E	[34]
Bromeliaceae				
*Ananas comosus*	Nicaragua, Sri Lanka	Flower, leaf, root	I, E	[18, 27]
*Bromelia pinguin*	Nicaragua	Leaf	I, E	[27]
Burseraceae				
*Boswellia serrata*	India	Bark	I	[24]
*Bursera simaruba*	Nicaragua	Bark, whole plant	I	[27]
*Canarium zeylanicum*	Sri Lanka	ND	E	[18]
Cactaceae				
*Opuntia ficus-indica *(syn.* Opuntia vulgaris*)	India	Root	ND	[25]
*Pereskia bleo* ^#^	Colombia	Leaf, stem	E	[35]
Calophyllaceae				
*Calophyllum inophyllum*	Sri Lanka	ND	E	[18]
*Mesua ferrea*	Sri Lanka	ND	I, E	[18]
Campanulaceae				
*Hippobroma longiflora*	Nicaragua	Leaf, root, whole plant	I, E	[27]
Cannabaceae				
*Cannabis sativa*	India, Sri Lanka	ND	I	[18, 40]
Cannaceae				
*Canna indica*	Sri Lanka	ND	E	[18]
Capparaceae				
*Capparis decidua*	Pakistan	Flower, shoot	E	[17]
*Capparis moonii*	Sri Lanka	ND	I	[18]
*Capparis roxburghii*	Sri Lanka	ND	E	[18]
*Capparis zeylanica*	Sri Lanka	ND	I, E	[18]
*Carica papaya* ^#^	India	Fruit	ND	[41]
*Crateva adansonii*	Sri Lanka	ND	I	[18]
*Crateva tapia *(syn.* Crateva benthamii*)^*#*^	Brazil	Leaf	E	[33]
*Cynophalla flexuosa *(syn.* Capparis flexuosa*)	Brazil	Bark	I	[64]
Caprifoliaceae				
*Nardostachys jatamansi*	India	Root	ND	[19]
*Valeriana jatamansi*	Pakistan, Sri Lanka	Root	I, E	[17, 18]
Celastraceae				
*Cassine glauca*	India, Sri Lanka	Leaf	I	[18, 19]
*Celastrus paniculatus*	India	Bark, root, seed	I	[19, 38]
*Gymnosporia emarginata*	Sri Lanka	ND	I	[18]
*Parnassia nubicola*	India	Tuber	ND	[19]
Chrysobalanaceae				
*Parinari capensis*	Namibia	Root	ND	[65]
Cleomaceae				
*Cleome gynandra*	Sri Lanka	ND	E	[18]
*Cleome viscosa*	Sri Lanka	ND	I	[18]
Clusiaceae				
*Garcinia morella*	Sri Lanka	ND	I, E	[18]
*Garcinia xanthochymus*	Sri Lanka	ND	I, E	[18]
Colchicaceae				
*Gloriosa superba* ^*∗*^	India, Pakistan, Sri Lanka	Tuber	I, E	[17, 18, 20, 28, 38, 40]
Combretaceae				
*Anogeissus latifolia*	Bangladesh, India	Bark, whole plant	I, E	[25, 30, 38]
*Combretum collinum*	Kenya	Root	E	[31]
*Combretum molle* ^*∗*^	Kenya	Bark, root	I	[31]
*Getonia floribunda* (syn. *Calycopteris floribunda*)	Bangladesh	Root	E	[32]
*Terminalia arjuna* ^*∗*^	Bangladesh, India	Bark	I, E	[20, 32]
*Terminalia bellirica*	Sri Lanka	ND	I	[18]
*Terminalia chebula*	Sri Lanka	ND	I	[18]
Commelinaceae				
*Callisia gracilis*	Colombia	Flower, leaf	I, E	[34]
*Commelina benghalensis*	India, Sri Lanka	Root	ND	[18, 42]
Connaraceae				
*Connarus favosus* ^*∗*^	Brazil	Bark	I	[33]
*Connarus monocarpus*	Sri Lanka	ND	E	[18]
Convolvulaceae				
*Argyreia nervosa *(syn.* Argyreia speciosa*)	India	Root, seed	ND	[19]
*Argyreia populifolia*	Sri Lanka	ND	I	[18]
*Cuscuta reflexa*	Sri Lanka	ND	E	[18]
*Dichondra repens*	Kenya	Leaf	E	[31]
*Evolvulus alsinoides*	India, Sri Lanka	Root	I	[18, 23]
*Ipomoea alba*	Sri Lanka	ND	E	[18]
*Ipomoea aquatica*	Bangladesh	Leaf, whole plant	ND	[30]
*Ipomoea asarifolia*	Sri Lanka	ND	I, E	[18]
*Ipomoea cairica* ^#^	Colombia	Branch, leaf, stem	E	[35]
*Ipomoea mauritiana*	Nicaragua	Leaf	I, E	[27]
*Ipomoea pes-caprae*	Nicaragua	Leaf, seed	I	[27]
*Ipomoea pes-tigridis*	India, Sri Lanka	Root	I, E	[18, 19, 24, 39]
*Ipomoea setifera*	Nicaragua	Leaf	I, E	[27]
*Ipomoea triloba*	Sri Lanka	ND	I	[18]
*Operculina pteripes*	Nicaragua	Leaf	E	[27]
*Rivea hypocrateriformis*	India	ND	I	[24]
Cornaceae				
*Alangium salviifolium*	India	Bark	I	[20, 23]
Costaceae				
*Cheilocostus speciosus* (syn. *Costus speciosus*)	Bangladesh, India, Sri Lanka	Bulb, leaf, stem, root, tuber	I, E	[18, 19, 32, 55]
*Costus guanaiensis* ^#^	Colombia	Stem	I, E	[35]
*Costus lasius* ^*∗*^	Colombia	Branch, leaf, stem	I, E	[35]
*Costus lima*	Colombia	Stem	E	[34]
Crassulaceae				
*Bryophyllum pinnatum *(syn.* Kalanchoe pinnata*)^*∗*^	India	Leaf	ND	[22, 42]
*Kalanchoe laciniata* (syn. *Kalanchoe brasiliensis*)^*∗*^	Brazil	Leaf	E	[33]
Cucurbitaceae				
*Benincasa hispida*	Sri Lanka	ND	E	[18]
*Citrullus colocynthis* ^*∗*^	India, Pakistan	Fruit, root	ND	[17, 40, 41]
*Coccinia grandis*	Pakistan, Sri Lanka	Root	I, E	[17, 18]
*Corallocarpus epigaeus*	India	Tuber	I	[38]
*Cucumis melo*	Sri Lanka	ND	I	[18]
*Cucurbita pepo*	Spain	Flower	E	[37]
*Diplocyclos palmatus*	India, Sri Lanka	Leaf, tuber	I, E	[18, 23, 66]
*Fevillea cordifolia*	Colombia, Nicaragua	Seed, whole plant	I, E	[27, 35]
*Lagenaria siceraria* ^#^	Sri Lanka	ND	E	[18]
*Luffa acutangula*	India, Sri Lanka	Fruit, whole plant	I, E	[18, 19, 38]
*Momordica balsamina*	India	ND	ND	[40]
*Momordica charantia* ^*∗*^	Colombia, India, Nicaragua, Sri Lanka	Aerial parts, branch, flower, fruit, leaf, stem, whole plant	I, E	[18, 20, 27, 34, 35]
*Momordica dioica*	Sri Lanka	ND	E	[18]
*Sicydium tamnifolium*	Mexico	Root	ND	[59]
*Trichosanthes cucumerina*	India, Sri Lanka	Leaf	I	[18, 38]
*Trichosanthes tricuspidata*	Bangladesh	Root	I	
Cycadaceae				
*Cycas pectinata*	Bangladesh	Flower	E	[32]
*Cycas revoluta*	Bangladesh	Whole plant	ND	[30]
Cyclanthaceae				
*Cyclanthus bipartitus*	Peru	Heart	E	[57]
Cyperaceae				
*Cyperus kyllingia*	Sri Lanka	ND	I	[18]
*Cyperus rotundus*	Bangladesh, India, Pakistan, Sri Lanka	Bulb, flower, leaf, rhizome, root, tuber	I, E	[17, 18, 20, 28, 32, 39]
*Kyllinga odorata* (syn. *Kyllinga monocephala*)	India	ND	ND	[40]
Dilleniaceae				
*Tetracera sarmentosa*	Sri Lanka	ND	I, E	[18]
Dioscoreaceae				
*Dioscorea oppositifolia*	Sri Lanka	ND	I	[18]
*Dioscorea pentaphylla*	India	Tuber	I	[38, 55]
Dipterocarpaceae				
*Dipterocarpus lowii*	Sri Lanka	ND	I	[18]
*Dipterocarpus zeylanicus*	Sri Lanka	ND	E	[18]
Droseraceae				
*Drosera burmannii*	Sri Lanka	ND	I, E	[18]
*Drosera indica*	Sri Lanka	ND	E	[18]
Ebenaceae				
*Diospyros kaki*	Malaysia	Fruit	I	[67]
*Diospyros melanoxylon*	India	Seed	E	[25]
*Diospyros montana*	India	Root	I	[38]
*Diospyros vera *(syn. *Maba buxifolia*)	Sri Lanka	ND	I, E	[18]
*Euclea racemosa*	Ethiopia	Leaf	I	[51]
Elaeagnaceae				
*Elaeagnus latifolia*	Sri Lanka	ND	I, E	[18]
Ericaceae				
*Gaultheria trichophylla*	India	Leaf	I	[66]
Erythroxylaceae				
*Erythroxylum monogynum*	Sri Lanka	ND	E	[18]
Euphorbiaceae				
*Acalypha aristata* (syn. *Acalypha arvensis*)	Nicaragua	Leaf, whole plant	I, E	[27]
*Acalypha fimbriata*	ND	ND	ND	[68]
*Acalypha indica* ^*∗*^	Bangladesh, India, Sri Lanka	Leaf, whole plant	E	[18, 20, 32]
*Acalypha phleoides*	Mexico	ND	ND	[68]
*Acalypha wilkesiana *(syn.* Acalypha godseffiana*)	Sri Lanka	ND	E	[18]
*Agrostistachys hookeri*	Sri Lanka	ND	E	[18]
*Baliospermum solanifolium *(syn.* Baliospermum montanum*)	India	Leaf, root, seed	E	[19, 32]
*Cnidoscolus aconitifolius*	Colombia	Leaf, whole plant	I, E	[34]
*Croton tiglium*	Sri Lanka	ND	E	[18]
*Croton trinitatis*	Colombia	Whole plant	E	[34]
*Euphorbia antiquorum*	Sri Lanka	ND	E	[18]
*Euphorbia hirta* ^*∗*^	Bangladesh, Brazil, India	Latex, root, whole plant	I	[19, 20, 32, 47]
*Euphorbia milii*	Bangladesh	Whole plant	ND	[30]
*Euphorbia neriifolia* (syn. *Euphorbia ligularia*)	India, Sri Lanka	Latex, leaf, stem	I, E	[18, 19, 22, 38]
*Euphorbia thymifolia*	Nicaragua	Latex, leaf, whole plant	I	[27]
*Euphorbia tirucalli*	Sri Lanka	ND	I	[18]
*Euphorbia tithymaloides *(syn.* Pedilanthus tithymaloides*)	Sri Lanka	ND	I, E	[18]
*Euphorbia tortilis*	Sri Lanka	ND	E	[18]
*Hura crepitans*	Peru	Latex	E	[57]
*Jatropha curcas* ^*∗*^	Brazil, Nepal	Latex, root, stem	I	[47, 64, 69, 70]
*Jatropha gossypiifolia* ^*∗*^	Bangladesh, Brazil	Latex, leaf, stem	I, E	[32, 50]
*Jatropha mollissima* ^*∗*^	Brazil	Latex	ND	[47, 64]
*Jatropha multifida*	Sri Lanka	ND	E	[18]
*Jatropha podagrica*	Sri Lanka	ND	E	[18]
*Jatropha ribifolia*	Brazil	Latex	ND	[47]
*Mallotus repandus*	Sri Lanka	ND	E	[18]
*Manihot esculenta*	Brazil, Colombia, Nicaragua	Branch, leaf, root	I, E	[27, 33, 34]
*Melanolepis multiglandulosa*	Papua New Guinea	Latex	I	[46]
*Phyllanthus acuminatus* ^#^	Colombia	Branch, leaf	I, E	[35]
*Ricinus communis*	Brazil, Pakistan, Sri Lanka	Fruit, latex, leaf, root, seed	I, E	[17, 18, 69, 71, 72]
*Spirostachys africana*	Namibia	Stem	ND	[65]
*Tragia involucrata*	India	Whole plant	I	[20, 28]
*Trewia nudiflora*	Bangladesh	Leaf	E	[32]
Fabaceae				
*Abrus precatorius* ^*∗*^	Bangladesh, India	Leaf, root, stem	I, E	[20, 21, 28, 32, 38]
*Abrus pulchellus*	Sri Lanka	ND	E	[18]
*Acacia caesia*	Sri Lanka	ND	I, E	[18]
*Acacia cornigera*	Mexico	Root	ND	[59]
*Acacia leucophloea*	India	Bark	I, E	[20, 63]
*Acacia mellifera*	Namibia	ND	ND	[54]
*Acacia nilotica*	India	Leaf	I, E	[38]
*Acacia torta*	India	Bark	I	[63]
*Acosmium panamense*	Mexico	Bark	ND	[59]
*Adenanthera pavonina*	Sri Lanka	ND	I, E	[18]
*Albizia lebbeck* ^*∗*^	Bangladesh, India, Pakistan, Sri Lanka	Bark, flower, fruit, leaf, seed	I, E	[16–18, 23, 32, 40]
*Albizia procera*	Bangladesh, Pakistan	Juicy parts, leaf, root	E	[17, 32]
*Alysicarpus vaginalis*	Sri Lanka	ND	I	[18]
*Amburana cearensis*	Brazil	Seed	ND	[71]
*Bauhinia divaricata *(syn.* Bauhinia retusa*)	India	Bark, flower, leaf	ND	[19]
*Bauhinia guianensis*	Nicaragua	Bark, stem	I, E	[27]
*Bauhinia purpurea*	India	Bark, flower, leaf	ND	[19]
*Bauhinia racemosa*	Sri Lanka	ND	E	[18]
*Bauhinia variegata* ^*∗*^	Bangladesh, Sri Lanka	Bulb, stem	E	[18, 32]
*Brownea *rosa-de-monte^*∗*^	Colombia	Bark	I, E	[35]
*Butea monosperma* ^*∗*^	India	Bark, leaf, resin, seed	I, E	[24, 25, 38, 40, 41]
*Caesalpinia bonduc*	India, Nicaragua, Sri Lanka	Root, seed	I, E	[18, 20, 27, 38]
*Caesalpinia coriaria*	Sri Lanka	ND	E	[18]
*Cajanus cajan*	Bangladesh	Stem	E	[30, 32]
*Canavalia gladiata*	Sri Lanka	ND	E	[18]
*Cassia fistula* ^*∗*^	Bangladesh, Brazil, India, Sri Lanka	Bark, fruit, leaf, root, seed	I, E	[18, 19, 24, 25, 32, 33, 38, 40]
*Centrosema pubescens*	Colombia	Whole plant	E	[34]
*Clitoria ternatea*	Bangladesh, India, Sri Lanka	Flower, leaf, root, seed	I, E	[16, 18, 19, 32, 38, 39, 42, 60]
*Crotalaria laburnifolia*	Sri Lanka	ND	E	[18]
*Crotalaria verrucosa*	India	Seed	I	[23]
*Dalbergia melanoxylon*	India	Bark	I	[20]
*Deguelia amazonica* (syn. *Derris amazonica*)	Brazil	Root	ND	[50]
*Derris floribunda*	Brazil	Root	ND	[50]
*Desmodium adscendens* ^#^	Colombia, Nicaragua	Leaf, root, whole plant	I, E	[27, 35]
*Desmodium gangeticum*	Bangladesh, India, Pakistan	Root, whole plant	I, E	[17, 32, 55]
*Desmodium triflorum*	Bangladesh, Sri Lanka	Shoot	I, E	[18, 32]
*Dipteryx odorata* ^#^	Brazil	Seed	I	[33, 50]
*Entada leptostachya*	Kenya	Latex	E	[31]
*Entada rheedii *(syn. *Entada pursaetha*)	Bangladesh, India, Sri Lanka	Leaf, seed	I, E	[18, 32, 49]
*Erythrina americana*	Mexico	Leaf, seed	ND	[59]
*Erythrina excelsa*	India, Kenya	Bark, latex	ND	[20, 31]
*Erythrina fusca*	Sri Lanka	ND	I, E	[18]
*Erythrina subumbrans*	Sri Lanka	ND	I	[18]
*Erythrina variegata*	India	Bark	ND	[19]
*Gliricidia sepium*	Colombia	Leaf, stem	I, E	[34]
*Glycine max*	India	Seed	I	[20]
*Glycyrrhiza glabra*	Sri Lanka	ND	E	[18]
*Humboldtia decurrens*	India	Root	E	[16]
*Humboldtia laurifolia*	Sri Lanka	ND	E	[18]
*Indigofera circinella*	Kenya	Leaf	E	[31]
*Indigofera suffruticosa*	Colombia, Nicaragua	Aerial parts, seed, whole plant	I, E	[27, 34]
*Indigofera tinctoria* ^#^	India	Root	I	[16]
*Leucaena leucocephala*	Sri Lanka	ND	E	[18]
*Libidibia ferrea* ^#^	Brazil	Seed	I	[33]
*Machaerium ferox*	Brazil	Leaf	E	[33]
*Macrotyloma uniflorum*	Sri Lanka	ND	I	[18]
*Mimosa pudica* ^*∗*^	Bangladesh, India	Leaf, root, whole plant	I, E	[16, 19, 20, 22, 23, 28, 32]
*Mucuna pruriens* ^#^	Bangladesh, India, Nepal, Sri Lanka	Fruit, seed, stem, whole plant	I, E	[18, 19, 28, 32, 69]
*Mucuna sloanei*	Ecuador	Seed	I	[61]
*Mucuna urens*	Nicaragua	Seed	E	[27]
*Parkinsonia aculeata*	Brazil	Seed	ND	[47]
*Pentaclethra macroloba* ^*∗*^	Nicaragua	Bark	I, E	[27]
*Plathymenia reticulata* ^*∗*^	Brazil	Bark	I	[33]
*Pongamia pinnata*	Sri Lanka	ND	I, E	[18]
*Pterocarpus santalinus*	Sri Lanka	ND	E	[18]
*Saraca asoca*	Sri Lanka	ND	I	[18]
*Senna alata *(syn.* Cassia alata*)	India, Nicaragua, Sri Lanka	Flower, leaf, whole plant	I, E	[18, 20, 27, 28]
*Senna auriculata* ^*∗*^	Sri Lanka	ND	E	[18]
*Senna dariensis* ^*∗*^	Colombia	Whole plant	I, E	[35]
*Senna hirsuta*	Bangladesh	Leaf	E	[32]
*Senna occidentalis* (syn. *Cassia occidentalis*)	Bangladesh, India, Nicaragua, Sri Lanka	Leaf, root, whole plant	I, E	[18, 27, 32, 40]
*Senna reticulata *(syn.* Cassia reticulata*)	Brazil, Nicaragua	Leaf, root, whole plant	I	[27, 50]
*Senna siamea*	Kenya	Root	ND	[31]
*Senna sophera *(syn.* Cassia sophera*)	Bangladesh	Leaf, root	I	[30, 32]
*Senna tora *(syn*. Cassia tora*)	Bangladesh, India	Leaf, root, seed, stem	I, E	[20, 24, 25, 28, 32, 42]
*Sesbania grandiflora*	Sri Lanka	ND	I, E	[18]
*Tadehagi triquetrum *(syn.* Desmodium triquetrum*)	India	Whole plant	ND	[19]
*Tamarindus indica* ^*∗*^	Bangladesh, India, Sri Lanka	Seed, whole plant	I, E	[18, 22, 25, 32, 38]
*Tephrosia purpurea*	Bangladesh, India	Root, whole plant	I, E	[19, 20, 24, 32]
*Trigonella foenum-graecum*	Sri Lanka	ND	I	[18]
*Uraria lagopodioides*	India	Bark	I, E	[49]
*Uraria picta*	Bangladesh, India	Root, whole plant	I	[24, 30]
*Vigna luteola*	Colombia	Whole plant	E	[34]
*Vigna radiata*	Sri Lanka	ND	I	[18]
Gentianaceae				
*Chelonanthus alatus *(syn.* Irlbachia alata*)^*#*^	Colombia	Branch, leaf	E	[35]
*Enicostema axillare* ^*∗*^	India	Whole plant	I	[23, 45]
*Fagraea ceilanica*	Sri Lanka	ND	E	[18]
*Hoppea dichotoma*	India	Shoot	ND	[19]
*Huperzia phlegmaria*	Sri Lanka	ND	E	[18]
*Potalia amara*	Peru	Aerial parts	ND	[29]
Gesneriaceae				
*Columnea pulcherrima* ^#^	Colombia	Whole plant	I, E	[35]
*Columnea sanguinea *(syn.* Besleria sanguinea*)^*#*^	Colombia	Whole plant	I, E	[35]
*Episcia dianthiflora* ^#^	Colombia	Whole plant	I, E	[35]
Gleicheniaceae				
*Gleichenella pectinata*	Colombia	Whole plant	I	[34]
Haemodoraceae				
*Xiphidium caeruleum* ^#^	Colombia, Nicaragua, Peru	Leaf, stem, whole plant	I, E	[27, 35, 44, 57]
Heliconiaceae				
*Heliconia curtispatha* ^*∗*^	Colombia	Rhizome	E	[35]
Hydroleaceae				
*Hydrolea zeylanica*	Sri Lanka	ND	I	[18]
Hymenophyllaceae				
*Trichomanes elegans* ^*∗*^	Colombia	Whole plant	E	[35]
Hypoxidaceae				
*Curculigo orchioides*	Bangladesh, India	Bulb, leaf, rhizome	I	[32, 73]
Iridaceae				
*Iris kemaonensis*	India	Rhizome	ND	[66]
*Sisyrinchium micranthum*	Colombia	Whole plant	E	[34]
Lamiaceae				
*Aegiphila panamensis* ^#^	Colombia	Leaf, branch, stem	E	[35]
*Anisochilus velutinus*	Sri Lanka	ND	E	[18]
*Anisomeles indica*	India, Sri Lanka	Whole plant	ND	[18, 19]
*Anisomeles malabarica*	Bangladesh, India	Whole plant	I	[28, 30, 60]
*Callicarpa tomentosa*	Sri Lanka	ND	E	[18]
*Clerodendrum cordatum *(syn.* Clerodendrum viscosum*)	Bangladesh	Flower, leaf	E	[32]
*Clerodendrum phlomidis*	Sri Lanka	ND	E	[18]
*Fuerstia africana*	Kenya	Leaf	I	[31]
*Gmelina arborea*	Bangladesh	Root	I	[32]
*Gmelina asiatica*	Sri Lanka	ND	I, E	[18]
*Hyptis capitata* ^#^	Colombia	Branch, leaf, stem	I, E	[35]
*Hyptis suaveolens*	Bangladesh	Leaf	E	[32]
*Leonotis leonurus*	South Africa	Flower, leaf	I	[74]
*Leucas aspera* ^*∗*^	Bangladesh, India	Leaf, root, stem	I	[23, 24, 28, 30, 32]
*Leucas cephalotes* ^*∗*^	India	Bark, leaf, whole plant	I, E	[19, 20, 40, 49]
*Marsypianthes chamaedrys* ^*∗*^	Brazil	Leaf	I	[33]
*Mentha × piperita*	Colombia	Leaf	E	[34]
*Mentha pulegium*	Colombia	Leaf	E	[34]
*Ocimum basilicum* ^#^	Bangladesh, Colombia, India	Branch, leaf, stem, whole plant	I, E	[20, 32, 35]
*Ocimum campechianum *(syn.* Ocimum micranthum*)	Colombia, Nicaragua	Aerial parts, leaf, whole plant	I, E	[27, 44]
*Ocimum tenuiflorum *(syn.* Ocimum sanctum*)^*∗*^	India, Sri Lanka	Leaf, root, whole plant	I, E	[16, 18, 20, 28, 40, 41]
*Origanum vulgare*	Serbia	Flower, leaf	ND	[58]
*Plectranthus amboinicus*	Sri Lanka	ND	I	[18]
*Plectranthus hadiensis*	Sri Lanka	ND	I	[18]
*Plectranthus monostachyus*	Brazil	Leaf	I	[33]
*Pogostemon cablin*	Malaysia	ND	ND	[75]
*Pogostemon heyneanus*	Sri Lanka	ND	E	[18]
*Premna esculenta*	Bangladesh	Leaf	E	[32]
*Premna serratifolia *(syn.* Premna integrifolia*)	Bangladesh	Leaf, root	I, E	[36]
*Rosmarinus officinalis*	Colombia	Whole plant	E	[34]
*Rotheca serrata *(syn.* Clerodendrum serratum*)	India	Leaf, root	ND	[19, 39]
*Tectona grandis*	India	Bark	I	[25]
*Teucrium chamaedrys*	Serbia	Flower	ND	[58]
*Thymus vulgaris*	India, Spain	Aerial parts, whole plant	I, E	[20, 37]
*Vitex negundo* ^*∗*^	Bangladesh, India, Sri Lanka	Leaf, rhizome, root	I, E	[18, 20, 22, 32]
*Vitex trifolia*	India	Leaf	I	[28]
*Volkameria eriophylla *(syn*. Clerodendrum eriophyllum*)	Kenya	Leaf, root	ND	[76]
Lauraceae				
*Aniba parviflora *(syn.* Aniba fragrans*)^*∗*^	Brazil	Bark	I	[33]
*Cinnamomum verum*	Sri Lanka	ND	I, E	[18]
*Litsea glutinosa*	Sri Lanka	ND	E	[18]
*Litsea longifolia*	Sri Lanka	ND	I, E	[18]
*Persea macrantha*	Sri Lanka	ND	E	[18]
Lecythidaceae				
*Careya arborea*	Sri Lanka	ND	E	[18]
*Couroupita guianensis*	Bangladesh	Bark, leaf	ND	[30]
Linderniaceae				
*Lindernia diffusa* ^#^	Colombia	Whole plant	E	[35]
Loganiaceae				
*Strychnos *nux-vomica^*∗*^	India	Bark, root, seed	I, E	[16, 20, 49]
*Strychnos potatorum*	Sri Lanka	ND	E	[18]
*Strychnos xinguensis* ^*∗*^	Colombia	Stem	E	[35]
Loranthaceae				
*Struthanthus cassythoides*	Nicaragua	Leaf, whole plant	I, E	[27]
*Struthanthus orbicularis* ^*∗*^	Colombia	Branch, leaf	E	[35]
Lycopodiaceae				
*Huperzia pulcherrima*	Sri Lanka	ND	E	[18]
Lygodiaceae				
*Lygodium heterodoxum*	Nicaragua	Leaf	I, E	[27]
*Lygodium venustum*	Colombia, Mexico, Nicaragua	Aerial parts, leaf, stem, whole plant	I, E	[27, 34, 59]
Lythraceae				
*Lawsonia inermis*	India	Bark	ND	[25]
*Punica granatum*	India, Sri Lanka	Whole plant	I, E	[18, 20, 28]
*Trapa natans *(syn.* Trapa bispinosa*)	Sri Lanka	ND	I	[18]
Magnoliaceae				
*Magnolia champaca *(syn.* Michelia champaca*)	Sri Lanka	ND	E	[18]
Malpighiaceae				
*Bronwenia cornifolia *(syn.* Banisteriopsis cornifolia*)	Nicaragua	Bark, leaf, stem	E	[27]
*Byrsonima crassifolia*	Brazil, Nicaragua	Bark, leaf	I	[27, 47]
*Stigmaphyllon puberum*	Nicaragua	Leaf, stem	I, E	[27]
Malvaceae				
*Abelmoschus moschatus*	Bangladesh, India, Sri Lanka	Fruit, leaf, seed	I, E	[18, 32, 38]
*Abroma augusta*	Bangladesh	Leaf, root, stem	E	[32]
*Abutilon hirtum *(syn*. Abutilon heterotrichum*)	Sri Lanka	ND	I, E	[18]
*Abutilon indicum*	India, Sri Lanka	Fruit, leaf	I	[18, 20]
*Ceiba pentandra*	Sri Lanka	ND	I	[18]
*Corchorus trilocularis*	Kenya	Leaf	E	[31]
*Firmiana simplex *(syn.* Sterculia urens*)	India	Bark, latex	I	[38, 55]
*Gossypium arboreum*	Sri Lanka	ND	E	[18]
*Gossypium herbaceum *	India	Seed	ND	[41]
*Gossypium hirsutum*	Brazil	Leaf	I	[33]
*Grewia damine*	Sri Lanka	ND	E	[18]
*Grewia nervosa *(syn.* Microcos paniculata*)	Sri Lanka	ND	E	[18]
*Helicteres isora*	Bangladesh, India	Fruit, root	I	[23, 25, 32]
*Hibiscus rostellatus *(syn.* Hibiscus furcatus*)	Sri Lanka	ND	E	[18]
*Hibiscus surattensis*	Sri Lanka	ND	E	[18]
*Hibiscus tiliaceus*	Mexico	Seed	ND	[59]
*Melochia corchorifolia*	Bangladesh, Sri Lanka	Leaf, whole plant	I, E	[18, 32]
*Sida acuta* ^#^	Bangladesh, Colombia, India, Sri Lanka	Leaf, whole plant	I, E	[18, 32, 35, 39, 44]
*Sida cordata*	Sri Lanka	ND	I	[18]
*Sida cordifolia*	Bangladesh	Leaf	I	[32]
*Sida rhombifolia*	Bangladesh, Nicaragua, Sri Lanka	Leaf, stem	I, E	[18, 27, 32]
*Thespesia populnea*	Sri Lanka	ND	I	[18]
*Triumfetta rhomboidea*	Kenya	Root	E	[31]
*Urena lobata*	Bangladesh	Root	I	[32]
*Wissadula periplocifolia*	Bangladesh, Sri Lanka	Leaf, root	E	[18, 30, 60]
Marantaceae				
*Ischnosiphon rotundifolius*	Brazil	Leaf	ND	[47]
Martyniaceae				
*Martynia annua*	India, Sri Lanka	Fruit	E	[18, 25]
Melastomataceae				
*Osbeckia octandra*	Sri Lanka	ND	E	[18]
*Bellucia dichotoma* ^*∗*^	Brazil	Bark	I	[33]
*Melastoma malabathricum*	Bangladesh	Leaf	E	[32]
*Memecylon umbellatum*	India	Leaf	I	[63]
Meliaceae				
*Azadirachta indica*	India, Sri Lanka	Bark, flower, latex, leaf, seed	I, E	[18, 20, 22, 28, 39–41]
*Cipadessa baccifera*	India	Leaf, root	I	[63]
*Melia azedarach*	India, Sri Lanka	Bark, leaf	I, E	[18, 41]
*Munronia pinnata*	Sri Lanka	ND	I, E	[18]
Menispermaceae				
*Cissampelos fasciculata*	Colombia	Leaf	I	[44]
*Cissampelos pareira* ^*∗*^	Bangladesh, India, Mexico, Nicaragua, Sri Lanka	Leaf, root, whole plant	I, E	[18, 19, 23, 25, 27, 32, 38, 55, 59]
*Cocculus acuminatus*	India	Stem	E	[16]
*Cocculus hirsutus *(syn.* Cocculus villosus*)	India	Leaf	I	[38, 40]
*Coscinium fenestratum*	Sri Lanka	ND	I	[18]
*Cyclea peltata*	Sri Lanka	ND	I	[18]
*Odontocarya tenacissima* ^#^	Colombia	Whole plant	I, E	[35]
*Tinospora cordifolia*	Bangladesh, India, Sri Lanka	Fruit, root, stem	I	[18, 22, 23, 32]
Menyanthaceae				
*Nymphoides indica*	Nicaragua, Sri Lanka	Leaf, root	I, E	[18, 27]
Monimiaceae				
*Hortonia angustifolia*	Sri Lanka	ND	E	[18]
Moraceae				
*Artocarpus heterophyllus*	Sri Lanka	ND	E	[18]
*Artocarpus nobilis*	Sri Lanka	ND	I, E	[18]
*Broussonetia zeylanica*	Sri Lanka	ND	I, E	[18]
*Castilla elastica* ^*∗*^	Colombia	Branch, leaf, stem	I, E	[35]
*Dorstenia contrajerva*	Mexico, Nicaragua	Leaf, whole plant	I, E	[27, 59]
*Ficus benghalensis*	India	ND	ND	[40]
*Ficus drupacea*	Sri Lanka	ND	E	[18]
*Ficus hispida*	Sri Lanka	ND	E	[18]
*Ficus nymphaeifolia* ^*∗*^	Colombia	Branch, leaf, stem	I, E	[35]
*Ficus racemosa*	Bangladesh, India, Sri Lanka	Bark, shoot	I, E	[18, 32, 38]
*Ficus religiosa*	India, Sri Lanka	Bark	I, E	[18, 49]
*Morus alba* ^*∗*^	India	Leaf	I	[20]
*Plecospermum spinosum*	Sri Lanka	ND	I, E	[18]
*Streblus asper*	Bangladesh	Root	E	[32]
Moringaceae				
*Moringa oleifera* ^#^	India, Sri Lanka	Bark, root, seed	I, E	[16, 18, 20, 22, 24, 28]
Musaceae				
*Ensete ventricosum *(syn.* Ensete edule*)	Kenya	Latex	E	[31]
*Musa × paradisíaca* ^*∗*^	Ecuador, India, Nicaragua, Sri Lanka	Bark, flower, latex	I, E	[18, 20, 27, 28, 61]
Myristicaceae				
*Myristica fragrans*	Sri Lanka	ND	I	[18]
Myrtaceae				
*Myrcia bracteata *(syn. *Eugenia bracteata*)	Sri Lanka	ND	I, E	[18]
*Syzygium aromaticum*	Sri Lanka	ND	I	[18]
*Syzygium caryophyllatum*	Sri Lanka	ND	E	[18]
*Syzygium cumini *(syn.* Eugenia jambolana*)	India, Pakistan, Sri Lanka	Bark, leaf	I	[17, 18, 20]
*Syzygium zeylanicum*	Sri Lanka	ND	E	[18]
Nelumbonaceae				
*Nelumbo nucifera*	Sri Lanka	ND	I	[18]
Nepenthaceae				
*Nepenthes distillatoria*	Sri Lanka	ND	E	[18]
Nyctaginaceae				
*Boerhavia coccinea*	Pakistan	Whole plant	E	[17]
*Boerhavia diffusa*	Brazil, India, Sri Lanka	Leaf, root, whole plant	E	[18, 24, 25, 39, 41, 50]
*Boerhavia procumbens*	Pakistan	Leaf	E	[17]
*Mirabilis jalapa*	Bangladesh, Sri Lanka	Leaf	I, E	[18, 32]
Nymphaeaceae				
*Nymphaea nouchali*	Sri Lanka	ND	E	[18]
*Nymphaea pubescens*	Sri Lanka	ND	I	[18]
Ochnaceae				
*Ochna jabotapita*	Sri Lanka	ND	I	[18]
*Sauvagesia erecta*	Nicaragua	Whole plant	I, E	[27]
Oleaceae				
*Jasminum officinale*	Sri Lanka	ND	E	[18]
*Jasminum sambac*	Sri Lanka	ND	E	[18]
*Nyctanthes arbor-tristis*	India, Sri Lanka	Root	I	[18, 49]
*Olea europaea*	Spain	Oil	ND	[37]
Opiliaceae				
*Opilia amentacea*	Kenya	Root	E	[31]
Orchidaceae				
*Vanda tessellata*	India	Root	E	[25]
*Zeuxine regia*	Sri Lanka	ND	E	[18]
Oxalidaceae				
*Averrhoa carambola*	Sri Lanka	ND	I	[18]
*Biophytum reinwardtii*	Sri Lanka	ND	I	[18]
*Oxalis corniculata*	Bangladesh, Sri Lanka	Leaf	I, E	[18, 32]
Pandanaceae				
*Pandanus kaida*	Sri Lanka	ND	I	[18]
*Pandanus odorifer *(syn.* Pandanus odoratissimus*)	India	Root	ND	[19]
Papaveraceae				
*Argemone mexicana*	Bangladesh, India	Leaf, root, seed, stem	I, E	[20, 32, 38, 42]
Papilionaceae				
*Desmodium elegans*	Pakistan	Root	E	[17, 53]
Passifloraceae				
*Adenia hondala*	Sri Lanka	ND	E	[18]
*Passiflora quadrangularis* ^*∗*^	Colombia	Branch, leaf	E	[34, 35]
Phyllanthaceae				
*Antidesma bunius*	India	Leaf	ND	[77]
*Bridelia retusa*	Sri Lanka	ND	I, E	[18]
*Cleistanthus collinus*	Sri Lanka	ND	I	[18]
*Glochidion zeylanicum*	Sri Lanka	ND	I	[18]
*Margaritaria indica*	Sri Lanka	ND	I, E	[18]
*Phyllanthus acidus*	India	Root	ND	[77]
*Phyllanthus debilis*	Sri Lanka	ND	I	[18]
*Phyllanthus emblica *(syn.* Emblica officinalis*)^*∗*^	Bangladesh, India, Sri Lanka	Bark, fruit, root	I, E	[18, 20, 22, 30]
*Phyllanthus niruri*	India	Flower	E	[20]
*Phyllanthus reticulatus*	India	Leaf	I	[20]
*Phyllanthus urinaria*	Sri Lanka	ND	I, E	[18]
Phytolaccaceae				
*Petiveria alliacea* ^#^	Colombia, Nicaragua	Branch, leaf, root, whole plant	I, E	[27, 34, 35]
Pinaceae				
*Pinus roxburghii* ^*∗*^	Pakistan	Oil, resin, wood	E	[17, 53]
Piperaceae				
*Peperomia elsana* ^#^	Colombia	Whole plant	E	[35]
*Peperomia pellucida*	Nicaragua, Sri Lanka	Whole plant	I, E	[18, 27]
*Piper amalago*	Mexico, Nicaragua	Leaf, root	I	[27, 59]
*Piper arboreum* ^*∗*^	Colombia	Branch, leaf	E	[35]
*Piper auritum* ^#^	Colombia, Nicaragua	Branch, leaf, stem, whole plant	I, E	[27, 34, 35, 44]
*Piper betle*	Sri Lanka	ND	I, E	[18]
*Piper chuvya*	Sri Lanka	ND	E	[18]
*Piper confusionis*	Peru	Leaf	E	[57]
*Piper coruscans* ^#^	Colombia	Branch, leaf, stem	I, E	[35]
*Piper hispidum* ^#^	Colombia	Branch, leaf, stem	I, E	[35]
*Piper longivillosum* ^#^	Colombia	Whole plant	E	[35]
*Piper longum* ^*∗*^	Bangladesh, Sri Lanka	Flower, fruit, Latex, root	E	[18, 30]
*Piper marginatum* ^#^	Brazil, Colombia	Branch, leaf, root, stem	I, E	[35, 50]
*Piper multiplinervium* ^#^	Colombia	Branch, leaf, stem	I, E	[35]
*Piper nigrum*	Bangladesh, India, Sri Lanka	Floral bud, flower, fruit, root	I, E	[18, 20, 28, 32, 52]
*Piper peltatum* ^#^	Colombia, Nicaragua	Branch, leaf, stem, whole plant	I, E	[27, 35]
*Piper pulchrum* ^*∗*^	Colombia	Branch, leaf, stem	I, E	[35]
*Piper reticulatum* ^#^	Colombia	Branch, leaf, stem	I, E	[35]
*Piper tricuspe* ^#^	Colombia	Branch, leaf, stem	E	[35]
*Piper umbellatum*	Sri Lanka	ND	I, E	[18]
Pittosporaceae				
*Pittosporum neelgherrense*	India	Bark	I, E	[16]
*Pittosporum tetraspermum*	India	Bark	I	[26]
Plantaginaceae				
*Bacopa monnieri*	Bangladesh, India, Sri Lanka	Leaf, root, whole plant	I	[18, 23, 32, 39, 41]
*Plantago australis*	Colombia	Whole plant	E	[34]
*Plantago major*	Colombia	Aerial parts, leaf	I, E	[44]
*Scoparia dulcis* ^#^	Colombia, Nicaragua	Aerial parts, branch, leaf, root, whole plant	I, E	[27, 34, 35, 44]
Platanaceae				
*Platanus orientalis*	Pakistan	Bark	I, E	[17]
Plumbaginaceae				
*Plumbago indica*	Sri Lanka	ND	I, E	[18]
*Plumbago zeylanica*	Bangladesh, India, Sri Lanka	Root	I, E	[18, 23, 32]
Poaceae				
*Chrysopogon zizanioides *(syn. *Vetiveria zizanioides*)	India, Sri Lanka	Root	I, E	[16, 18]
*Cymbopogon citratus*	Colombia	Leaf	E	[34]
*Cynodon dactylon*	Bangladesh, India, Sri Lanka	Leaf, root, whole plant	E	[18, 19, 32]
*Drynaria quercifolia*	Sri Lanka	ND	I	[18]
*Eleusine coracana*	Sri Lanka	ND	I	[18]
*Gynerium sagittatum*	Nicaragua	Leaf, root	I	[27]
*Heteropogon contortus*	India, Sri Lanka	Root	I, E	[18, 38, 55]
*Isachne globosa*	Sri Lanka	ND	E	[18]
*Oryza punctata*	Sri Lanka	ND	I, E	[18]
*Oryza sativa*	Sri Lanka	ND	I	[18]
*Pogonatherum paniceum*	Sri Lanka	ND	E	[18]
*Saccharum arundinaceum*	Sri Lanka	ND	I	[18]
*Saccharum officinarum*	Colombia, Sri Lanka	Stem	I, E	[18, 34, 44]
Polygalaceae				
*Polygala abyssinica*	Pakistan	Root	I	[17]
*Polygala crotalarioides*	India	Leaf, root	ND	[19]
*Polygala paniculata*	Brazil	Root	E	[47]
*Polygala spectabilis*	Brazil	Root	I, E	[47]
Polygonaceae				
*Persicaria barbata *(syn.* Polygonum barbatum*)	India	Leaf	I, E	[38]
*Persicaria chinensis *(syn*. Polygonum chinense*)	Bangladesh	Leaf	E	[32]
*Persicaria ferruginea *(syn.* Polygonum ferrugineum*)	Colombia	Aerial parts	E	[34]
*Persicaria glabra *(syn.* Polygonum glabrum*)	India	Root	E	[25]
Polypodiaceae				
*Pleopeltis percussa* ^*∗*^	Colombia	Branch, leaf, stem	I, E	[35]
*Pyrrosia piloselloides*	Sri Lanka	ND	E	[18]
Pontederiaceae				
*Monochoria hastata*	Sri Lanka	ND	I, E	[18]
Portulacaceae				
*Portulaca pilosa*	Brazil	Leaf	I	[33]
Primulaceae				
*Aegiceras corniculatum*	Sri Lanka	ND	E	[18]
*Anagallis arvensis*	Serbia	Aerial parts	ND	[58]
*Ardisia humilis*	Sri Lanka	ND	E	[18]
*Maesa lanceolata* ^*∗*^	Kenya	Root	ND	[31]
*Myrsine coriacea*	Colombia	Whole plant	E	[34]
Pteridaceae				
*Acrostichum aureum*	Nicaragua	Leaf, root	I, E	[27]
*Adiantum capillus-veneris*	Pakistan	Frond	E	[17]
*Pellaea viridis*	Kenya	Leaf	E	[31]
Ranunculaceae				
*Clematis brachiata *(syn.* Clematis triloba*)	India	Root	E	[25]
*Delphinium denudatum*	India	Root	ND	[19]
*Delphinium vestitum*	India	Whole plant	ND	[19]
Rhamnaceae				
*Alphitonia incana*	Papua New Guinea	Oil	E	[46]
*Ziziphus jujuba *(syn.* Ziziphus mauritiana*)	Sri Lanka	ND	E	[18]
*Ziziphus oenoplia*	India, Sri Lanka	Leaf	I, E	[18, 49]
Rhizophoraceae				
*Rhizophora mangle*	Nicaragua	Bark	I, E	[27]
Rosaceae				
*Crataegus monogyna*	Spain	Thorn	ND	[37]
*Potentilla sundaica*	India	Root, stem	ND	[19]
*Prunus persica*	Ethiopia	Leaf	I	[51]
*Prunus walkeri*	Sri Lanka	ND	E	[18]
*Pyrus communis*	Pakistan	Fruit, leaf	I	[17]
*Sanguisorba officinalis*	Serbia	Rhizome	ND	[58]
Rubiaceae				
*Catunaregam spinosa *(syn. *Randia dumetorum*)	India	Root	I	[23]
*Ceriscoides turgida *(syn*. Gardenia turgida*)	India	Bark, root	I	[24, 38]
*Chiococca alba*	Brazil, Nicaragua	Leaf, root	I	[27, 47]
*Clausena dentata*	Sri Lanka	ND	E	[18]
*Gonzalagunia panamensis* ^*∗*^	Colombia	Branch, leaf, stem	I, E	[35]
*Hamelia axillaris*	Nicaragua	Leaf, whole plant	I, E	[27]
*Hamelia barbata*	Nicaragua	Leaf, whole plant	I, E	[27]
*Hamelia patens*	Nicaragua	Leaf, whole plant	I, E	[27]
*Hamelia rovirosae*	Nicaragua	Flower, leaf, stem	I, E	[27]
*Hedyotis scandens*	Bangladesh	Leaf, stem	E	[32]
*Ixora coccinea*	Sri Lanka	ND	I, E	[18]
*Ixora cuneifolia*	Bangladesh	Bark	E	[32]
*Ixora pavetta *(syn.* Ixora arborea*)	India	Leaf, rood, seed	ND	[19]
*Mitragyna parvifolia*	India	Bark, stem	I, E	[38, 63]
*Morinda angustifolia*	Bangladesh	Leaf	I	[32]
*Morinda citrifolia*	Bangladesh	Root	ND	[30]
*Morinda coreia*	Sri Lanka	ND	I, E	[18]
*Morinda persicifolia*	Bangladesh	Leaf	E	[32]
*Mussaenda frondosa*	Sri Lanka	ND	I	[18]
*Mussaenda roxburghii*	Bangladesh	Leaf	E	[32]
*Nauclea orientalis*	Sri Lanka	ND	E	[18]
*Neonauclea purpurea *(syn. *Anthocephalus chinensis*)	Bangladesh	Bark, leaf	ND	[30]
*Oldenlandia diffusa*	India	Whole plant	E	[20]
*Oldenlandia umbellata*	India	Leaf, root	E	[20]
*Ophiorrhiza mungos* ^*∗*^	India	Root	I	[16, 20]
*Paederia foetida*	Sri Lanka	ND	I, E	[18]
*Palicourea croceoides*	Colombia	Bark	I	[34]
*Pavetta indica*	Sri Lanka	ND	I, E	[18]
*Psychotria elata*	Nicaragua	Flower, leaf, root, stem, whole plant	I, E	[27]
*Psychotria flavida*	India	Root	I	[63]
*Psychotria poeppigiana* ^#^	Colombia, Nicaragua, Sri Lanka	Branch, leaf, stem, whole plant	I, E	[18, 27, 35]
*Randia aculeata* ^*∗*^	Mexico	Fruit, whole plant	I	[59, 78]
*Rubia cordifolia* ^*∗*^	Nepal, Pakistan	Leaf, root, stem	I	[17, 69]
*Rubia manjith*	India	Root, stem	ND	[19]
*Spermacoce remota *(syn.* Borreria assurgens*)	Nicaragua	Leaf, root	I, E	[27]
*Tamilnadia uliginosa*	Sri Lanka	ND	I	[18]
*Wendlandia exserta*	India	Root	I	[49]
Rutaceae				
*Acronychia pedunculata*	Sri Lanka	ND	E	[18]
*Aegle marmelos*	Bangladesh, India, Sri Lanka	Bark, whole plant	I, E	[18, 20, 30, 32, 41]
*Atalantia ceylanica*	Sri Lanka	ND	I, E	[18]
*Citrus aurantiifolia*	Sri Lanka	ND	I, E	[18]
*Citrus aurantium*	Sri Lanka	ND	I, E	[18]
*Citrus japonica *(syn*. Citrus madurensis*)	Sri Lanka	ND	I, E	[18]
*Citrus limon* ^*∗*^	Colombia, India, Sri Lanka	Fruit, leaf, root	I, E	[18, 20, 28, 34, 35]
*Citrus maxima *(syn*. Citrus grandis*)	Sri Lanka	ND	I, E	[18]
*Glycosmis pentaphylla*	India	Leaf	I, E	[16]
*Limonia acidissima *(syn.* Feronia limonia*)	India, Sri Lanka	Root	I	[18, 20]
*Murraya koenigii*	India, Sri Lanka	Bark, leaf	I, E	[18, 28]
*Murraya paniculata* ^*∗*^	Sri Lanka	ND	E	[18]
*Naringi crenulata*	India	Fruit	ND	[19]
*Pamburus missionis*	Sri Lanka	ND	E	[18]
*Ruta chalepensis*	Colombia	Whole plant	E	[34]
*Toddalia asiatica*	India, Sri Lanka	Root	I, E	[18, 63]
Salicaceae				
*Casearia grandiflora* ^*∗*^	ND	Bark, leaf	ND	[79]
*Casearia nigrescens *(syn.* Casearia elliptica*)	India	Bark, leaf	ND	[19]
*Casearia sylvestris* ^*∗*^	Brazil	Leaf, whole plant	ND	[47, 79]
*Casearia tomentosa*	India	Bark, root	I, E	[49, 79]
*Flacourtia indica*	Bangladesh	Leaf	E	[32]
Santalaceae				
*Santalum album*	Sri Lanka	ND	E	[18]
Sapindaceae				
*Allophylus cobbe*	Sri Lanka	ND	I, E	[18]
*Cardiospermum halicacabum*	India, Sri Lanka	Leaf	I, E	[18, 28]
*Dodonaea viscosa*	India	Leaf	E	[28]
*Harpullia arborea*	Sri Lanka	ND	I, E	[18]
*Sapindus emarginatus*	India	Bark	I	[20]
*Sapindus mukorossi*	India, Pakistan	Fruit, leaf, root, seed	E	[17, 25]
Sapotaceae				
*Madhuca longifolia *(syn.* Madhuca indica*)	India, Sri Lanka	Bark, fruit, leaf, nut, root, seed	I, E	[18, 20, 23, 25, 32, 38]
*Manilkara zapota*	Mexico	Root	ND	[59]
*Mimusops elengi*	Sri Lanka	ND	I	[18]
Scrophulariaceae				
*Verbascum thapsus*	India	Leaf	ND	[66]
Selaginellaceae				
*Selaginella articulata* ^#^	Colombia	Whole plant	I, E	[35]
Simaroubaceae				
*Ailanthus excelsa*	India	Bark	I	[38]
*Quassia amara* ^#^	Colombia, Nicaragua	Root, stem, whole plant	I, E	[27, 35]
*Quassia indica*	Sri Lanka	ND	I	[18]
*Simaba cedron* ^#^	Colombia	Seed, whole plant	I, E	[34, 35, 44]
Siparunaceae				
*Siparuna gesnerioides*	Colombia	Leaf, root	I	[34, 44]
*Siparuna thecaphora* ^*∗*^	Colombia	Branch, leaf, stem	I, E	[35]
Smilacaceae				
*Smilax regelii*	Nicaragua	Root	I	[27]
*Smilax spinosa*	Nicaragua	Root	I	[27]
Solanaceae				
*Atropa acuminata*	Pakistan	Leaf, root	E	[17]
*Capsicum annuum *(syn.* Capsicum frutescens*)^*∗*^	Bangladesh, Colombia, India, Sri Lanka	Fruit, root	I, E	[18, 19, 25, 32, 34, 35]
*Datura metel*	Bangladesh, Colombia, India, Sri Lanka	Bark, flower, fruit, leaf, root, seed	I, E	[18, 22, 23, 25, 28, 30, 34]
*Datura stramonium* ^#^	India	Root	I, E	[38]
*Lycopersicon esculentum*	Colombia	Leaf, stem, whole plant	E	[34]
*Nicotiana tabacum*	Colombia, India, Nicaragua	Leaf	I, E	[20, 27, 44]
*Solanum allophyllum* ^#^	Colombia	Branch, leaf, stem	I, E	[35]
*Solanum americanum *(syn.* Solanum nigrum*)	Colombia, India, Sri Lanka	Fruit, leaf, whole plant	I, E	[18, 25, 34, 38]
*Solanum capsicoides*	Bangladesh	Seed	ND	[30]
*Solanum incanum*	Kenya	Fruit, stem	E	[31]
*Solanum melongena*	Sri Lanka	ND	I	[18]
*Solanum nudum* ^#^	Colombia	Branch, fruit, leaf, stem	I, E	[35]
*Solanum ochraceo-ferrugineum*	Mexico	Whole plant	ND	[59]
*Solanum torvum*	Bangladesh, India, Nicaragua	Flower, leaf, root	I, E	[20, 27, 30, 32]
*Solanum verbascifolium*	Mexico	Whole plant	ND	[59]
*Solanum virginianum *(syn. *Solanum xanthocarpum*)	India, Sri Lanka	Root	I	[18, 41]
*Withania somnifera*	Bangladesh, India, Sri Lanka	Root	I, E	[18, 22, 32]
Sterculiaceae				
*Byttneria pilosa*	Bangladesh	Leaf, stem	E	[32]
Symplocaceae				
*Symplocos cochinchinensis*	Sri Lanka	ND	E	[18]
*Symplocos racemosa*	Sri Lanka	ND	I, E	[18]
Talinaceae				
*Talinum paniculatum*	Sri Lanka	ND	E	[18]
Thymelaeaceae				
*Daphne papyracea*	Pakistan	Leaf, root, stem	ND	[17]
Triuridaceae				
*Sciaphila purpurea*	Colombia	Whole plant	I, E	[35]
Urticaceae				
*Boehmeria nivea*	Sri Lanka	ND	E	[18]
*Cecropia obtusifolia*	Nicaragua	Leaf	I	[27]
*Cecropia peltata*	Bangladesh, Nicaragua	Leaf, whole plant	I	[27, 30]
*Girardinia diversifolia*	Sri Lanka	ND	E	[18]
*Pouzolzia zeylanica *(syn.* Pouzolzia indica*)	Bangladesh, India	Leaf, whole plant	E	[32, 42]
Verbenaceae				
*Aloysia triphylla*	Colombia	Aerial parts	E	[34]
*Lantana camara*	Bangladesh, Colombia, India, Sri Lanka	Flower, leaf, root, stem	I, E	[18, 32, 34, 39, 41]
*Lippia alba*	Colombia	Aerial parts, leaf, whole plant	I, E	[34]
*Lippia grandis*	Brazil	Leaf	I	[33]
*Stachytarpheta cayennensis*	Colombia	Whole plant	E	[34]
*Verbena litoralis*	Colombia	Fruit peel, leaf, root, seed	E	[34]
*Verbena officinalis*	Pakistan	Root, whole plant	E	[17, 53]
Vitaceae				
*Ampelocissus latifolia*	India	Root	I	[49]
*Cayratia pedata *(syn. *Cissus pedata*)	Bangladesh	Leaf	ND	[30]
*Cayratia trifolia *(syn. *Vitis trifolia*)	Bangladesh, India	Leaf, root	I, E	[36, 38]
*Cissus adnata*	Bangladesh	Leaf	E	[32]
*Cissus javana*	Bangladesh	Leaf, stem	E	[32]
*Cissus quadrangularis* ^#^	Sri Lanka	ND	E	[18]
*Leea indica*	Sri Lanka	ND	E	[18]
*Vitis heyneana *(syn.* Vitis lanata*)	Bangladesh	Leaf	E	[32]
Xanthorrhoeaceae				
*Aloe harlana*	Ethiopia	Leaf	I	[51]
*Aloe littoralis*	Pakistan	Whole plant	E	[17]
*Aloe vera*	Nicaragua, Sri Lanka	Leaf	I, E	[18, 27]
Zingiberaceae				
*Alpinia calcarata*	Sri Lanka	ND	I, E	[18]
*Alpinia galanga*	Sri Lanka	ND	I	[18]
*Alpinia nigra*	Sri Lanka	ND	E	[18]
*Alpinia purpurata*	Colombia	Leaf	E	[44]
*Curcuma angustifolia*	India	Rhizome	E	[28]
*Curcuma longa* ^*∗*^	Bangladesh, India, Sri Lanka	Rhizome	I, E	[16, 18, 20, 30]
*Elettaria cardamomum*	Sri Lanka	ND	I	[18]
*Globba marantina *(syn.* Globba bulbifera*)	India	Rhizome	I	[49]
*Hedychium coronarium*	Colombia, Nicaragua	Root, whole plant	E	[27, 34]
*Renealmia alpinia* ^*∗*^	Colombia	Rhizome, stem	I, E	[34, 35]
*Renealmia thyrsoidea*	Colombia	Leaf, stem	I	[34]
*Zingiber officinale* ^*∗*^	Ecuador, Nicaragua, Sri Lanka	Rhizome, root	I, E	[18, 27, 61]
Zygophyllaceae				
*Balanites aegyptiaca* ^#^	India	Bark, fruit	E	[38]

In parentheses is the synonym used in the original work; out of the parentheses is the accepted name (in case of more than one paper treating the same species with different names); ND = information not described in the work; I = internal use; E = external use. ^*∗*^Species evaluated on antiophidic activities in previous studies (see Tables 2–8) showing good inhibitory potential against venom induced local effects. ^#^Species evaluated on antiophidic activities in previous studies, however, with poor inhibition potential against venom induced local effects.

**Table 2:** List of medicinal plants with inhibitory potential against local effects induced by *Naja *snakes.

Plant name	Part used	Snake venom	Inhibited activities		Reference(s)
			*In vitro*	*In vivo*	
Acanthaceae					
*Andrographis stenophylla*	Leaf	*N. naja*	—	Hemorrhage	[83]
Amaranthaceae					
*Pupalia lappacea*	Herbal	*N. nigricollis*	SVH	—	[82]
Amaryllidaceae					
*Allium cepa* ^#^	Bulb	*N. n. karachiensis*	PLA_2_	—	[84]
*Allium sativum* ^#^	Bulb	*N. n. karachiensis*	PLA_2_	—	[84]
Anacardiaceae					
*Lannea acida*	Cortex	*N. nigricollis*	SVH	—	[82]
*Pistacia chinensis *subsp.*integerrima*^#^	Gall	*N. n. karachiensis*	PLA_2_	—	[84]
*Sclerocarya birrea*	Cortex	*N. nigricollis*	SVH	—	[82]
*Spondias mombin* ^#^	Cortex, radix	*N. nigricollis*	SVH	—	[82]
Annonaceae					
*Annona senegalensis* ^#^	Cortex	*N. nigricollis*	SVH	—	[82]
Apiaceae					
*Cuminum cyminum*	Seed	*N. n. karachiensis*	PLA_2_	—	[84]
Apocynaceae					
*Acokanthera oppositifolia*	Radix	*N. nigricollis*	SVH	—	
*Calotropis procera* ^#^	Flower, latex	*N. n. karachiensis*	PLA_2_	—	[84]
*Strophanthus sarmentosus*	Folium	*N. nigricollis*	SVH	—	[82]
*Strophanthus speciosus*	Radix	*N. nigricollis*	SVH	—	[82]
*Tylophora indica* ^#^	Leaf, root	*N. naja*	PLA_2_	Hemorrhage	[85]
Araceae					
*Colocasia esculenta*	Tuber	*N. nigricollis*	SVH	—	[82]
Araliaceae					
*Polyscias fulva*	Cortex	*N. nigricollis*	SVH	—	[82]
Aristolochiaceae					
*Aristolochia bracteolata* ^#^	Leaf, root	*N. naja*	PLA_2_	Hemorrhage	[85]
Asteraceae					
*Callilepis laureola*	Radix	*N. nigricollis*	SVH	—	[82]
Bignoniaceae					
*Kigelia africana*	Cortex, folium	*N. nigricollis*	SVH	—	[82]
*Tecoma stans *(syn. *Stenolobium stans*)	Root	*N. n. karachiensis*	PLA_2_	—	[84]
Bixaceae					
*Cochlospermum tinctorium*	Radix	*N. nigricollis*	SVH	—	[82]
Boraginaceae					
*Cordia macleodii*	Bark	*N. naja*	—	Edema, hemorrhage^*∗*^, necrosis^*∗*^	[86]
*Trichodesma indicum* ^#^	Whole plant	*N. n. karachiensis*	PLA_2_	—	[84]
Capparaceae					
*Capparis tomentosa*	Radix	*N. nigricollis*	SVH	—	[82]
Colchicaceae					
*Gloriosa superba* ^#^	Radix	*N. nigricollis*	SVH	—	[82]
Combretaceae					
*Combretum molle* ^#^	Folium	*N. nigricollis*	SVH	—	[82]
*Guiera senegalensis*	Radix	*N. nigricollis*	SVH	—	[82]
*Terminalia arjuna* ^#^	Bark	*N. n. karachiensis*	PLA_2_	—	[84]
Convolvulaceae					
*Ipomoea rubens*	Seed	*N. nigricollis*	SVH	—	[82]
Cucurbitaceae					
*Citrullus colocynthis* ^#^	Fruit	*N. n. karachiensis*	PLA_2_	—	[84]
*Luffa cylindrica *(syn.* Luffa aegyptiaca*)	Leaf	*N. nigricollis*	Proteolytic	—	[87]
*Momordica charantia* ^#^	Fruit	*N. n. karachiensis*	PLA_2_	—	[84]
Ebenaceae					
*Diospyros mespiliformis*	Cortex	*N. nigricollis*	SVH	—	[82]
Euphorbiaceae					
*Alchornea laxiflora*	Cortex	*N. nigricollis*	SVH	—	[82]
*Clutia cordata*	Radix	*N. nigricollis*	SVH	—	[82]
*Euphorbia hirta* ^#^	Whole plant	*N. naja*	PLA_2_, proteolytic, SVH	Edema^*∗*^	[88]
*Jatropha curcas* ^#^	Leaf, root, stem	*N. naja*	PLA_2_	—	[89]
*Jatropha gossypiifolia* ^#^	Leaf, root, stem	*N. naja*	PLA_2_	—	[89]
*Manihot foetida *(syn.* Jatropha foetida*)	Leaf, stem	*N. naja*	PLA_2_	—	[89]
Fabaceae					
*Abrus precatorius* ^#^	Radix	*N. nigricollis*	SVH	—	[82]
*Argyrolobium stipulaceum*	Radix	*N. nigricollis*	SVH	—	[82]
*Bauhinia thonningii*	Cortex, radix	*N. nigricollis*	SVH	—	[82]
*Bauhinia variegata* ^#^	Root	*N. n. karachiensis*	PLA_2_	—	[84]
*Bobgunnia madagascariensis *(syn.* Swartzia madagascariensis*)	Folium, radix	*N. nigricollis*	SVH	—	[82]
*Burkea africana*	Cortex	*N. nigricollis*	SVH	—	[82]
*Cullen corylifolium *(syn.* Psoralea corylifolia*)	Seed	*N. n. karachiensis*	PLA_2_	—	[84]
*Dichrostachys cinerea*	Folium	*N. nigricollis*	SVH	—	[82]
*Entada africana*	Radix	*N. nigricollis*	SVH	—	[82]
*Mimosa pudica* ^#^	Root	*N. kaouthia, N. naja*	PLA_2_, proteolytic, SVH	Edema^*∗*^, myotoxicity^*∗*^	[90–92]
*Parkia biglobosa*	Cortex, stem bark	*N. nigricollis*	Cytotoxicity against muscle cells, SVH		[82, 93]
*Stylosanthes erecta*	Folium	*N. nigricollis*	SVH	—	[82]
*Tamarindus indica* ^#^	Folium, radix	*N. nigricollis*	SVH	—	[82]
Gentianaceae					
*Enicostema axillare *(syn*. Enicostema hyssopifolium*)^*#*^	Whole plant	*N. n. karachiensis*	PLA_2_	—	[84]
Hypericaceae					
*Psorospermum corymbiferum*	Cortex, radix	*N. nigricollis*	SVH	—	[82]
Lamiaceae					
*Leucas aspera* ^#^	Leaf, root, whole plant	*N. naja*	PLA_2_, proteolytic, SVH	Hemorrhage	[85, 94]
*Leucas cephalotes *(syn.* Leucas capitata*)^*#*^	Whole plant	*N. n. karachiensis*	PLA_2_	—	[84]
*Leucas martinicensis*	ND	*N. nigricollis*	SVH	—	[82]
*Ocimum tenuiflorum *(syn.* Ocimum sanctum*)^*#*^	Whole plant	*N. n. karachiensis*	PLA_2_	—	[84]
*Rotheca myricoides *(syn*. Clerodendrum myricoides*)	Cortex	*N. nigricollis*	SVH	—	[82]
*Teucrium kraussii*	Aerial parts, cortex	*N. nigricollis*	SVH	—	[82]
*Volkameria glabra *(syn.* Clerodendrum glabrum*)	Radix	*N. nigricollis*	SVH	—	[82]
Lauraceae					
*Cassytha filiformis*	Herbal	*N. nigricollis*	SVH	—	[82]
Loganiaceae					
*Strychnos innocua*	Folium	*N. nigricollis*	SVH	—	[82]
*Strychnos *nux-vomica^#^	Seed	*N. kaouthia*	PLA_2_	—	[95]
Malvaceae					
*Althaea officinalis*	Root	*N. n. karachiensis*	PLA_2_	—	[84]
*Dombeya quinqueseta*	Cortex	*N. nigricollis*	SVH	—	[82]
*Grewia mollis*	Cortex, folium, radix	*N. nigricollis*	SVH	—	[82]
*Sterculia setigera*	Cortex	*N. nigricollis*	SVH	—	[82]
*Waltheria indica*	Radix	*N. nigricollis*	SVH	—	[82]
Menispermaceae					
*Cissampelos mucronata*	Herbal	*N. nigricollis*	SVH	—	[82]
Moraceae					
*Ficus platyphylla*	Folium	*N. nigricollis*	SVH	—	[82]
Olacaceae					
*Ximenia americana*	Folium	*N. nigricollis*	SVH	—	[82]
Pedaliaceae					
*Ceratotheca sesamoides*	Herbal	*N. nigricollis*	SVH	—	[82]
Peraceae					
*Clutia pulchella*	Radix	*N. nigricollis*	SVH	—	[82]
Phyllanthaceae					
*Flueggea virosa *(syn.* Securinega virosa*)	Radix	*N. nigricollis*	SVH	—	[82]
Pinaceae					
*Cedrus deodara*	Bark	*N. n. karachiensis*	PLA_2_	—	[84]
*Pinus roxburghii* ^#^	Oleoresin	*N. n. karachiensis*	PLA_2_	—	[84]
Poaceae					
*Cymbopogon schoenanthus*	Radix	*N. nigricollis*	SVH	—	[82]
Primulaceae					
*Maesa lanceolata* ^#^	Cortex	*N. nigricollis*	SVH	—	[82]
Rhamnaceae					
*Ziziphus mucronata*	Radix	*N. nigricollis*	SVH	—	[82]
*Ziziphus spina-christi*	Cortex	*N. nigricollis*	SVH	—	[82]
Rubiaceae					
*Crossopteryx febrifuga*	Cortex	*N. nigricollis*	SVH	—	[82]
*Pentanisia prunelloides*	Radix	*N. nigricollis*	SVH	—	[82]
*Pentas zanzibarica*	Folium	*N. nigricollis*	SVH	—	[82]
*Rubia cordifolia* ^#^	Stem				
Rutaceae					
*Citrus limon* ^#^	Fruit	*N. n. karachiensis*	PLA_2_	—	[84]
*Zanthoxylum capense*	Radix	*N. nigricollis*	SVH	—	[82]
Sapindaceae					
*Paullinia pinnata*	Folium	*N. nigricollis*	SVH	—	[82]
*Sapindus mukorossi*	Fruit	*N. n. karachiensis*	PLA_2_	—	[84]
Solanaceae					
*Nicotiana rustica*	Leaf	*N. nigricollis*	Proteolytic	—	[87]
*Schwenckia americana*	Folium	*N. nigricollis*	SVH	—	[82]
Thymelaeaceae					
*Gnidia anthylloides*	Radix	*N. nigricollis*	SVH	—	[82]
*Gnidia kraussiana*	Radix	*N. nigricollis*	SVH	—	[82]
*Gnidia splendens*	Radix	*N. nigricollis*	SVH	—	[82]
Verbenaceae					
*Lantana trifolia*	Cortex	*N. nigricollis*	SVH	—	[82]
Vitaceae					
*Cissus populnea*	Stem	*N. nigricollis*	SVH	—	[82]
Zingiberaceae					
*Zingiber officinale* ^#^	Rhizome	*N. n. karachiensis*	PLA_2_	—	[84]
Zygophyllaceae					
*Fagonia cretica*	Leaf, stem	*N. n. karachiensis*	PLA_2_	—	[84]

ND = information not described in the work; PLA_2_ = snake venom phospholipase A_2_; SVH = snake venom hyaluronidase. ^#^Vegetal species with related folk use as antiophidic agents, as showed in Table 1. ^*∗*^Studies where inhibitory activity was assessed only by preincubation of venom with extract (see Section 4.1 for details).

**Table 3:** List of medicinal plants with inhibitory potential against local effects induced by *Bothrops *snakes.

Plant name	Part used	Snake venom	Inhibited activities		Reference(s)
			*In vitro*	*In vivo*	
Acanthaceae					
*Justicia pectoralis* ^#^	Leaf	*B. jararaca*	—	Hemorrhage^*∗*^	[33]
Amaranthaceae					
*Blutaparon portulacoides*	Aerial parts	*B. jararacussu*	—	Edema	[109]
Anacardiaceae					
*Anacardium excelsum*	Leaf, twig	*B. asper*	PLA_2_	—	[111]
Annonaceae					
*Ephedranthus columbianus*	Leaf, twig	*B. asper*	PLA_2_	—	[111]
*Sapranthus isae*	Leaf, twig	*B. asper*	PLA_2_	—	[111]
Apocynaceae					
*Allamanda cathartica* ^#^	Branch, leaf, stem	*B. atrox*	—	Hemorrhage^*∗*^	[124]
*Fernaldia pandurata *(syn.* Mandevilla velutina*)	Leaf, stem, subterranean system	*B. alternatus, B. jararacussu, B. moojeni, B. pirajai*	PLA_2_	Edema^*∗*^, hemorrhage^*∗*^, myotoxicity^*∗*^	[125]
*Tabernaemontana catharinensis*	Root bark	*B. jararacussu*	Myotoxicity	Myotoxicity^*∗∗*^	[126]
Araceae					
*Dracontium croatii* ^#^	Rhizome	*B. asper*	—	Edema^*∗*^	[127]
*Philodendron megalophyllum* ^#^	Liana, vine	*B. atrox, B. jararaca*	PLA_2_	Edema^*∗*^, hemorrhage^*∗*^	[33, 128]
*Philodendron tripartitum* ^#^	Branch, leaf	*B. atrox*	—	Hemorrhage^*∗*^	[124]
Asteraceae					
*Chaptalia nutans*	Leaf	*B. asper*	—	Edema	[129]
*Eclipta prostrata *(syn.* Eclipta alba*)^*#*^	Aerial parts	*B. jararaca, B. jararacussu*	Myotoxicity	Myotoxicity	[130]
*Mikania glomerata*	Leaf	*B. jararaca*	—	Edema^*∗*^, hemorrhage^*∗*^, peritonitis	[131, 132]
*Neurolaena lobata* ^#^	Branch, leaf, stem	*B. atrox*	—	Hemorrhage^*∗*^	[124]
*Pseudelephantopus spicatus* ^#^	Whole plant	*B. atrox*	—	Hemorrhage^*∗*^	[124]
Bignoniaceae					
*Fridericia chica *(syn.* Arrabidaea chica*)	Leaf	*B. atrox*	—	Edema	[133]
*Tabebuia aurea*	Stem bark	*B. neuwiedi*	H_2_O_2_ production by peritoneal macrophages	Edema, hemorrhage^*∗*^, myonecrosis^*∗*^, peritonitis	[110]
*Tabebuia rosea* ^#^	Stem bark	*B. asper, B. atrox*	PLA_2_	Edema^*∗∗*^, Hemorrhage^*∗*^	[124, 127, 134]
Bixaceae					
*Bixa orellana* ^#^	Branch, leaf	*B. asper, B. atrox*	PLA_2_	Edema^*∗∗*^, hemorrhage^*∗*^	[124, 127, 134]
Boraginaceae					
*Cordia verbenacea*	Leaf	*B. jararacussu*	—	Edema^*∗*^	[135]
Clusiaceae					
*Clusia fluminensis*	Fruit	*B. jararaca*	Proteolytic	Hemorrhage^*∗∗*^	[136]
Combretaceae					
*Combretum leprosum*	Root	*B. jararaca, B. jararacussu*	Collagenase, myotoxicity, PLA_2_, proteolytic	Edema, hemorrhage^*∗*^, myotoxicity^*∗∗*^	[99]
Connaraceae					
*Connarus favosus* ^#^	Bark	*B. atrox, B. jararaca*	PLA_2_, proteolytic	Edema^*∗*^, hemorrhage^*∗∗∗*^	[33, 115, 128]
Costaceae					
*Costus lasius* ^#^	ND	*B. atrox*	PLA_2_	—	[134]
*Costus spicatus*	Leaf	*B. atrox*	—	Edema^*∗*^, hyperalgesia^*∗*^	[137]
Crassulaceae					
*Bryophyllum pinnatum *(syn.* Kalanchoe pinnata*)^*#*^	Leaf	*B. jararaca*	PLA_2_	Edema, hemorrhage	[138]
*Kalanchoe laciniata *(syn.* Kalanchoe brasiliensis*)^*#*^	Leaf	*B. jararaca*	PLA_2_	Edema, hemorrhage^*∗∗∗*^	[33, 138]
Dicranaceae					
*Dicranum frigidum*	Whole plant	*B. asper*	PLA_2_	—	[111]
Dilleniaceae					
*Davilla elliptica*	Leaf	*B. jararaca*	—	Hemorrhage^*∗*^	[139]
Euphorbiaceae					
*Croton urucurana*	Stem bark	*B. jararaca*	—	Hemorrhage^*∗*^	[140]
*Hevea nitida*	Leaf, twig	*B. asper*	PLA_2_	—	[111]
*Jatropha gossypiifolia* ^#^	Leaf	*B. erythromelas, B. jararaca*	PLA_2_, proteolytic, SVH	Edema, hemorrhage, myotoxicity	[96, 119]
*Jatropha mollissima* ^#^	Leaf	*B. erythromelas, B. jararaca*	—	Edema, hemorrhage, myotoxicity, peritonitis	[141]
Fabaceae					
*Abarema cochliacarpos*	Stem bark	*B. leucurus*	—	Decreased motor function, edema, hyperalgesia, myotoxicity	[101]
*Brownea ariza*	Bark, leaf	*B. asper*	PLA_2_, proteolytic	—	[142]
*Brownea *rosa-de-monte^#^	Leaf, stem bark	*B. asper, B. atrox*	PLA_2_, proteolytic	Edema^*∗∗∗*^, hemorrhage^*∗∗∗*^	[124, 127, 134, 143]
*Cassia fistula* ^#^	Seed	*B. jararaca*	—	Hemorrhage^*∗*^	[33]
*Dipteryx alata*	Bark	*B. jararacussu*	Myotoxicity	—	[144]
*Pentaclethra macroloba* ^#^	Bark	*B. alternatus, B. asper, B. atrox, B. jararaca, B. jararacussu, B. moojeni, B. neuwiedi, B. pirajai*	PLA_2_	Edema^*∗*^, hemorrhage^*∗*^, myotoxicity^*∗*^	[145]
*Plathymenia reticulata* ^#^	Bark	*B. atrox, B. jararaca*	PLA_2_	Edema^*∗*^, hemorrhage^*∗*^	[33, 128]
*Schizolobium parahyba*	Leaf	*B. alternatus, B. moojeni, B. pauloensis*	PLA_2_	Hemorrhage^*∗*^, myotoxicity^*∗∗∗*^	[146, 147]
*Senna dariensis* ^#^	Whole plant	*B. atrox*	PLA_2_	Hemorrhage^*∗*^	[124, 134]
Heliconiaceae					
*Heliconia curtispatha* ^#^	Rhizome	*B. asper, B. atrox*	PLA_2_, proteolytic	Edema^*∗∗∗*^, hemorrhage^*∗*^	[124, 127, 134, 148]
*Heliconia latispatha*	Rhizome	*B. asper*	PLA_2_, proteolytic	—	[148]
*Heliconia wagneriana*	Rhizome	*B. asper*	PLA_2_, proteolytic	—	[148]
Hymenophyllaceae					
Trichomanes elegans^*#*^	Whole plant	*B. asper, B. atrox*	PLA_2_	Edema^*∗∗∗*^, hemorrhage^*∗*^	[124, 127, 134]
Hypericaceae					
*Hypericum brasiliense*	Whole plant	*B. jararaca*	Proteolytic	Edema^*∗*^, hemorrhage^*∗*^	[149]
Icacinaceae					
*Casimirella ampla *(syn.* Humirianthera ampla*)	Root	*B. atrox, B. jararaca, B. jararacussu*	Myotoxicity, PLA2, proteolytic	Edema^*∗∗∗*^, hemorrhage^*∗∗*^, myotoxicity^*∗*^	[102]
Lamiaceae					
*Marsypianthes chamaedrys* ^#^	Inflorescence, leaf	*B. atrox*	PLA_2_	Peritonitis	[108]
*Peltodon radicans*	Flower, leaf, stem	*B. atrox*	—	Edema	[150]
Lauraceae					
*Aniba parviflora *(syn*. Aniba fragrans*)^*#*^	Bark, leaf	*B. atrox, B. jararaca*	PLA_2_	Edema^*∗*^, hemorrhage^*∗*^	[33, 128]
Loasaceae					
*Nasa speciosa* (syn. *Loasa speciosa*)	Leaf	*B. asper*	—	Edema	[129]
Loganiaceae					
*Strychnos pseudoquina*	Leaf	*B. jararaca*	—	Hemorrhage^*∗*^	[139]
*Strychnos xinguensis* ^#^	ND	*B. atrox*	PLA_2_	—	[134]
Loranthaceae					
*Struthanthus orbicularis* ^#^	Branch, leaf	*B. asper, B. atrox*	PLA_2_	Edema^*∗∗*^, hemorrhage^*∗*^	[124, 127, 134]
Magnoliaceae					
*Magnolia espinalii *(syn.* Talauma espinalii*)	Leaf, twig	*B. asper*	PLA_2_	—	[111]
*Magnolia guatapensis *(syn.* Dugandiodendron guatapense*)	Leaf, twig	*B. asper*	PLA_2_	—	[111]
*Magnolia hernandezii *(syn.* Talauma hernandezii*)	Leaf, twig	*B. asper*	PLA_2_	—	[111]
*Magnolia yarumalensis *(syn.* Dugandiodendron yarumalense*)	Leaf, twig	*B. asper*	PLA_2_	—	[111]
Malpighiaceae					
*Byrsonima crassa*	Leaf	*B. jararaca*	—	Hemorrhage^*∗*^	
Malvaceae					
*Pachira glabra* (syn. *Bombacopsis glabra*)	Root bark	*B. pauloensis*	—	Hemorrhage	[151]
Melastomataceae					
*Bellucia dichotoma* ^#^	Bark	*B. atrox, B. jararaca*	PLA_2_	Edema^*∗∗∗*^, hemorrhage^*∗∗*^	[33, 98, 128, 152]
*Mouriri pusa*	Leaf	*B. jararaca*	—	Hemorrhage^*∗*^	[139]
Meliaceae					
*Carapa guianensis*	Leaf, twig	*B. asper*	PLA_2_	—	[111]
*Cedrela odorata*	Leaf, twig	*B. asper*	PLA_2_	—	[111]
*Swietenia humilis*	Leaf, twig	*B. asper*	PLA_2_	—	[111]
*Swietenia macrophylla*	Leaf, twig	*B. asper*	PLA_2_	—	[111]
*Swietenia mahagoni*	Leaf, twig	*B. asper*	PLA_2_	—	[111]
Menispermaceae					
*Cissampelos pareira* ^#^	Leaf	*B. asper*	—	Hemorrhage^*∗*^	[153]
Moraceae					
*Brosimum guianense*	Leaf	*B. atrox*	—	Hemorrhage^*∗*^, pain^*∗*^	[154]
*Castilla elastica* ^#^	Branch, leaf, stem	*B. atrox*	—	Hemorrhage^*∗*^	[124]
*Ficus nymphaeifolia* ^#^	Branch, leaf, stem	*B. asper, B. atrox*	—	Edema^*∗∗*^, hemorrhage^*∗*^	[124, 127]
Musaceae					
*Musa × paradisíaca* ^#^	Exudate	*B. jararacussu*	PLA_2_	Hemorrhage^*∗∗*^, myonecrosis^*∗∗*^	[155]
Myrtaceae					
*Myrcia guianensis*	Leaf	*B. jararaca*	PLA_2_	Hemorrhage^*∗*^	[156]
Passifloraceae					
*Passiflora quadrangularis* ^#^	Branch, leaf	*B. atrox*	—	Hemorrhage^*∗*^	[124]
Piperaceae					
*Piper arboreum* ^#^	Branch, leaf	*B. atrox*	PLA_2_	Hemorrhage^*∗*^	[124, 134]
*Piper pulchrum* ^#^	Leaf, branch, stem	*B. atrox*	—	Hemorrhage^*∗*^	[124]
Polypodiaceae					
*Pleopeltis percussa* ^#^	Branch, leaf, stem, whole plant	*B. asper, B. atrox*	PLA_2_, proteolytic	Edema^*∗∗*^, hemorrhage^*∗*^	[124, 127, 134]
Rubiaceae					
*Gonzalagunia panamensis* ^#^	Branch, leaf, stem	*B. asper, B. atrox*	PLA_2_	Edema^*∗∗*^, hemorrhage^*∗*^	[124, 127, 134]
*Randia aculeata* ^#^	Fruit	*B. asper*	—	Myotoxicity	[78]
*Uncaria tomentosa*	Root	*B. asper*	—	Edema	[129]
Rutaceae					
*Citrus limon* ^#^	Ripe fruit	*B. asper, B. atrox*	—	Edema^*∗∗∗*^, hemorrhage^*∗*^	[124, 127]
*Murraya paniculata* ^#^	Leaf, twig	*B. asper*	PLA_2_	—	[111]
Salicaceae					
*Casearia grandiflora* ^#^	Leaf	*B. moojeni, B. neuwiedi*	PLA_2_	Myotoxicity^*∗*^	[157]
*Casearia sylvestris* ^#^	Leaf	*B. asper, B. jararacussu, B. moojeni, B. neuwiedi, B. pirajai*	Myonecrosis, neuromuscular blockade	Edema^*∗*^, hemorrhage^*∗*^, myotoxicity^*∗*^	[158–160]
Sapindaceae					
*Billia hippocastanum*	Leaf, twig	*B. asper*	PLA_2_	—	[111]
*Cupania americana*	Leaf, twig	*B. asper*	PLA_2_	—	[111]
*Sapindus saponaria*	*In vitro* cultivated callus, leaf, twig	*B. alternatus, B. asper, B. jararacussu, B. moojeni*	PLA_2_	Hemorrhage^*∗*^	[111, 161]
*Serjania erecta*	Aerial parts	*B. jararacussu*	PLA_2_	Edema^*∗*^, hemorrhage^*∗*^, myotoxicity^*∗*^	[162]
Siparunaceae					
*Siparuna thecaphora* ^#^	Branch, leaf, stem	*B. atrox*	—	Hemorrhage^*∗*^	[124]
Solanaceae					
*Capsicum annuum *(syn.* Capsicum frutescens*)^*#*^	Ripe fruit	*B. atrox*	—	Hemorrhage^*∗*^	[124]
Urticaceae					
*Urera baccifera*	Leaf	*B. asper*	—	Edema	[129]
Velloziaceae					
*Vellozia squamata *(syn.* Vellozia flavicans*)	Leaf	*B. jararacussu*	Neuromuscular blockade and cell damage	—	[163]
Zingiberaceae					
*Curcuma longa* ^#^	Rhizome	*B. alternatus*	—	Edema, hemorrhage, necrosis	[164]
*Renealmia alpinia* ^#^	Leaf, rhizome	*B. asper, B. atrox*	PLA_2_, proteolytic	Edema^*∗∗*^, hemorrhage	[107, 127, 134, 165, 166]

ND = information not described in the work; PLA_2_ = snake venom phospholipase A_2_; H_2_O_2_: hydrogen peroxide. ^#^Vegetal species with related folk use as antiophidic agents, as showed in Table 1. ^*∗*^Studies where inhibitory activity was assessed only by preincubation of venom with extract (see Section 4.1 for details). ^*∗∗*^Active in preincubation tests but inactive or only poorly active when extract was used independently of venom (pre-, co-, or posttreatment protocols). ^*∗∗∗*^Active in preincubation tests and when used independently of venom (pre-, co-, or posttreatment protocols).

**Table 4:** List of medicinal plants with inhibitory potential against local effects induced by *Bitis *snakes.

Plant name	Part used	Snake venom	Inhibited activities		Reference(s)
			*In vitro*	*In vivo*	
Amaranthaceae					
*Pupalia lappacea*	Herbal	*B. arietans*	SVH	—	[82]
Amaryllidaceae					
*Crinum jagus*	Bulb	*B. arietans*	—	Myotoxicity^*∗*^	[167]
Anacardiaceae					
*Lannea acida*	Cortex	*B. arietans*	PLA_2_, proteolytic, SVH	—	[82]
*Sclerocarya birrea*	Cortex	*B. arietans*	PLA_2_, proteolytic, SVH	—	[82]
*Spondias mombin* ^#^	Cortex, radix	*B. arietans*	PLA_2_, proteolytic, SVH	—	[82]
Annonaceae					
*Annona senegalensis* ^#^	Cortex	*B. arietans*	PLA_2_, proteolytic, SVH	—	[82]
Apocynaceae					
*Strophanthus speciosus*	Radix	*B. arietans*	SVH	—	[82]
Araliaceae					
*Polyscias fulva*	Cortex	*B. arietans*	SVH	—	[82]
Bignoniaceae					
*Kigelia africana*	Cortex	*B. arietans*	PLA_2_, SVH	—	[82]
Bixaceae					
*Cochlospermum tinctorium*	Radix	*B. arietans*	PLA_2_, proteolytic, SVH	—	[82]
Capparaceae					
*Capparis tomentosa*	Radix	*B. arietans*	PLA_2_, proteolytic, SVH	—	[82]
Colchicaceae					
*Gloriosa superba* ^#^	Radix	*B. arietans*	SVH	—	[82]
Combretaceae					
*Combretum molle* ^#^	Folium	*B. arietans*	PLA_2_, proteolytic, SVH	—	[82]
*Guiera senegalensis*	Radix	*B. arietans*	PLA_2_, proteolytic, SVH	—	[82]
Ebenaceae					
*Diospyros mespiliformis*	Cortex	*B. arietans*	PLA_2_, proteolytic, SVH	—	[82]
Euphorbiaceae					
*Alchornea laxiflora*	Cortex	*B. arietans*	PLA_2_, proteolytic, SVH	—	[82]
Fabaceae					
*Bauhinia thonningii*	Cortex, radix	*B. arietans*	PLA_2_, proteolytic, SVH	—	[82]
*Bobgunnia madagascariensis *(syn.* Swartzia madagascariensis*)	Folium, radix	*B. arietans*	PLA_2_, proteolytic, SVH	—	[82]
*Burkea africana*	Cortex	*B. arietans*	PLA_2_, proteolytic, SVH	—	[82]
*Dichrostachys cinerea*	Folium	*B. arietans*	PLA_2_, proteolytic, SVH	—	[82]
*Entada africana*	Radix	*B. arietans*	SVH	—	[82]
*Parkia biglobosa*	Cortex	*B. arietans*	PLA_2_, proteolytic, SVH	—	[82]
*Stylosanthes erecta*	Folium	*B. arietans*	SVH	—	[82]
*Tamarindus indica* ^#^	Cortex, folium	*B. arietans*	PLA_2_, proteolytic, SVH	—	[82]
Hypericaceae					
*Psorospermum corymbiferum*	Cortex, radix	*B. arietans*	PLA_2_, proteolytic, SVH	—	[82]
Hypoxidaceae					
*Molineria capitulata *(syn.* Curculigo recurvata*)	Folium	*B. arietans*	SVH	—	[82]
Lamiaceae					
*Rotheca myricoides *(syn.* Clerodendrum myricoides*)	Cortex	*B. arietans*	SVH	—	[82]
*Teucrium kraussii*	Aerial parts, cortex	*B. arietans*	SVH	—	[82]
*Volkameria glabra *(syn.* Clerodendrum glabrum*)	Cortex	*B. arietans*	PLA_2_, proteolytic, SVH	—	[82]
Lauraceae					
*Cassytha filiformis*	Herbal	*B. arietans*	SVH	—	[82]
Loganiaceae					
*Strychnos decussata*	Radix	*B. arietans*	Proteolytic	—	[82]
*Strychnos innocua*	Folium	*B. arietans*	Proteolytic, SVH	—	[82]
Malvaceae					
*Dombeya quinqueseta*	Cortex	*B. arietans*	PLA_2_, proteolytic, SVH	—	[82]
*Grewia mollis*	Cortex, folium, radix	*B. arietans*	PLA_2_, proteolytic, SVH	—	[82]
*Sterculia setigera*	Cortex	*B. arietans*	PLA_2_, SVH	—	[82]
*Waltheria indica*	Radix	*B. arietans*	PLA_2_, proteolytic, SVH	—	[82]
Menispermaceae					
*Cissampelos mucronata*	Herbal	*B. arietans*	Proteolytic, PLA_2_	—	[82]
Moraceae					
*Ficus platyphylla*	Folium	*B. arietans*	PLA_2_, SVH	—	[82]
Olacaceae					
*Ximenia americana*	Folium	*B. arietans*	PLA_2_, proteolytic, SVH	—	[82]
Phyllanthaceae					
*Flueggea virosa *(syn*. Securinega virosa*)	Radix	*B. arietans*	PLA_2_, proteolytic, SVH	—	[82]
Primulaceae					
*Maesa lanceolata* ^#^	Cortex	*B. arietans*	PLA_2_, proteolytic, SVH	—	[82]
Rhamnaceae					
*Ziziphus mucronata*	Radix	*B. arietans*	PLA_2_, proteolytic, SVH	—	[82]
*Ziziphus spina-christi*	Cortex	*B. arietans*	PLA_2_, proteolytic, SVH	—	[82]
Rubiaceae					
*Crossopteryx febrifuga*	Cortex	*B. arietans*	PLA_2_, SVH	—	[82]
*Pentanisia prunelloides*	Radix	*B. arietans*	PLA_2_, proteolytic, SVH	—	[82]
*Pentas zanzibarica*	Folium	*B. arietans*	PLA_2_	—	[82]
Rutaceae					
*Zanthoxylum capense*	Radix	*B. arietans*	PLA_2_, proteolytic	—	[82]
Sapindaceae					
*Paullinia pinnata*	Folium, radix	*B. arietans*	PLA_2_, proteolytic, SVH	—	[82]
Solanaceae					
*Schwenckia americana*	Folium	*B. arietans*	SVH	—	[82]
Verbenaceae					
*Lantana trifolia*	Cortex	*B. arietans*	PLA_2_, SVH	—	[82]
Vitaceae					
*Cissus populnea*	Stem	*B. arietans*	SVH	—	[82]

PLA_2_ = snake venom phospholipase A_2_; SVH = snake venom hyaluronidase. ^#^Vegetal species with related folk use as antiophidic agents, as showed in Table 1. ^*∗*^Studies where inhibitory activity was assessed only by preincubation of venom with extract (see Section 4.1 for details).

**Table 5:** List of medicinal plants with inhibitory potential against local effects induced by *Daboia/Vipera *snakes.

Plant name	Part used	Snake venom	Inhibited activities		Reference(s)
			*In vitro*	*In vivo*	
Anacardiaceae					
*Anacardium occidentale* ^#^	Bark	*D. russelli*	PLA_2_, proteolytic, SVH	Edema, hemorrhage, myotoxicity	[170]
*Mangifera indica* ^#^	Stem bark	*D. russelii*	LAAO, PLA_2_, SVH, proteolytic	Edema^*∗*^, hemorrhage^*∗*^, myotoxicity^*∗*^	[171]
Apocynaceae					
*Hemidesmus indicus* ^#^	Root	*D. russelli*	—	Hemorrhage	[172]
*Tylophora indica* ^#^	Leaf, root	*D. russelli*	PLA_2_	Hemorrhage	[85]
Aristolochiaceae					
*Aristolochia bracteolata* ^#^	Leaf, root	*D. russelli*	PLA_2_	Hemorrhage	[85]
*Aristolochia indica* ^#^	Root	*D. russelii*	LAAO, proteolytic	—	[173]
Asteraceae					
*Pluchea indica* ^#^	Root	*D. russelli*	—	Hemorrhage	[172]
Euphorbiaceae					
*Acalypha indica* ^#^	Leaf	*D. r. russelli*	—	Hemorrhage, necrosis	[174]
Fabaceae					
*Butea monosperma* ^#^	Stem bark	*D. russelii*	SVH	Hemorrhage^*∗*^	[175]
*Mimosa pudica* ^#^	Root	*D. russelii*	Proteolytic, SVH	—	[91]
*Tamarindus indica* ^#^	Seed	*D. r. siamensis, D. russelii*	LAAO, PLA_2_, SVH, proteolytic	Edema^*∗*^, hemorrhage^*∗*^, myotoxicity^*∗*^	[176, 177]
Lamiaceae					
*Leucas aspera* ^#^	Leaf, root	*D. russelii*	PLA_2_	Hemorrhage	[85]
*Vitex negundo* ^#^	Root	*D. russelii*	—	Edema, hemorrhage	[178]
Loganiaceae					
*Strychnos *nux-vomica^#^	Seed	*D. russelii*	PLA_2_	Hemorrhage^*∗*^	[95]
Moraceae					
*Morus alba* ^#^	Leaf	*D. russelii*	Proteolytic, SVH	Edema^*∗*^, hemorrhage^*∗*^, myotoxicity^*∗*^	[179]
Phyllanthaceae					
*Phyllanthus emblica *(syn.* Emblica officinalis*)^*#*^	Root	*D. russelii*	—	Edema, hemorrhage	[178]
Piperaceae					
*Piper longum* ^#^	Fruit	*D. russelii*	Hemorrhage	Edema, hemorrhage, myotoxicity, necrosis	[104]
Rubiaceae					
*Ophiorrhiza mungos* ^#^	Root	*D. russelii*	Hemorrhage	—	[180]
Salvadoraceae					
*Azima tetracantha*	Leaf	*D. russelii*	SVH	—	[181]
Vitaceae					
*Vitis vinifera*	Seed	*D. russelii*	Proteolytic, SVH	Edema^*∗*^, hemorrhage^*∗*^, myonecrosis^*∗*^	[182]

LAAO = L-amino acid oxidase; PLA_2_ = snake venom phospholipase A_2_; SVH = snake venom hyaluronidase. ^#^Vegetal species with related folk use as antiophidic agents, as showed in Table 1. ^*∗*^Studies where inhibitory activity was assessed only by preincubation of venom with extract (see Section 4.1 for details).

**Table 6:** List of medicinal plants with inhibitory potential against local effects induced by *Lachesis *snakes.

Plant name	Part used	Snake venom	Inhibited activities		Reference(s)
			*In vitro*	*In vivo*	
Apocynaceae					
*Fernaldia pandurata *(syn*. Mandevilla velutina*)	Root	*L. muta*	Proteolytic, PLA_2_	Hemorrhage^*∗*^	[188]
Asteraceae					
*Eclipta prostrata *(syn.* Eclipta alba*)^#^	Aerial parts, root	*L. muta*	Myotoxicity, proteolytic, PLA_2_	Hemorrhage^*∗*^, myotoxicity	[130, 188]
*Mikania glomerata*	Root	*L. muta*	Proteolytic, PLA_2_	—	[188]
Erythroxylaceae					
*Erythroxylum ovalifolium*	Stem	*L. muta*	Proteolytic, PLA_2_	Edema^*∗∗∗*^, hemorrhage^*∗∗∗*^	[189]
*Erythroxylum subsessile*	Stem	*L. muta*	Proteolytic, PLA_2_	Edema^*∗∗∗*^, hemorrhage^*∗∗∗*^	[189]
Euphorbiaceae					
*Jatropha elliptica*	Root, stem	*L. muta*	Proteolytic, PLA_2_	Hemorrhage^*∗*^	[188]
Fabaceae					
*Pentaclethra macroloba* ^#^	Bark	*L. muta*	—	Hemorrhage^*∗*^	[145]
*Stryphnodendron adstringens *(syn.* Stryphnodendron barbatimam*)	Root	*L. muta*	Proteolytic, PLA_2_	Hemorrhage^*∗*^	[188]
Melastomataceae					
*Miconia albicans*	Stem	*L. muta*	Proteolytic, PLA_2_	Hemorrhage^*∗*^	[188]
*Miconia fallax*	Stem	*L. muta*	Proteolytic, PLA_2_	Hemorrhage^*∗*^	[188]
*Miconia sellowiana*	ND	*L. muta*	Proteolytic, PLA_2_	Hemorrhage^*∗*^	[188]
*Tibouchina stenocarpa*	Root	*L. muta*	Proteolytic, PLA_2_	Hemorrhage^*∗*^	[188]
Salicaceae					
*Casearia sylvestris* ^#^	Root	*L. muta*	Proteolytic	Hemorrhage^*∗*^	[188]
Sapotaceae					
*Manilkara subsericea*	Leaf, stem	*L. muta*	Proteolytic, PLA_2_	Edema^*∗∗*^, hemorrhage^*∗∗*^	[100]

ND = information not described in the work; PLA_2_ = snake venom phospholipase A_2_. ^#^Vegetal species with related folk use as antiophidic agents, as showed in Table 1. ^*∗*^Studies where inhibitory activity was assessed only by preincubation of venom with extract (see Section 4.1 for details). ^*∗∗*^Active in preincubation tests but inactive or only poorly active when extract was used independently of venom (pre-, co-, or posttreatment protocols). ^*∗∗∗*^Active in preincubation tests and when used independently of venom (pre-, co-, or posttreatment protocols).

**Table 7:** List of medicinal plants with inhibitory potential against local effects induced by *Crotalus *snakes.

Plant name	Part used	Snake venom	Inhibited activities		Reference(s)
			*In vitro*	*In vivo*	
Apocynaceae					
*Fernaldia pandurata *(syn.* Mandevilla velutina*)	Leaf, stem, subterranean system	*C. d. terrificus*	PLA_2_	Edema^*∗*^, myotoxicity^*∗*^	[125]
*Mandevilla illustris*	Subterranean system	*C. d. terrificus*	PLA_2_	—	[190]
Asteraceae					
*Eclipta prostrata *(syn.* Eclipta alba*)^*#*^	Aerial parts	*C. d. terrificus*	Myotoxicity	Myotoxicity^*∗*^	[11]
Bignoniaceae					
*Fridericia chica *(syn.* Arrabidaea chica*)	Leaf	*C. d. ruruima*	—	Edema	[133]
Fabaceae					
*Pentaclethra macroloba* ^#^	Bark	*C. atrox*	—	Hemorrhage^*∗*^	[145]
*Schizolobium parahyba*	Leaf	*C. d. terrificus*	PLA_2_	Edema^*∗*^	[146, 147]
Musaceae					
*Musa* × *paradisiaca*^#^	Exudate	*C. d. terrificus*	PLA_2_	—	[155]
Rubiaceae					
*Randia aculeata* ^#^	Fruit	*C. simus*	—	Myotoxicity	[78]
Sapindaceae					
*Sapindus saponaria*	*In vitro* cultivated callus	*C. d. terrificus*	PLA_2_	—	[161]

PLA_2_ = snake venom phospholipase A_2_. ^#^Vegetal species with related folk use as antiophidic agents, as showed in Table 1. ^*∗*^Studies where inhibitory activity was assessed only by preincubation of venom with extract (see Section 4.1 for details).

**Table 8:** List of medicinal plants with inhibitory potential against local effects induced by other snakes.

Plant name	Part used	Snake venom	Inhibited activities		Reference(s)
			*In vitro*	*In vivo*	
Amaryllidaceae					
*Crinum jagus*	Bulb	*Echis ocellatus*	Hemorrhage	Myotoxicity^*∗∗*^	[167, 191]
Asteraceae					
*Artemisia absinthium*	Aerial parts	*Montivipera xanthina*	—	Edema	[192]
*Mikania laevigata*	Leaf	*Philodryas olfersii*	Inflammation, myotoxicity	—	[193]
Fabaceae					
*Albizia lebbeck* ^#^	Seed	*Echis carinatus*	Proteolytic, SVH	Hemorrhage, myotoxicity	[194]
*Mimosa pudica* ^#^	Root	*Bungarus caeruleus, Echis carinatus*	PLA_2_, proteolytic, SVH	Edema^*∗*^	[91, 92]
*Parkia biglobosa*	Stem bark	*Echis ocellatus*	Cytotoxicity against muscle cells, hemorrhage	—	[93]
*Pentaclethra macroloba* ^#^	Bark	*Calloselasma rhodostoma*	—	Hemorrhage^*∗*^	[145]
*Senna auriculata *(syn.* Cassia auriculata*)	Leaf	*Echis carinatus*	PLA_2_, proteolytic, SVH	Edema^*∗∗∗*^, hemorrhage^*∗∗∗*^, myotoxicity^*∗∗∗*^	[195]
Malvaceae					
*Hibiscus aethiopicus*	Whole plant	*Echis carinatus, Echis ocellatus*	Cytotoxicity against muscle cells, hemorrhage	Edema^*∗∗∗*^, hemorrhage^*∗∗∗*^	[196, 197]
Salvadoraceae					
*Azima tetracantha*	Leaf	*Bungarus caeruleus*	PLA_2_	—	[181]
Vitaceae					
*Vitis vinifera*	Seed	*Echis carinatus*	Proteolytic, SVH	Edema^*∗*^, hemorrhage^*∗*^, myotoxicity^*∗*^	[198]

PLA_2_ = snake venom phospholipase A_2_; SVH = snake venom hyaluronidase. ^#^Vegetal species with related folk use as antiophidic agents, as showed in Table 1. ^*∗*^Studies where inhibitory activity was assessed only by preincubation of venom with extract (see Section 4.1 for details). ^*∗∗*^Active in preincubation tests but inactive or only poorly active when extract was used independently of venom (pre-, co-, or posttreatment protocols). ^*∗∗∗*^Active in preincubation tests and when used independently of venom (pre-, co-, or posttreatment protocols).

## References

[1] Gutiérrez J. M., Burnouf T., Harrison R. A. (2015). A call for incorporating social research in the global struggle against snakebite. *PLoS Neglected Tropical Diseases*.

[2] Kasturiratne A., Wickremasinghe A. R., de Silva N. (2008). The global burden of snakebite: a literature analysis and modelling based on regional estimates of envenoming and deaths. *PLoS Medicine*.

[3] Gutiérrez J. M., Warrell D. A., Williams D. J. (2013). The need for full integration of snakebite envenoming within a global strategy to combat the neglected tropical diseases: the way forward. *PLoS Neglected Tropical Diseases*.

[4] Harrison R. A., Hargreaves A., Wagstaff S. C., Faragher B., Lalloo D. G. (2009). Snake envenoming: a disease of poverty. *PLoS Neglected Tropical Diseases*.

[5] Warrell D. A. (2012). Venomous animals. *Medicine*.

[6] Guimarães C. L. S., Moreira-Dill L. S., Fernandes R. S. (2014). Biodiversity as a source of bioactive compounds against snakebites. *Current Medicinal Chemistry*.

[7] Kang T. S., Georgieva D., Genov N. (2011). Enzymatic toxins from snake venom: structural characterization and mechanism of catalysis. *The FEBS Journal*.

[8] GutiΘrrez J. M., Lomonte B. (1989). Local tissue damage induced by Bothrops snake venoms. A review. *Memorias do Instituto Butantan*.

[9] Gutiérrez J. M., León G., Burnouf T. (2011). Antivenoms for the treatment of snakebite envenomings: the road ahead. *Biologicals*.

[10] León G., Herrera M., Segura Á., Villalta M., Vargas M., Gutiérrez J. M. (2013). Pathogenic mechanisms underlying adverse reactions induced by intravenous administration of snake antivenoms. *Toxicon*.

[11] Mors W. B., do Nascimento M. C., Parente J. P., da Silva M. H., Melo P. A., Suarez-Kurtz G. (1989). Neutralization of lethal and myotoxic activities of south american rattlesnake venom by extracts and constituents of the plant *Eclipta prostrata* (Asteraceae). *Toxicon*.

[12] Santhosh M. S., Hemshekhar M., Sunitha K. (2013). Snake venom induced local toxicities: plant secondary metabolites as an auxiliary therapy. *Mini-Reviews in Medicinal Chemistry*.

[13] Shabbir A., Shahzad M., Masci P., Gobe G. C. (2014). Protective activity of medicinal plants and their isolated compounds against the toxic effects from the venom of *Naja* (cobra) species. *Journal of Ethnopharmacology*.

[14] Dey A., de J. N. (2012). Phytopharmacology of antiophidian botanicals: A review. *International Journal of Pharmacology*.

[15] Gomes A., Das R., Sarkhel S. (2010). Herbs and herbal constituents active against snake bite. *Indian Journal of Experimental Biology*.

[16] Sulochana A., Raveendran D., Krishnamma A., Oommen O. (2015). Ethnomedicinal plants used for snake envenomation by folk traditional practitioners from Kallar forest region of South Western Ghats, Kerala, India. *Journal of Intercultural Ethnopharmacology*.

[17] Butt M. A., Ahmad M., Fatima A. (2015). Ethnomedicinal uses of plants for the treatment of snake and scorpion bite in Northern Pakistan. *Journal of Ethnopharmacology*.

[18] Dharmadasa R. M., Akalanka G. C., Muthukumarana P. R. M., Wijesekara R. G. S. (2016). Ethnopharmacological survey on medicinal plants used in snakebite treatments in Western and Sabaragamuwa provinces in Sri Lanka. *Journal of Ethnopharmacology*.

[19] Kala C. P. (2015). Herbal treatment for snakebites in Uttarakhand state of India. *Indian Journal of Natural Products and Resources*.

[20] Samy R. P., Thwin M. M., Gopalakrishnakone P., Ignacimuthu S. (2008). Ethnobotanical survey of folk plants for the treatment of snakebites in Southern part of Tamilnadu, India. *Journal of Ethnopharmacology*.

[21] Rajadurai M., Vidhya V. G., Ramya M., Bhaskar A. (2009). Ethno-medicinal plants used by the traditional healers of pachamalai hills, Tamilnadu, India. *Studies on Ethno-Medicine*.

[22] Sarkhel S. (2014). Ethnobotanical survey of folklore plants used in treatment of snakebite in Paschim Medinipur district, West Bengal. *Asian Pacific Journal of Tropical Biomedicine*.

[23] Penchalapratap G., Sudarsanam G., Pushpan R., Prasad G. P. (2010). Herbal remedies for snake bites in ethnic practices of Chittoor District, Andhra Pradesh. *Ancient Science of Life*.

[24] Singh A. K., Raghubanshi A. S., Singh J. S. (2002). Medical ethnobotany of the tribals of Sonaghati of Sonbhadra district, Uttar Pradesh, India. *Journal of Ethnopharmacology*.

[25] Kadel C., Jain A. K. (2008). Folklore claims on snakebite among some tribal communities of Central India. *Indian Journal of Traditional Knowledge*.

[26] Yesodharan K., Sujana K. A. (2007). Ethnomedicinal knowledge among Malamalasar tribe of Parambikulam wildlife sanctuary. *Indian Journal of Traditional Knowledge*.

[27] Coe F. G., Anderson G. J. (2005). Snakebite ethnopharmacopoeia of eastern Nicaragua. *Journal of Ethnopharmacology*.

[28] Prabu M., Kumuthakalavalli R. (2012). Folk remedies of medicinal plants for snake bites, scorpion stings and dog bites in Eastern Ghats of Kolli Hills, Tamil Nadu, India. *International Journal of Research in Ayurveda and Pharmacy*.

[29] Sanz-Biset J., Campos-de-la-Cruz J., Epiquién-Rivera M. A., Cañigueral S. (2009). A first survey on the medicinal plants of the Chazuta valley (Peruvian Amazon). *Journal of Ethnopharmacology*.

[30] Hasan M. N., Azam M. N. K., Ahmed M. N., Hirashima A. (2015). A randomized ethnomedicinal survey of snakebite treatment in southwestern parts of Bangladesh. *Journal of Traditional and Complementary Medicine*.

[31] Owuor B. O., Kisangau D. P. (2006). Kenyan medicinal plants used as antivenin: a comparison of plant usage. *Journal of Ethnobiology and Ethnomedicine*.

[32] Kadir M. F., Karmoker J. R., Alam M. R., Jahan S. R., Mahbub S., Mia M. M. K. (2015). Ethnopharmacological survey of medicinal plants used by traditional healers and indigenous people in Chittagong Hill Tracts, Bangladesh, for the treatment of snakebite. *Evidence-Based Complementary and Alternative Medicine*.

[33] De Moura V. M., Freitas De Sousa L. A., Cristina Dos-Santos M. (2015). Plants used to treat snakebites in Santarém, western Pará, Brazil: an assessment of their effectiveness in inhibiting hemorrhagic activity induced by Bothrops jararaca venom. *Journal of Ethnopharmacology*.

[34] Vásquez J., Alarcón J. C., Jiménez S. L. (2015). Main plants used in traditional medicine for the treatment of snake bites n the regions of the department of Antioquia, Colombia. *Journal of Ethnopharmacology*.

[35] Otero R., Fonnegra R., Jiménez S. L. (2000). Snakebites and ethnobotany in the northwest region of Colombia. Part I: traditional use of plants. *Journal of Ethnopharmacology*.

[36] Rahmatullah M., Hasan A., Parvin W. (2012). Medicinal plants and formulations used by the Soren clan of the Santal tribe in Rajshahi District, Bangladesh for treatment of various ailments. *African Journal of Traditional, Complementary and Alternative Medicines*.

[37] Rigat M., Vallès J., Dambrosio U., Gras A., Iglésias J., Garnatje T. (2015). Plants with topical uses in the Ripollès district (Pyrenees, Catalonia, Iberian Peninsula): Ethnobotanical survey and pharmacological validation in the literature. *Journal of Ethnopharmacology*.

[38] Jain A., Katewa S. S., Sharma S. K., Galav P., Jain V. (2011). Snakelore and indigenous snakebite remedies practiced by some tribals of Rajasthan. *Indian Journal of Traditional Knowledge*.

[39] Shukla A. N., Srinivastan S., Rawat A. K. S. (2010). An ethnobotanical study of medicinal plants of Rewa district, Madhya Pradesh. *Indian Journal of Traditional Knowledge*.

[40] Panghal M., Arya V., Yadav S., Kumar S., Yadav J. P. (2010). Indigenous knowledge of medicinal plants used by Saperas community of Khetawas, Jhajjar District, Haryana, India. *Journal of Ethnobiology and Ethnomedicine*.

[41] Khan A. V., Ahmed Q. U., Khan M. W., Khan A. A. (2014). Herbal cure for poisons and poisonous bites from Western Uttar Pradesh, India. *Asian Pacific Journal of Tropical Disease*.

[42] Sikdar M., Dutta U. (2008). Traditional phytotherapy among the Nath people of Assam. *Studies on Ethno-Medicine*.

[43] Khan M. A., Khan S. A., Quresh M. A. (2011). Ethnobotany of some useful plants of poonch valley azad kashmir. *Journal of Medicinal Plant Research*.

[44] Vásquez J., Jiménez S. L., Gómez I. C. (2013). Snakebites and ethnobotany in the eastern region of Antioquia, Colombia—the traditional use of plants. *Journal of Ethnopharmacology*.

[45] Sivasankari B., Anandharaj M., Gunasekaran P. (2014). An ethnobotanical study of indigenous knowledge on medicinal plants used by the village peoples of Thoppampatti, Dindigul district, Tamilnadu, India. *Journal of Ethnopharmacology*.

[46] Mebs D. (2000). Notes on the traditional use of plants to treat snake bite in northern Papua New Guinea. *Toxicon*.

[47] Agra M. D. F., Silva K. N., Basílio I. J. L. D., De Freitas P. F., Barbosa-Filho J. M. (2008). Survey of medicinal plants used in the region Northeast of Brazil. *Brazilian Journal of Pharmacognosy*.

[48] Ritter R. A., Monteiro M. V. B., Monteiro F. O. B. (2012). Ethnoveterinary knowledge and practices at Colares island, Pará state, eastern Amazon, Brazil. *Journal of Ethnopharmacology*.

[49] Murthy K. S., Sharma P. C., Kishore P. (1986). Tribal remedies for snakebite from Orissa. *Ancient Science of Life*.

[50] Di Stasi L. C., Hiruma-Lima C. A. (2002). *Plantas medicinais na Amazônia e na Mata Atlântica*.

[51] Belayneh A., Bussa N. F. (2014). Ethnomedicinal plants used to treat human ailments in the prehistoric place of Harla and Dengego valleys, eastern Ethiopia. *Journal of Ethnobiology and Ethnomedicine*.

[52] Ayyanar M., Ignacimuthu S. (2011). Ethnobotanical survey of medicinal plants commonly used by Kani tribals in Tirunelveli hills of Western Ghats, India. *Journal of Ethnopharmacology*.

[53] Haq F., Ahmad H., Alam M. (2011). Traditional uses of medicinal plants of Nandiar Khuwarr catchment (District Battagram), Pakistan. *Journal of Medicinal Plants Research*.

[54] Cheikhyoussef A., Shapi M., Matengu K., Ashekele H. M. (2011). Ethnobotanical study of indigenous knowledge on medicinal plant use by traditional healers in Oshikoto region, Namibia. *Journal of Ethnobiology and Ethnomedicine*.

[55] Jain A., Katewa S. S., Galav P. K., Sharma P. (2005). Medicinal plant diversity of Sitamata wildlife sanctuary, Rajasthan, India. *Journal of Ethnopharmacology*.

[56] Dutt H. C., Bhagat N., Pandita S. (2015). Oral traditional knowledge on medicinal plants in jeopardy among Gaddi shepherds in hills of northwestern Himalaya, J&K, India. *Journal of Ethnopharmacology*.

[57] Odonne G., Valadeau C., Alban-Castillo J., Stien D., Sauvain M., Bourdy G. (2013). Medical ethnobotany of the Chayahuita of the Paranapura basin (Peruvian Amazon). *Journal of Ethnopharmacology*.

[58] Jankovic M. (2014). Communication and shared information. *Philosophical Studies. An International Journal for Philosophy in the Analytic Tradition*.

[59] Hernandez M. R., Avila-Bello C. H., Morales-Mavil J. E. (2007). Etnobotánica y ecología de plantas utilizadas por tres curanderos contra la mordedura de serpiente en la región de Acayucan, Veracruz, México. *Boletin de la Sociedad Botanica de Mexico*.

[60] Rahmatullah M., Ferdausi D., Mollik M. A. H., Jahan R., Chowdhury M. H., Haque W. M. (2010). A survey of medicinal plants used by Kavirajes of Chalna area, Khulna district, Bangladesh. *African Journal of Traditional, Complementary and Alternative Medicines*.

[61] Giovannini P. (2015). Medicinal plants of the Achuar (Jivaro) of Amazonian Ecuador: ethnobotanical survey and comparison with other Amazonian pharmacopoeias. *Journal of Ethnopharmacology*.

[62] Karuppaiya P., Tsay H. S. (2015). Therapeutic values, chemical constituents and toxicity of Taiwanese Dysosma pleiantha: a review. *Toxicology Letters*.

[63] Kshirsagar R. D., Singh N. P. (2001). Some less known ethnomedicinal uses from Mysore and Coorg districts, Karnataka state, India. *Journal of Ethnopharmacology*.

[64] De Albuquerque U. P., Andrade L. D. H. C. (2002). Uso de recursos vegetais da caatinga: O caso do agreste do Estado de Pernambuco (Nordeste do Brasil). *Interciencia*.

[65] Cheikhyoussef A., Embashu W. (2013). Ethnobotanical knowledge on indigenous fruits in Ohangwena and Oshikoto regions in Northern Namibia. *Journal of Ethnobiology and Ethnomedicine*.

[66] Shah A., Bharati K. A., Ahmad J., Sharma M. (2015). New ethnomedicinal claims from Gujjar and Bakerwals tribes of Rajouri and Poonch districts of Jammu and Kashmir, India. *Journal of Ethnopharmacology*.

[67] Ong H. C., Nordiana M. (1999). Malay ethno-medico botany in Machang, Kelantan, Malaysia. *Fitoterapia*.

[68] Seebaluck R., Gurib-Fakim A., Mahomoodally F. (2015). Medicinal plants from the genus Acalypha (Euphorbiaceae): a review of their ethnopharmacology and phytochemistry. *Journal of Ethnopharmacology*.

[69] Sharma P., Devi U. (2013). Ethnobotanical uses of biofencing plants in Himachal Pradesh, Northwest Himalaya. *Pakistan Journal of Biological Sciences*.

[70] Coelho-Ferreira M. (2009). Medicinal knowledge and plant utilization in an Amazonian coastal community of Marudá, Pará State (Brazil). *Journal of Ethnopharmacology*.

[71] Oliveira F. C. S., Barros R. F. M., Moita Neto J. M. (2010). Medicinal plants used in rural communities from Oeiras Municipality, in the semi-arid region of Piauí State (PI), Brazil. *Revista Brasileira de Plantas Medicinais*.

[72] Crepaldi C. G., Campos J. L. A., Albuquerque U. P., Sales M. F. (2016). Richness and ethnobotany of the family Euphorbiaceae in a tropical semiarid landscape of Northeastern Brazil. *South African Journal of Botany*.

[73] Murugesan M., Balasubramaniam V., Arthi H. (2005). Ethno medical knowledge of plants used by Irula Tribes, Chengal Combai, the Nilgiris, Tamilnadu. *Ancient Science of Life*.

[74] Nsuala B. N., Enslin G., Viljoen A. (2015). “Wild cannabis”: a review of the traditional use and phytochemistry of Leonotis leonurus. *Journal of Ethnopharmacology*.

[75] Swamy M. K., Sinniah U. R. (2015). A Comprehensive review on the phytochemical constituents and pharmacological activities of Pogostemon cablin Benth.: an Aromatic Medicinal Plant of Industrial Importance. *Molecules*.

[76] Gakuya D. W., Itonga S. M., Mbaria J. M., Muthee J. K., Musau J. K. (2013). Ethnobotanical survey of biopesticides and other medicinal plants traditionally used in Meru central district of Kenya. *Journal of Ethnopharmacology*.

[77] Hazarika T. K., Lalramchuana, Nautiyal B. P. (2012). Studies on wild edible fruits of Mizoram, India used as ethno-medicine. *Genetic Resources and Crop Evolution*.

[78] Gallardo-Casas C. A., Guevara-Balcázar G., Morales-Ramos E. (2012). Ethnobotanic study of Randia aculeata (Rubiaceae) in Jamapa, Veracruz, Mexico, and its anti-snake venom effects on mouse tissue. *Journal of Venomous Animals and Toxins Including Tropical Diseases*.

[79] Xia L., Guo Q., Tu P., Chai X. (2014). The genus Casearia: a phytochemical and pharmacological overview. *Phytochemistry Reviews*.

[80] Molander M., Saslis-Lagoudakis C. H., Jäger A. K., Rønsted N. (2012). Cross-cultural comparison of medicinal floras used against snakebites. *Journal of Ethnopharmacology*.

[81] Félix-Silva J., Giordani R. B., Silva-Jr A. A. D., Zucolotto S. M., Fernandes-Pedrosa M. D. F. (2014). *Jatropha gossypiifolia* L. (Euphorbiaceae): a review of traditional uses, phytochemistry, pharmacology, and toxicology of this medicinal plant. *Evidence-Based Complementary and Alternative Medicine*.

[82] Molander M., Nielsen L., Søgaard S. (2014). Hyaluronidase, phospholipase A_2_ and protease inhibitory activity of plants used in traditional treatment of snakebite-induced tissue necrosis in Mali, DR Congo and South Africa. *Journal of Ethnopharmacology*.

[83] Thangavel N., Gupta J. K. (2007). Anti-inflammatory and anti-snake venom activity of Andrographis stenophylla leaf. *Asian Journal of Chemistry*.

[84] Asad M. H. H. B., Razi M. T., Durr-e-Sabih (2013). Anti-venom potential of Pakistani medicinal plants: inhibition of anticoagulation activity of Naja naja karachiensis toxin. *Current Science*.

[85] Sakthivel G., Dey A., Nongalleima K. (2013). In vitro and in vivo evaluation of polyherbal formulation against Russell's viper and cobra venom and screening of bioactive components by docking studies. *Evidence-Based Complementary and Alternative Medicine*.

[86] Soni P., Bodakhe S. H. (2014). Antivenom potential of ethanolic extract of Cordia macleodii bark against Naja venom. *Asian Pacific Journal of Tropical Biomedicine*.

[87] Ibrahim M. A., Aliyu A. B., Abusufiyanu A., Bashir M., Sallau A. B. (2011). Inhibition of naja nigricolis (Reinhardt) venom protease activity by Luffa egyptiaca (Mill) and Nicotiana rustica (Linn) extracts. *Indian Journal of Experimental Biology*.

[88] Gopi K., Renu K., Sannanaik Vishwanath B., Jayaraman G. (2015). Protective effect of Euphorbia hirta and its components against snake venom induced lethality. *Journal of Ethnopharmacology*.

[89] Rathnakar Reddi K. V. N., Rajesh S. S., Narendra K. (2014). In vitro anti-venom potential of various Jatropha extracts on neutralizing cytotoxic effect induced by phospholipase A2 of crude venom from Indian cobra (Naja naja). *Bangladesh Journal of Pharmacology*.

[90] Mahanta M., Mukherjee A. K. (2001). Neutralisation of lethality, myotoxicity and toxic enzymes of Naja kaouthia venom by Mimosa pudica root extracts. *Journal of Ethnopharmacology*.

[91] Girish K. S., Mohanakumari H. P., Nagaraju S., Vishwanath B. S., Kemparaju K. (2004). Hyaluronidase and protease activities from Indian snake venoms: Neutralization by Mimosa pudica root extract. *Fitoterapia*.

[92] Meenatchisundaram S., Priyagrace S., Vijayaraghavan R., Velmurugan A., Parameswari G., Michael A. (2009). Antitoxin activity of Mimosa pudica root extracts against Naja naja and Bangarus caerulus venoms. *Bangladesh Journal of Pharmacology*.

[93] Asuzu I. U., Harvey A. L. (2003). The antisnake venom activities of Parkia biglobosa (Mimosaceae) stem bark extract. *Toxicon*.

[94] Gopi K., Renu K., Jayaraman G. (2014). Inhibition of Naja naja venom enzymes by the methanolic extract of Leucas aspera and its chemical profile by GC-MS. *Toxicology Reports*.

[95] Chatterjee I., Chakravarty A. K., Gomes A. (2004). Antisnake venom activity of ethanolic seed extract of Strychnos nux vomica Linn. *Indian Journal of Experimental Biology*.

[96] Félix-Silva J., Souza T., Menezes Y. A. S. (2014). Aqueous leaf extract of *Jatropha gossypiifolia* L. (Euphorbiaceae) inhibits enzymatic and biological actions of *Bothrops jararaca* snake venom. *PLoS ONE*.

[97] WHO (2010). *Guidelines for the Production, Control and Regulation of Snake Antivenom Immunoglobulins*.

[98] Mourão De Moura V., Serra Bezerra A. N., Veras Mourão R. H. (2014). A comparison of the ability of Bellucia dichotoma Cogn. (Melastomataceae) extract to inhibit the local effects of Bothrops atrox venom when pre-incubated and when used according to traditional methods. *Toxicon*.

[99] Fernandes F. F. A., Tomaz M. A., El-Kik C. Z. (2014). Counteraction of Bothrops snake venoms by Combretum leprosum root extract and arjunolic acid. *Journal of Ethnopharmacology*.

[100] De Oliveira E. C., Fernandes C. P., Sanchez E. F., Rocha L., Fuly A. L. (2014). Inhibitory effect of plant Manilkara subsericea against biological activities of Lachesis muta snake venom. *BioMed Research International*.

[101] Saturnino-Oliveira J., Santos D. D. C., Guimarães A. G. (2014). Abarema cochliacarpos extract decreases the inflammatory process and skeletal muscle injury induced by bothrops leucurus venom. *BioMed Research International*.

[102] Strauch M. A., Tomaz M. A., Monteiro-Machado M. (2013). Antiophidic activity of the extract of the Amazon plant *Humirianthera ampla* and constituents. *Journal of Ethnopharmacology*.

[103] Houghton P. J., Osibogun I. M. (1993). Flowering plants used against snakebite. *Journal of Ethnopharmacology*.

[104] Shenoy P. A., Nipate S. S., Sonpetkar J. M., Salvi N. C., Waghmare A. B., Chaudhari P. D. (2013). Anti-snake venom activities of ethanolic extract of fruits of *Piper longum* L. (Piperaceae) against Russell's viper venom: characterization of piperine as active principle. *Journal of Ethnopharmacology*.

[105] Araújo S. D., de Souza A., Nunes F. P. B., Gonçalves L. R. C. (2007). Effect of dexamethasone associated with serum therapy on treatment of *Bothrops jararaca* venom-induced paw edema in mice. *Inflammation Research*.

[106] Patrão-Neto F. C., Tomaz M. A., Strauch M. A. (2013). Dexamethasone antagonizes the in vivo myotoxic and inflammatory effects of Bothrops venoms. *Toxicon*.

[107] Gómez-Betancur I., Benjumea D., Patiño A., Jiménez N., Osorio E. (2014). Inhibition of the toxic effects of Bothrops asper venom by pinostrobin, a flavanone isolated from Renealmia alpinia (Rottb.) MAAS. *Journal of Ethnopharmacology*.

[108] Magalhães A., Santos G. B. D., Verdam M. C. D. S. (2011). Inhibition of the inflammatory and coagulant action of *Bothrops atrox* venom by the plant species *Marsypianthes chamaedrys*. *Journal of Ethnopharmacology*.

[109] Pereira I. C., Barbosa A. M., Salvador M. J. (2009). Anti-inflammatory activity of Blutaparon portulacoides ethanolic extract against the inflammatory reaction induced by Bothrops jararacussu venom and isolated myotoxins BthTX-I and II. *Journal of Venomous Animals and Toxins Including Tropical Diseases*.

[110] Reis F. P., Senna Bonfa I. M., Cavalcante R. B. (2014). Tabebuia aurea decreases inflammatory, myotoxic and hemorrhagic activities induced by the venom of Bothrops neuwiedi. *Journal of Ethnopharmacology*.

[111] Pereañez J. A., Lobo-Echeverri T., Rojano B. (2010). Correlation of the inhibitory activity of phospholipase A2 snake venom and the antioxidant activity of Colombian plant extracts. *Brazilian Journal of Pharmacognosy*.

[112] Sunitha K., Hemshekhar M., Thushara R. M. (2015). Inflammation and oxidative stress in viper bite: an insight within and beyond. *Toxicon*.

[113] Saravia-Otten P., Gutiérrez J. M., Arvidson S., Thelestam M., Flock J.-I. (2007). Increased infectivity of Staphylococcus aureus in an experimental model of snake venom-induced tissue damage. *Journal of Infectious Diseases*.

[114] Jorge M. T., Ribeiro L. A. (1997). Infections in the bite site after envenoming by snakes of the Bothrops genus. *Journal of Venomous Animals and Toxins*.

[115] Silva T. P. D., Moura V. M. D., Souza M. C. S. D. (2016). Connarus favosus Planch.: An inhibitor of the hemorrhagic activity of Bothrops atrox venom and a potential antioxidant and antibacterial agent. *Journal of Ethnopharmacology*.

[116] Dehghani R., Sharif M. R., Moniri R., Sharif A., Kashani H. H. (2016). The identification of bacterial flora in oral cavity of snakes. *Comparative Clinical Pathology*.

[117] Hearn P., Miliya T., Hor S., Sar V., Turner P. (2015). Necrotizing fasciitis complicating snakebite in Cambodia. *IDCases*.

[118] Palappallil D. S. (2015). Pattern of use of antibiotics following snake bite in a tertiary care hospital. *Journal of Clinical and Diagnostic Research*.

[119] Félix-Silva J., Gomes J. A., Xavier-Santos J. B. (2017). Inhibition of local effects induced by Bothrops erythromelas snake venom: assessment of the effectiveness of Brazilian polyvalent bothropic antivenom and aqueous leaf extract of Jatropha gossypiifolia. *Toxicon*.

[120] Fernandes C. A. H., Cardoso F. F., Cavalcante W. G. L. (2015). Structural basis for the inhibition of a phospholipase A_2_-like toxin by caffeic and aristolochic acids. *PLoS ONE*.

[121] Srinivasa V., Sundaram M. S., Anusha S. (2014). Novel apigenin based small molecule that targets snake venom metalloproteases. *PLoS ONE*.

[122] Pereañez J. A., Patiño A. C., Núñez V., Osorio E. (2014). The biflavonoid morelloflavone inhibits the enzymatic and biological activities of a snake venom phospholipase A2. *Chemico-Biological Interactions*.

[123] Molander M., Staerk D., Mørck Nielsen H. (2015). Investigation of skin permeation, ex vivo inhibition of venom-induced tissue destruction, and wound healing of African plants used against snakebites. *Journal of Ethnopharmacology*.

[124] Otero R., Núñez V., Barona J. (2000). Snakebites and ethnobotany in the northwest region of Colombia—Part III: Neutralization of the haemorrhagic effect of Bothrops atrox venom. *Journal of Ethnopharmacology*.

[125] Biondo R., Pereira A. M. S., Marcussi S., Pereira P. S., França S. C., Soares A. M. (2003). Inhibition of enzymatic and pharmacological activities of some snake venoms and toxins by Mandevilla velutina (Apocynaceae) aqueous extract. *Biochimie*.

[126] Veronese E. L. G., Esmeraldino L. E., Trombone A. P. F. (2005). Inhibition of the myotoxic activity of Bothrops jararacussu venom and its two major myotoxins, BthTX-I and BthTX-II, by the aqueous extract of Tabernaemontana catharinensis A. DC. (Apocynaceae). *Phytomedicine*.

[127] Núñez V., Otero R., Barona J. (2004). Neutralization of the edema-forming, defibrinating and coagulant effects of *Bothrops asper* venom by extracts of plants used by healers in Columbia. *Brazilian Journal of Medical and Biological Research*.

[128] de Moura V. M., de Sousa L. A. F., de Oliveira R. B. (2013). Inhibition of the principal enzymatic and biological effects of the crude venom of Bothrops atrox by plant extracts. *Journal of Medicinal Plants Research*.

[129] Badilla B., Chaves F., Mora G., Poveda L. J. (2006). Edema induced by Bothrops asper (Squamata: Viperidae) snake venom and its inhibition by Costa Rican plant extracts. *Revista de Biologia Tropical*.

[130] Melo P. A., Nascimento M. C. D., Mors W. B., Suarez-Kurtz G. (1994). Inhibition of the myotoxic and hemorrhagic activities of crotalid venoms by *Eclipta prostrata* (*Asteraceae*) extracts and constituents. *Toxicon*.

[131] Mourao V. B., Giraldi G. M., Neves L. M. G. (2014). Anti-hemorrhagic effect of hydro-alcoholic extract of the leaves of *Mikania glomerata* in lesions induced by Bothrops jararaca venom in rats. *Acta Cirurgica Brasileira*.

[132] Motta Y. P., Sakate M., Nogueira R. M. B. (2014). Quantification of cytokines in serum and paw homogenate of experimental intoxication for venom of the Bothropoides jararaca in Wistar rats treated with antivenom and Mikania glomerata. *Arquivo Brasileiro de Medicina Veterinaria e Zootecnia*.

[133] Oliveira D. P. C., Borrás M. R. L., Ferreira L. C. D. L., López-Lozano J. L. (2009). Atividade antiinflamatória do extrato aquoso de *Arrabidaea chica* (Humb. & Bonpl.) B. Verl. sobre o edema induzido por venenos de serpentes amazônicas. *Revista Brasileira de Farmacognosia*.

[134] Otero R., Núñez V., Jiménez S. L. (2000). Snakebites and ethnobotany in the northwest region of Colombia. Part II: Neutralization of lethal and enzymatic effects of Bothrops atrox venom. *Journal of Ethnopharmacology*.

[135] Ticli F. K., Hage L. I. S., Cambraia R. S. (2005). Rosmarinic acid, a new snake venom phospholipase A2 inhibitor from Cordia verbenacea (Boraginaceae): Antiserum action potentiation and molecular interaction. *Toxicon*.

[136] De Oliveira E. C., Anholeti M. C., Domingos T. F. (2014). Inhibitory effect of the plant Clusia fluminensis against biological activities of Bothrops jararaca snake venom. *Natural Product Communications*.

[137] Picanço L. C. D. S., Bittencourt J. A. H. M., Henriques S. V. C. (2016). Pharmacological activity of Costus spicatus in experimental Bothrops atrox envenomation. *Pharmaceutical Biology*.

[138] Fernandes J. M., Félix-Silva J., Da Cunha L. M. (2016). Inhibitory effects of hydroethanolic leaf extracts of kalanchoe brasiliensis and kalanchoepinnata (crassulaceae) against local effects induced by bothropsjararaca snake venom. *PLoS ONE*.

[139] Nishijima C. M., Rodrigues C. M., Silva M. A., Lopes-Ferreira M., Vilegas W., Hiruma-Lima C. A. (2009). Anti-hemorrhagic activity of four brazilian vegetable species against Bothrops jararaca venom. *Molecules*.

[140] Esmeraldino L. E., Souza A. M., Sampaio S. V. (2005). Evaluation of the effect of aqueous extract of Croton urucurana Baillon (Euphorbiaceae) on the hemorrhagic activity induced by the venom of Bothrops jararaca, using new techniques to quantify hemorrhagic activity in rat skin. *Phytomedicine*.

[141] Gomes J. A. D. S., Félix-Silva J., Morais Fernandes J. (2016). Aqueous Leaf Extract of Jatropha mollissima (Pohl) Bail Decreases Local Effects Induced by Bothropic Venom. *BioMed Research International*.

[142] Mack-Wen V. L., Rico L. B., Alarc J. C., Perea J. A., Alarcón J. C. (2011). Inhibición in vitro del veneno de *Bothrops asper* con extractos etanólicos de *Brownea ariza* B. (Caesalpiniaceae). *Vitae*.

[143] Salazar M., Chérigo L., Acosta H., Otero R., Martínez-Luis S. (2014). Evaluation of anti-Bothrops asper venom activity of ethanolic extract of Brownea rosademonte leaves. *Acta Pharmaceutica*.

[144] Nazato V. S., Rubem-Mauro L., Vieira N. A. G. (2010). In vitro antiophidian properties of *Dipteryx alata* Vogel bark extracts. *Molecules*.

[145] Da Silva J. O., Coppede J. S., Fernandes V. C. (2005). Antihemorrhagic, antinucleolytic and other antiophidian properties of the aqueous extract from Pentaclethra macroloba. *Journal of Ethnopharmacology*.

[146] Vale L. H. F., Mendes M. M., Hamaguchi A., Soares A. M., Rodrigues V. M., Homsi-Brandeburgo M. I. (2008). Neutralization of pharmacological and toxic activities of Bothrops snake venoms by Schizolobium parahyba (Fabaceae) aqueous extract and its fractions. *Basic and Clinical Pharmacology and Toxicology*.

[147] Mendes M. M., Oliveira C. F., Lopes D. S. (2008). Anti-snake venom properties of Schizolobium parahyba (caesalpinoideae) aqueous leaves extract. *Phytotherapy Research*.

[148] Perea J. A., Jimenez S. L., Quintana J. C., Pereañez J. A. (2008). Inhibición de las actividades proteolítica, coagulante y hemolítica indirecta inducidas por el veneno de *Bothrops asper* por extractos etanólicos de tres especies de heliconias. *Vitae*.

[149] Assafim M., Coriolano E. C., Benedito S. E. (2011). *Hypericum brasiliense* plant extract neutralizes some biological effects of *Bothrops jararaca* snake venom. *Journal of Venom Research*.

[150] Costa H. N., Santos M. C., Alcântara A. F., Silva M. C., França R. C., Piló-Veloso D. (2008). Constituintes químicos e atividade antiedematogênica de Peltodon radicans (Lamiaceae). *Química Nova*.

[151] Mendes M. M., Vieira S. A. P. B., Gomes M. S. R. (2013). Triacontyl p-coumarate: an inhibitor of snake venom metalloproteinases. *Phytochemistry*.

[152] de Moura V. M., de Souza L. Y., da Costa Guimarães N. (2017). The potential of aqueous extracts of Bellucia dichotoma Cogn. (Melastomataceae) to inhibit the biological activities of Bothrops atrox venom: A comparison of specimens collected in the states of Pará and Amazonas, Brazil. *Journal of Ethnopharmacology*.

[153] Badilla B., Chaves F., Jiménez S., Rodríguez G., Poveda L. J. (2008). Effects of an extract of Cissampelos pareira on the hemorrhagic and proteolytic activities from Bothrops asper venom. *Pharmacognosy Magazine*.

[154] Bittencourt J. A. H. M., de Oliveira N. K. S., Cabral M. S. (2014). Antiophidian activity of Brosimum guianense (AUBL) Huber. *American Journal of Pharmacology and Toxicology*.

[155] Borges M. H., Alves D. L. F., Raslan D. S. (2005). Neutralizing properties of *Musa paradisiaca* L. (Musaceae) juice on phospholipase A_2_, myotoxic, hemorrhagic and lethal activities of crotalidae venoms. *Journal of Ethnopharmacology*.

[156] Sousa L. A. F., Moura V. M., Raposo J. D. A. (2013). The effect of the aqueous extract of Myrcia guianensis (AubL) DC and its fractions against the hemorrhagic activity of Bothrops jararaca venom. *Journal of Medicinal Plants Research*.

[157] Freitas F. G., Silva T. A., Oliveira F. (2005). Toxicidade aguda e propriedades antiofídicas do extrato aquoso de *Casearia grandiflora* (Flacourtiaceae): atividades fosfolipásica A_2_, miotóxica e letal de peçonhas de *B. moojeni* e *B. neuwiedi*. *Bioscience Journal*.

[158] Cintra-Francischinelli M., Silva M. G., Andréo-Filho N. (2008). Antibothropic action of casearia sylvestris Sw. (flacourtiaceae) extracts. *Phytotherapy Research*.

[159] Borges M. H., Soares A. M., Rodrigues V. M. (2001). Neutralization of proteases from Bothrops snake venoms by the aqueous extract from Casearia sylvestris (Flacourtiaceae). *Toxicon*.

[160] Da Silva S. L., Calgarotto A. K., Chaar J. S., Marangoni S. (2008). Isolation and characterization of ellagic acid derivatives isolated from Casearia sylvestris SW aqueous extract with anti-PLA2 activity. *Toxicon*.

[161] Da Silva M. L., Marcussi S., Fernandes R. S. (2012). Anti-snake venom activities of extracts and fractions from callus cultures of Sapindus saponaria. *Pharmaceutical Biology*.

[162] Fernandes R. S., Costa T. R., Marcussi S. (2011). Neutralization of pharmacological and toxic activities of Bothrops jararacussu snake venom and isolated myotoxins by Serjania erecta methanolic extract and its fractions. *Journal of Venomous Animals and Toxins Including Tropical Diseases*.

[163] Tribuiani N., da Silva A. M., Ferraz M. C. (2014). Vellozia flavicans Mart. ex Schult. hydroalcoholic extract inhibits the neuromuscular blockade induced by Bothrops jararacussu venom. *BMC Complementary and Alternative Medicine*.

[164] Melo M. M., Lúcia M., Habermehl G. G. (2007). Plant extracts for topic therapy of Bothrops alternatus envenomation. *Brazilian Journal of Pharmacognosy*.

[165] Fernández M., Ortiz W. F., Pereáñez J. A., Martínez D. (2010). Evaluación de las propiedades antiofídicas del extracto etanólico y fracciones obtenidas de Renealmia alpinia(Rottb) Mass (Zingiberaceae) cultivada in vitro. *Vitae*.

[166] Patiño A. C., López J., Aristizábal M., Quintana J. C., Benjumea D. (2012). Evaluation of the inhibitory effect of extracts from leaves of renealmia alpinia rottb. maas(Zingiberaceae) on the venom of bothrops asper (mapaná). *Biomedica*.

[167] Ode O. J., Asuzu I. U. (2006). The anti-snake venom activities of the methanolic extract of the bulb of Crinum jagus (Amaryllidaceae). *Toxicon*.

[168] Gutiérrez J. M., Rucavado A., Chaves F., Díaz C., Escalante T. (2009). Experimental pathology of local tissue damage induced by *Bothrops asper* snake venom. *Toxicon*.

[169] Paixão-Cavalcante D., Kuniyoshi A. K., Portaro F. C. V., da Silva W. D., Tambourgi D. V. (2015). African Adders: Partial Characterization of Snake Venoms from Three Bitis Species of Medical Importance and Their Neutralization by Experimental Equine Antivenoms. *PLoS Neglected Tropical Diseases*.

[170] Ushanandini S., Nagaraju S., Nayaka S. C., Kumar K. H., Kemparaju K., Girish K. S. (2009). The anti-ophidian properties of Anacardium occidentale bark extract. *Immunopharmacology and Immunotoxicology*.

[171] Dhananjaya B. L., Zameer F., Girish K. S., D'Souza C. J. M. (2011). Anti-venom potential of aqueous extract of stem bark of Mangifera indica L. against Daboia russellii (Russell's viper) venom. *Indian Journal of Biochemistry and Biophysics*.

[172] Alam M. I., Auddy B., Gomes A. (1996). Viper venom neutralization by Indian medicinal plant (Hemidesmus indicus and Pluchea indica) root extracts. *Phytotherapy Research*.

[173] Bhattacharjee P., Bhattacharyya D. (2013). Characterization of the aqueous extract of the root of *Aristolochia indica*: evaluation of its traditional use as an antidote for snake bites. *Journal of Ethnopharmacology*.

[174] Shirwaikar A., Rajendran K., Bodla R., Kumar C. D. (2004). Neutralization potential of *Viper russelli* russelli (Russell's viper) venom by ethanol leaf extract of *Acalypha indica*. *Journal of Ethnopharmacology*.

[175] Tarannum S., Mohamed R., Vishwanath B. S. (2012). Inhibition of testicular and Vipera russelli snake venom hyaluronidase activity by Butea monosperma (Lam) Kuntze stem bark. *Natural Product Research*.

[176] Maung K. M., Lynn Z. (2012). Effects of Tamarind (Tamarindus indicus Linn) seed extract on Russell's viper (Daboia russelli siamensis) venom. *Tropical Biomedicine*.

[177] Ushanandini S., Nagaraju S., Kumar K. H. (2006). The anti-snake venom properties of *Tamarindus indica* (leguminosae) seed extract. *Phytotherapy Research*.

[178] Alam M. I., Gomes A. (2003). Snake venom neutralization by Indian medicinal plants (*Vitex negundo* and *Emblica officinalis*) root extracts. *Journal of Ethnopharmacology*.

[179] Chandrashekara K. T., Nagaraju S., Usha Nandini S., Basavaiah, Kemparaju K. (2009). Neutralization of local and systemic toxicity of Daboia russelii venom by Morus alba plant leaf extract. *Phytotherapy Research*.

[180] Krishnan S. A., Dileepkumar R., Nair A. S., Oommen O. V. (2014). Studies on neutralizing effect of Ophiorrhiza mungos root extract against Daboia russelii venom. *Journal of Ethnopharmacology*.

[181] Janardhan B., Shrikanth V. M., Mirajkar K. K., More S. S. (2014). In vitro screening and evaluation of antivenom phytochemicals from Azima tetracantha Lam. leaves against bungarus caeruleus and Vipera russelli. *Journal of Venomous Animals and Toxins Including Tropical Diseases*.

[182] Mahadeswaraswamy Y. H., Devaraja S., Kumar M. S., Goutham Y. N. J., Kemparaju K. (2009). Inhibition of local effects of Indian Daboia/Vipera russelli venom by the methanolic extract of grape (Vitis vinifera L.) seeds. *Indian Journal of Biochemistry and Biophysics*.

[183] Mukherjee A. K., Kalita B., Mackessy S. P. (2016). A proteomic analysis of Pakistan Daboia russelii russelii venom and assessment of potency of Indian polyvalent and monovalent antivenom. *Journal of Proteomics*.

[184] Wüster W. (1998). The genus *Daboia* (Serpentes: Viperidae): Russell's viper. *Hamadryad*.

[185] Warrell D. A. (2010). Snake bite. *The Lancet*.

[186] Pla D., Sanz L., Molina-Sánchez P. (2013). Snake venomics of Lachesis muta rhombeata and genus-wide antivenomics assessment of the paraspecific immunoreactivity of two antivenoms evidence the high compositional and immunological conservation across Lachesis. *Journal of Proteomics*.

[187] Gutiérrez J. M. (2002). Comprendiendo los venenos de serpientes: 50 Años de investigaciones en América Latina. *Revista de Biologia Tropical*.

[188] de Paula R. C., Sanchez E. F., Costa T. R. (2010). Antiophidian properties of plant extracts against Lachesis muta venom. *Journal of Venomous Animals and Toxins Including Tropical Diseases*.

[189] De Oliveira E. C., Cruz R. A. S., Amorim N. D. M. (2016). Protective effect of the plant extracts of erythroxylum sp. against toxic effects induced by the venom of lachesis muta snake. *Molecules*.

[190] Biondo R., Soares A. M., Bertoni B. W., França S. C., Pereira A. M. S. (2004). Direct organogenesis of Mandevilla illustris (Vell) Woodson and effects of its aqueous extract on the enzymatic and toxic activities of Crotalus durissus terrificus snake venom. *Plant Cell Reports*.

[191] Ode O. J., Nwaehujor C. O., Onakpa M. M. (2010). Evaluation of antihaemorrhagic and antioxidant potentials of *Crinum jagus* bulb. *International Journal of Applied Biology and Pharmaceutical Technology*.

[192] Nalbantsoy A., Erel Ş. B., Köksal Ç., Göçmen B., Yildiz M. Z., Karabay Yavaşoĝlu N. Ü. (2013). Viper venom induced inflammation with Montivipera xanthina (Gray, 1849) and the anti-snake venom activities of Artemisia absinthium L. in rat. *Toxicon*.

[193] Collaço R. D. C. O., Cogo J. C., Rodrigues-Simioni L., Rocha T., Oshima-Franco Y., Randazzo-Moura P. (2012). Protection by *Mikania laevigata* (guaco) extract against the toxicity of *Philodryas olfersii* snake venom. *Toxicon*.

[194] Amog P. U., Manjuprasanna V. N., Yariswamy M. (2016). Albizia lebbeck seed methanolic extract as a complementary therapy to manage local toxicity of Echis carinatus venom in a murine model. *Pharmaceutical Biology*.

[195] Nanjaraj Urs A. N., Yariswamy M., Joshi V. (2014). Local and systemic toxicity of Echis carinatus venom: Neutralization by Cassia auriculata L. leaf methanol extract. *Journal of Natural Medicines*.

[196] Hasson S. S., Al-Balushi M. S., Said E. A. (2012). Neutralisation of local haemorrhage induced by the saw-scaled viper Echis carinatus sochureki venom using ethanolic extract of Hibiscus aethiopicus L. *Evidence-Based Complementary and Alternative Medicine*.

[197] Hasson S. S., Al-Jabri A. A., Sallam T. A., Al-Balushi M. S., Mothana R. A. A. (2010). Antisnake venom activity of hibiscus aethiopicus L. against echis ocellatus and naja n. nigricollis. *Journal of Toxicology*.

[198] Mahadeswaraswamy Y. H., Nagaraju S., Girish K. S., Kemparaju K. (2008). Local tissue destruction and procoagulation properties of Echis carinatus venom: Inhibition by Vitis vinifera seed methanol extract. *Phytotherapy Research*.

[199] Boldrini-França J., Corrêa-Netto C., Silva M. M. S. (2010). Snake venomics and antivenomics of Crotalus durissus subspecies from Brazil: Assessment of geographic variation and its implication on snakebite management. *Journal of Proteomics*.

[200] Santoro M. L., Sousa-E-Silva M. C. C., Gonçalves L. R. C. (1999). Comparison of the biological activities in venoms from three subspecies of the South American rattlesnake (Crotalus durissus terrificus, C. durissus cascavella and C. durissus collilineatus). *Comparative Biochemistry and Physiology - C Pharmacology Toxicology and Endocrinology*.

[201] Sangiorgio F., Sakate M., Nogueira R. M. B., Araújo J. P., Chavez-Olortegui C. (2008). Kinetics of venom and antivenom serum levels, clinical evaluation and therapeutic effectiveness in dogs inoculated with Crotalus durissus terrificus venom. *Journal of Venomous Animals and Toxins Including Tropical Diseases*.

[202] Hiremath V., Nanjaraj Urs A. N., Joshi V. (2016). Differential action of medically important Indian BIG FOUR snake venoms on rodent blood coagulation. *Toxicon*.

[203] Chen Q., Wang W., Li Q., Bai Y., Zou X., Wu Y. (2014). Effect of externally applied Jidesheng anti-venom on skin and soft-tissue necrosis after Chinese cobra bite: a retrospective study. *Journal of Traditional Chinese Medicine*.

